# Between *Raetia Secunda* and the dutchy of Bavaria: Exploring patterns of human movement and diet

**DOI:** 10.1371/journal.pone.0283243

**Published:** 2023-04-05

**Authors:** Maren Velte, Andrea Czermak, Andrea Grigat, Brigitte Haas-Gebhard, Anja Gairhos, Anita Toncala, Bernd Trautmann, Jochen Haberstroh, Bernd Päffgen, Kristin von Heyking, Sandra Lösch, Joachim Burger, Michaela Harbeck

**Affiliations:** 1 SNSB, Bavarian State Collection for Anthropology, Munich, Germany; 2 School of Archaeology, University of Oxford, Oxford, United Kingdom; 3 Bavarian State Archaeological Collection, Munich, Germany; 4 Bavarian Regional Office for the Care of Monuments, Munich & Regensburg, Germany; 5 Institute of Prehistoric and Prehistoric Archaeology, Ludwig Maximilian University, Munich, Germany; 6 Institute of Forensic Medicine, Department of Physical Anthropology, University of Bern, Bern, Switzerland; 7 Institute of Organismic and Molecular Evolution, Johannes Gutenberg University, Mainz, Germany; University of Padova: Universita degli Studi di Padova, ITALY

## Abstract

During the transition from Late Antiquity to the Middle Ages, the Roman Empire dissolved in the West and medieval empires were founded. There has been much discussion about the role that migration played in this transition. This is especially true for the formation of the Baiuvariian tribe and the founding of this tribal dukedom, which took place from the 5^th^ to the 6^th^ century in what is now Southern Bavaria (Germany). In this study, we aimed to determine the extent of immigration during the beginning of this transformation and to shed further light on its character. To achieve this goal, we analyzed stable isotope values of strontium, carbon, and nitrogen from the teeth and bones of over 150 human remains from Southern Germany, dating from around 500 AD. This group of individuals included women with cranial modifications (ACD) which can be found sporadically in the burial grounds of this period. Our results showed an above-average migration rate for both men and women in the second half of the 5^th^ century. They also indicate that a foreign background may also be assumed for the women with ACD. The demonstrably different origins of the immigrants from isotopically diverse regions, and the identification of local differences in detectable migration rate, as well as indication for different timing of residential changes, highlight the complexity of immigration processes and the need for more studies at the regional level.

## Introduction

The transition period between Late Antiquity to the Middle Ages had a substantial influence on the formation of today’s European settlements, and genetic structures in Europe (e.g., [1, 2]). During this time the western part of the Roman Empire disintegrated and was replaced by medieval kingdoms. Historical sources describe the migration of entire communities, such as the tribes of the Alemanni, the Ostrogoth, the Lombards, or the Huns. Although the ethnic attributions of these associations are now considered outdated [3], the period between the 3^rd^ and the 6^th^ century is still commonly referred to as the “Migration Period,” and the extent and nature of these migrations are still discussed today [3–5].

**Fig 1:**
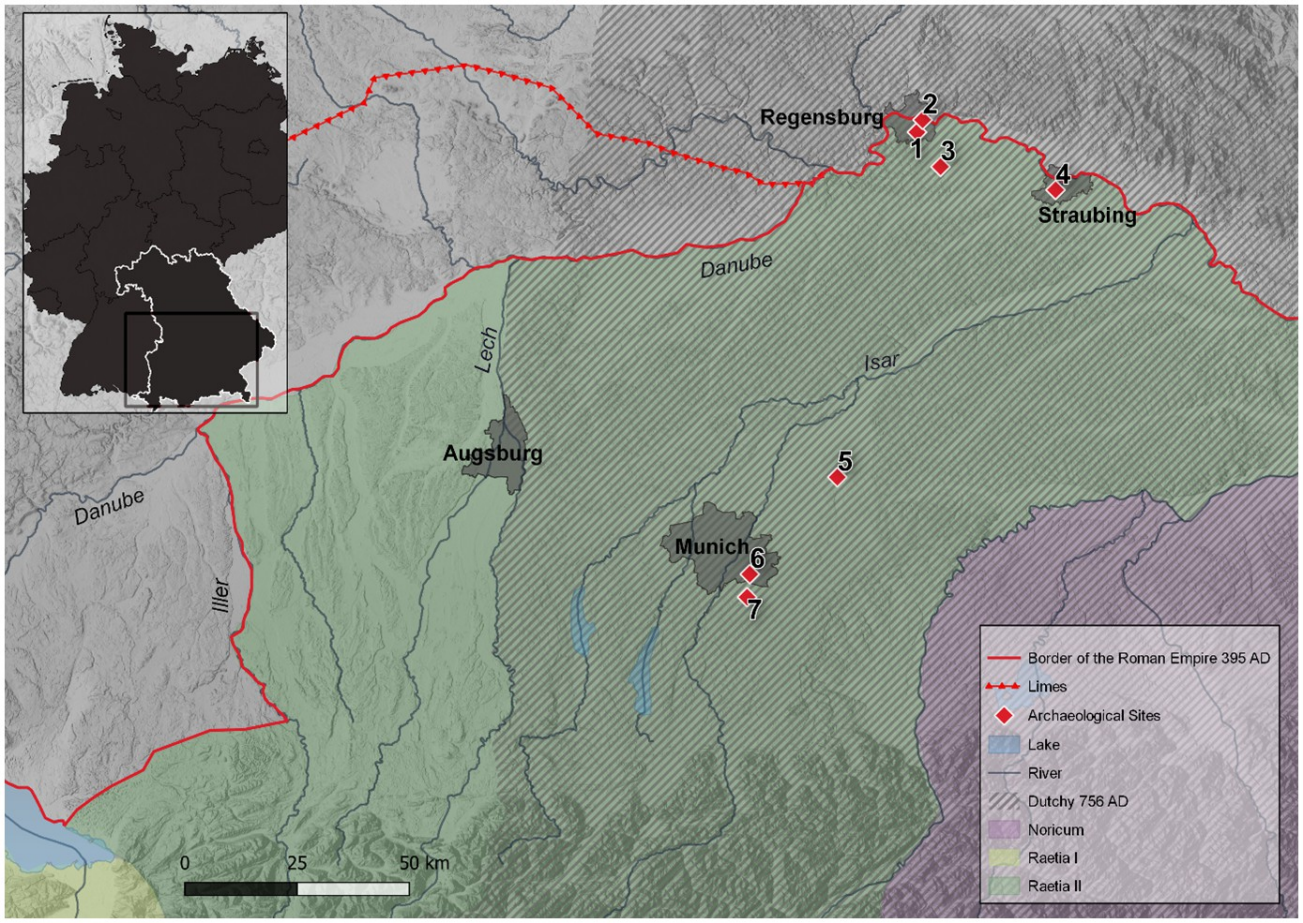
Map of the study area and location of archaeological sites. The study area is located south of the river Danube in the present German state of Bavaria. The region includes the part of Roman *Raetia II* that later became part of the first tribal duchy of the Baiuvarii. The Limes, the Roman-Germanic border established in the 1^st^ century AD, was abandoned in the middle of the 3^rd^ century, and the river boundaries of the Rhine, Iller, and Danube became the new imperial borders. Archaeological sites in the border zone: 1 Burgweinting A and B (BWA, BWB), 2 Irlmauth (IRM), 3 Alteglofsheim (AEH), 4 Straubing-Bajuwarenstraße (STB). Archaeological sites in the hinterland: 5 Altenerding (AED), 6 Munich-Perlach (PEL), 7 Unterhaching (UTH). (Map data: WMS Digital Terrain Model https://gdz.bkg.bund.de/index.php/default/wms-digitales-gelandemodell-gitterweite-200-m-wms-dgm200.html), Data licence Germany–attribution–Version 2.0 and GeoBasis-DE / BKG (2022) [7, 8]. Boundaries of provinces and course of the Limes shown as displayed in Harbeck et al. [9], dukedom drawn as displayed in Grollmann et al. [10]), (created with QGIS 3.18.3 Zürich).

During this time, the Roman province of *Raetia secunda (Raetia II)* dissolved in what is now Southern Bavaria, and the first tribal dukedom of the Baiuvarii emerged. In *Raetia II*, a functioning Roman administrative structure and border defenses are thought to have existed in the province until 476 AD [6]. However, throughout the 5^th^ century, and especially toward its end, there was a decline in Roman administrative and military structures and associated way of life. Historical sources for this region are lacking during this time, but archaeological findings indicate that the Roman lifestyle persisted well towards the end the 5^th^ century. Especially in larger settlements, such as the provincial capital Augusta Vindelicum (Augsburg), and also in border towns like Castra Regina (Regensburg) or Sorviodurum (Straubing), continuity from the late Roman period to the time of the first dukedom of the Baiuvarii can be observed (Fig 1).

Early studies attempting to explain the origins of the Baiuvarii argued that migration processes played a major role [11, 12], more recent studies suggest that local political factors, rather than migration, were key factors in their ethnogenesis [13, 14]. The extent of migration remains unclear and under debate. Whereas some authors argue for an increased immigration into the Roman province from the late 5^th^ century onwards [15], others contend that only marginal migration occurred during the second half of the 5^th^ century [16].

Most work on this topic has relied on the interpretation of grave goods as evidence of the non-local origins of some individuals, most of whom were women. This is primarily because brooches, belonging to the female costume, have been typologically associated with certain regions of Europe ([17, 18], but see [19, 20]). By contrast, the grave goods of men are rather uniform and thus allow for fewer inferences about mobility and migration [20]. Consequently, female migration is more likely to be detected, and male immigrants are likely underrepresented in the archaeological record.

This topic is further complicated by the fact that terms such as "migration" and "mobility" are not standardized and may cover a variety of different processes connected with residential change [21]. Variation includes the spatial extent of movement, the social context of movement or the number of people involved. In this study, we refer to migration as a long-term relocation of one or more persons who cross cultural and/or political boundaries, while mobility means individual or group movement across shorter distances that typically takes place within the own cultural and/or political region (see [21]).

Hakenbeck et al. [22] and Veeramah et al. [23] analyzed human remains instead of grave goods to address the question of mobility and migration for this region. Both studies reported evidence of migration predominantly for women and suggest an exogamous marriage system that involves women traveling over considerable distances. In particular, the hypothesis applies to a group of women with artificial cranial deformation (ACD). The custom of shaping the skull into an elongated shape in early childhood using bandages or other material is commonly associated with the Huns and their allies [24]. In Bavaria, ACD is mainly observed in women, and there is only disputed evidence for ACD in men or children. Veeramah et al. [23] demonstrated that in most cases, women with ACD buried in Bavaria showed a strong genetic resemblance to present-day South-Eastern European populations that was absent in individuals without ACD.

Hakenbeck et al. [22] determined carbon (δ^13^C) and nitrogen (δ^15^N) isotope values from the bone bulk collagen of various individuals from Early Medieval Bavaria. δ^15^N values are indicative of the trophic level of the consumer, and provide a proxy for the amount and type of dietary protein intake (e.g., [25]). δ^13^C values are generally used to indicate the relative contributions of C3 and C4 plant foods to the diet (e.g., [26]). Hakenbeck et al. [22] suggest that the average diet is mainly based on terrestrial C3 plants, with a large contribution of animal protein, but found little evidence of the usage of freshwater or marine resources. They also found evidence of an alternative diet in 5% of the individuals tested, most of whom were women with ACD and interpret this as a sign of migration.

Here, we build on the results of these prior studies by expanding the dataset to more than 100 additional individuals. We carried out strontium isotope (^87^Sr/^86^Sr) analyses on enamel to identify non-local individuals. ^87^Sr/^86^Sr varies substantially in regions with different bedrock geology and can thus serve as a geochemical signature. The natural distribution of strontium isotopes in soil, groundwater, and organisms in an ecosystem is primarily a function of the underlying geological system [27], and the contribution of exogenous sources, especially the input of atmospheric aerosols, may play a role [28, 29]. Bioavailable strontium isotopes that enter humans through the food chain are incorporated in the mineral structure of the skeletal system and display a blend of the strontium isotopes abundant in the habitat. Individuals showing a geochemical signature outside the local range of their burial site can be considered to be of non-local origin (e.g., [30]).

In addition, besides δ^15^N and δ^13^C from bone collagen, we additionally analyzed δ^13^C and δ^15^N in incremental dentine on a cohort of individuals, mainly composed of women with ACD and individuals for which a deviant diet in childhood was previously suggested. Whereas bone is remodeled throughout life, teeth formed during childhood or adolescence are barely remodeled and thus provide an isotopic archive of nutrition in early life. Accordingly, the analysis of dentine in comparison with bone samples allows for the detection of changes in dietary intake [31, 32], which can also provide indications of mobile behavior in some cases (e.g., [33, 34], see also [21]).

## Material

A total of 171 human skeletal samples were analyzed from seven archaeological sites in Southern Germany (Bavaria). The majority of samples dated to about 500 AD, according to archaeological or ^14^C dating (see [23, 35] for further details). About half of the selected burial sites are located near the border of the former Roman province; the other half corresponds to the hinterland.

Cemeteries vary in size and duration of use (Table 1), with the occurrence of two general types. Smaller to medium-sized burial groups came into use in the middle, or towards the end, of the 5^th^ century and were abandoned in the middle of the 6^th^ century. Large cemeteries, containing a substantially higher number of individuals ("Reihengräberfelder"), appeared at the same time and were in use until the middle of the 7^th^ century. The burial sites selected for this study captured this variety. If possible, all skeletons from smaller cemeteries were sampled. From large cemeteries, burials dating to around 500 AD were selected.

**Table 1:** Archeological sites investigated in this study.

Nr.	Name [Literature]	Total N of burials	Included n of burials	Period of use	Settlement zone	Specifics
1	Burgweinting A (BWA) [36]	15	15	Late 5^th^ to early 6^th^ centuries	Border	Small necropolis
1	Burgweinting B (BWB) [36]	19	19	Late 5^th^ to early 6^th^ centuries	Border	Small necropolis
2	Irlmauth (IRM) [37]	29* (+?)	16	First half of the 6^th^ century?	Border	Small to medium necropolis
3	Alteglofsheim (AEH) [38]	1* (+?)	1	Late 5^th^ to early 6^th^ centuries	Ambiguous	Poorly documented single finding of an individual with ACD
4	Straubing-Bajuwarenstraße (STB) [39]	Over 800	33	Mid-5^th^ to mid-7^th^ centuries	Border	Large cemetery
5	Altenerding (AED) [40, 41]	Over 1400	46	Mid-5^th^ to mid-7^th^ centuries	Hinterland	Large cemetery
6	Munich-Perlach (PEL) [42]	32* (+max 45)	29	Late 5^th^ to early 6^th^ centuries	Hinterland	Small to medium necropolis
7	Unterhaching (UTH) [43]	10* (+?)	10	Late 5^th^ to early 6^th^ centuries	Hinterland	Small necropolis including some individuals with remarkable grave goods indicating a higher social status

Including the total numbers of burials (N) and individuals studied (n).

*not completely excavated, maximum number of buried individuals can (+max N) or cannot (+?) be estimated,? = uncertain, see Fig 1 for locations of the cemeteries. Cent. = century, ACD = artificial cranial deformation.

Isotopic data from bone collagen and/or the enamel of some individuals were available from previous studies [9, 22, 42, 44–46] (see S1.1 Table in S1 Text for detailed list). To complement isotopic data from these studies the following samples were selected:

(1) Enamel samples from various teeth of 94 individuals for strontium isotope analysis: Preferably the second permanent molar (M2) was sampled to avoid the influence of breastfeeding. If M2 was not available, a tooth with a similar mineralization period was chosen (e.g., second premolar (P2), with approximate formation times at 2–8 and 2–7 years of age respectively. Only if the preferred teeth were not available other types of teeth were used. The mineralization of tooth enamel is completed at youth latest (third molar (M3): 16 years) ([47], see also [48]).
(2) Bone samples from 102 individuals for carbon and nitrogen isotope analysis: Preferably ribs without pathological bone formation were sampled. If ribs were not available cross sections of long bones were collected. Collagen in bone has a rather slow tissue turnover rate e.g., collagen from long bones reflects the average adult diet of the last 20–30 years before death [49]. The turnover of collagen in ribs is faster and thus reflects the average diet of a shorter time period before death [50]. However, since bone turnover rates generally decrease with age [49] it is difficult to estimate an exact timespan the measured isotope values actually reflect.
  Our own data combined with data from literature resulted in bone collagen δ^13^C and δ^15^N values from a total of 166 individuals, and ^87^Sr/^86^Sr ratios from the enamel of 164 individuals.
(3) In addition, we analyzed δ^13^C and δ^15^N from bulk dentine of the roots of first molars (M1) from a subsample of 24 individuals to obtain information about nutrition during early childhood. Crown sections were excluded to minimize the effect of breastfeeding and weaning. Root dentine reflects the diet between the age of 3.5 and 9.5 (see S2.2.2 section in S2 Text). The selection of individuals was then narrowed down to individuals with at least one M1 with little caries. Subsequently 15 Individuals were selected by means of presence of ACD or (potentially) deviating isotopic ratios in bone or enamel. Also, nine other individuals without ACD or deviating isotopic ratios were randomly selected (S3.2.1 Table in S3 Text). Six of the 24 individuals had all three molars preserved which were additionally analyzed.

The complete data set is provided in detail in S1.1 and S1.3 Table in S1 Text.

## Methods

### Osteological examination

Human remains for this and previous studies [23, 45, 51, 52] were provided by the State Collection of Anthropology Munich. Osteological data were recorded following the guidelines of the State Collection [53]. The presence of ACD was evaluated by Trautmann et al. [51]. For detailed osteological data see S1.1 Table in S1 Text.

### Strontium isotope analysis

#### Sample preparation and analysis

Procedures used for strontium extraction in this and previous studies followed the protocol described by Toncala et al. [54]. Enamel was removed from dentine using a drill, sonicated in concentrated CH_2_O_2_, washed twice with double distilled water, and incinerated at 500°C. Subsequently, samples were dissolved in concentrated HNO_3_ (69%) at 100°C for at least 24 h. After the acid evaporated, the samples were dissolved in 6N HNO_3_ at 100°C for 20 min. Sr-resin SR-B25-S (Eichrom) was used to separate strontium from other elements. Strontium was then eluted from the matrix with 0.05N HNO_3_.

Mass spectrometry were carried out at the RiesKraterMuseum Nördlingen (ZERIN). For mass spectrometry, extracts were loaded onto wolfram single filaments and analyzed with a thermal ionization mass spectrometer (MC-TIMS, MAT 261, Finnigan). Each sample was measured once, counting the isotopes 19 times in three blocks. Possible isotope mass fractionations during the analysis were corrected by normalizing the ^88^Sr/^86^Sr ratio to 8.37521 [55]. Strontium standard SRM 987 (^87^Sr/^86^Sr 0.71034±0.00026; National Institute of Standards and Technology) was used for quality control. The certified reference value was corrected to a generally accepted value of 0.71025±0.00001 [56, 57]. The mean ^87^Sr/^86^Sr ratio of SRM 987 during the measurements was 0.71022±0.00005 (n = 25, 1σ).

#### Determination of biologically available ^87^Sr/^86^Sr ranges

**Fig 2:**
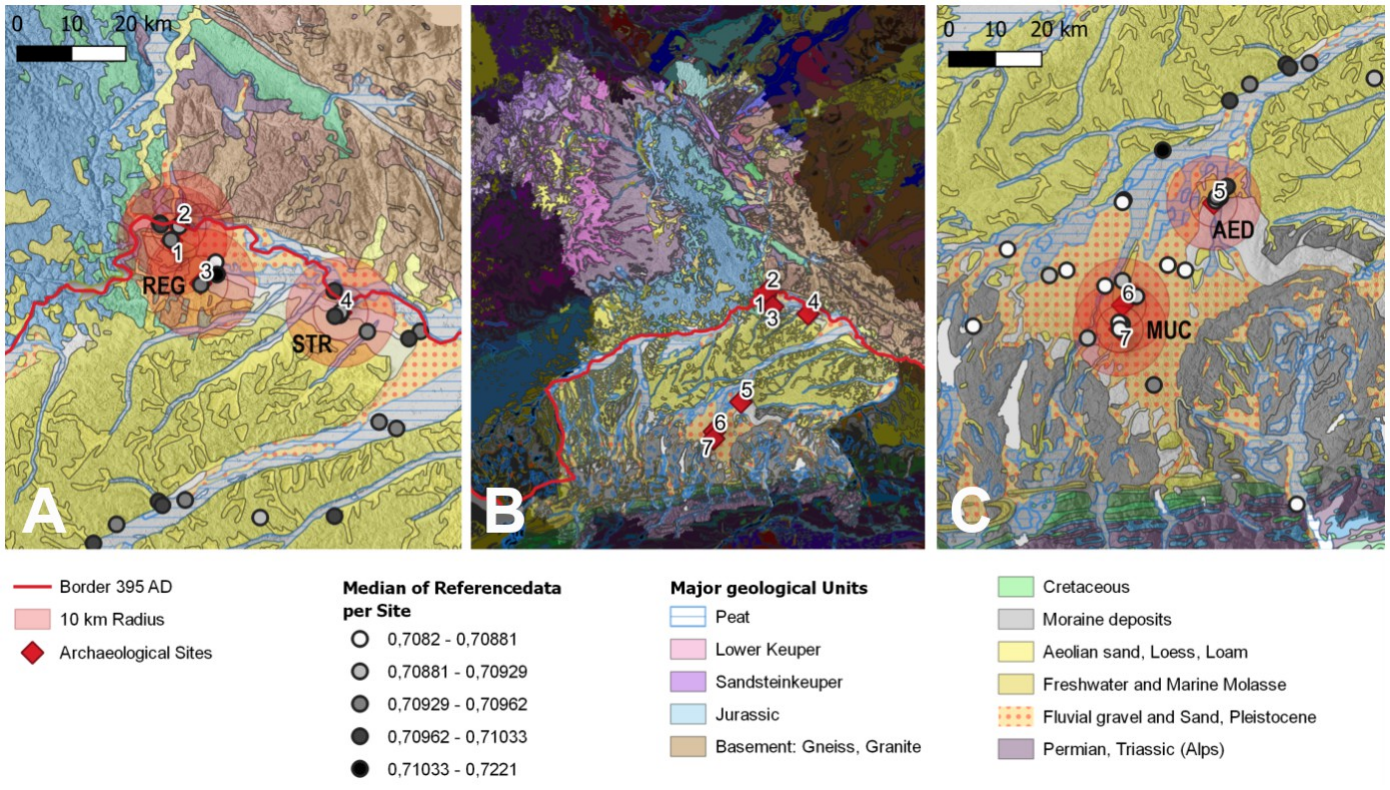
Geological maps of the study region. Only the main geological units are named (Illustration: QGIS, map base GK1000 © BGR Hannover (2014) [60]. Left (A) and Right (C) Corresponding details. Grey: Location and median of strontium values of the reference data. Red circles: 10-km radii (2x5 km) around the archaeological sites (overlapping for two sites). This resulted in four regional groups: Regensburg (REG, with Burgweinting, Irlmauth and Alteglofsheim), Straubing (STB), Erding (AED), and Munich (MUC, with Munich-Perlach and Unterhaching). Center (B) Geological map of Bavaria and locations of archaeological sites: 1 Burgweinting A and B (BWA, BWB), 2 Irlmauth (IRM), 3 Alteglofsheim (AEH), 4 Straubing-Bajuwarenstraße (STB), 5 Altenerding (AED), 6 Munich-Perlach (PEL), 7 Unterhaching (UTH). (created with QGIS 3.18.3 Zürich).

The study area in the North Alpine foreland of Bavaria has a fairly uniform, relatively young geological surface and is surrounded by regions of, at least partly, different geology e.g., with exposed older rocks ([58], Fig 2, S2.2 section in S2 Text). Based on an extensive data set of over 900 skeletal samples from various time periods, bioavailable strontium values between 0.70800 and 0.71050 are to be expected ([59] and S2.3.2 section in S2 Text).

The range of bioavailable strontium is usually defined by inferring a local isotopic range (‘baseline’) using proxies e.g., geological data, strontium values of archaeological bones and teeth from animals, modern plants and shells, or a combination of it [54, 61, 62]. Currently, there is little agreement on how to best establish a baseline and which proxies to be used (e.g., [63–66]). All approaches show some weaknesses e.g., environmental samples may be altered due to anthropogenic contaminations, limited sample size may underestimate the local bioavailable ^87^Sr/^86^Sr ratios [54, 62, 67, 68]. A number of studies draw inferences directly from human data using the distribution of human enamel data to determine the local range of ^87^Sr/^86^Sr (e.g. [69–73]). The main peak of the distribution of the data can be interpreted as the local ^87^Sr/^86^Sr ratio for humans [74], provided by some temporal depth of the data and the absence of evidence that most of the population was non-local (e.g., [75]).

Other studies use data from human [76, 77] bones. Bone, however, is more permeable compared to tooth enamel and thus strontium from groundwater can alter the Sr-values towards a “local” signal [27]. Thus, bones are less likely to show a non-local signal than teeth (see S2.3.2 section in Text for more details). However, if diagenetically incorporated strontium increasingly masks the autochthonous signal, the acutal variability of bioavailable strontium in the population investigated is shifted toward groundwater value. Hence bone values alone are not suitable for determining the range of bioavailable ^87^Sr/^86^Sr.

In this study, we combine the bone values of animals and humans with the enamel ratios of of animals and humans of different sites and time periods to determine the local baseline of a region. Data of human enamel from one of the sites (Altenerding) already proofed suitable for the determination of local isotopic variability of the population [54]. Additionally, the very largest part of strontium isotope data is within the typical range for the Northern Alpine foreland (see S2.3.2 section and S2.3.3.1 Fig in S2 Text), providing no evidence that the largest proportion of individuals are immigrants.

To identify potential small-scale differences, we established baselines for the regions surrounding the burial sites. A region was defined as area within a 10 km radius around the burial place. Neighboring sites were combined into one regional group if their 10 km radii overlapped (Fig 2). Faunal and human remains (enamel and bone) were used and strontium isotope data from other time periods was included to ensure greater temporal depth (see S2.3.3.1 Table in S2 Text).

The central tendency of the data was determined using Gaussian kernel density estimates (KDEs). The mode in KDEs most likely represents the values of the main population. Highest density intervals (HDIs) were calculated to indicate which interval covers most of the distribution and which point are most credible ([78], see also Statistical methods below). Intervals that span 99% of the distribution (99% HDIs) define the biologically available ^87^Sr/^86^Sr range of the individual region.

#### Non-locals and migrants

Individuals are defined as “non-local” if their strontium isotope ratio in tooth enamel is outside the 99% regional HDI. However, an ^87^Sr/^86^Sr ratio within the local biologically available range does not necessarily mean that the individual can be considered local. The ratio could also derive from a region with similar geology and biologically available ^87^Sr/^86^Sr signature. Due to the geographical redundancy of ^87^Sr/^86^Sr it can be assumed that a certain proportion of non-local individuals remain undetected and thus only the minimum proportion of non-locals can be indicated. Similarly, it is not possible to pinpoint a specific region of origin for detected non-locals. Yet, regions of origin can be excluded e.g., if the ^87^Sr/^86^Sr ratio of an individual exceeds the range of the alleged region of origin. If the ^87^Sr/^86^Sr ratio is within the range of an alleged region of origin, however, it might as likely derive from a region with similar conditions.

Non-locals whose ^87^Sr/^86^Sr ratios fit into the range of another region within the pre-Alpine area and thus likely moved across shorter distances within South Bavaria are referred to as “mobiles”. We only refer to individuals as “migrants” if other regions in the Northern pre-Alpine area can be excluded as their region of origin, following the concept that migration occurs over longer distances and cultural and/or political boundaries.

#### Comparing migration over time

The number of non-locals that can be reliably detected via strontium can vary depending on the geological conditions of both, the region of origin and burial site, as well as the type of data available and methods used. Thus, comparing migration rates is problematic. To minimize this issue and to estimate the extent of migration in South Bavaria over time we calculated a minimum frequency of migrants (MFM = percentage of specific immigrants) in the North Alpine area for different time periods (Late Neolithic, Early Neolithic, Early Bronze Age, Iron Age, Late Antique, around 500 AD and Early Middle Ages) based on the enamel data of adults from the literature (N = 519), using the range of the bioavailable ^87^Sr/^86^Sr calculated for the whole area (based the adjusted reference data set (N = 865) (for more detail see S2.3 section in S2 Text).

As strontium ratios from the region of Regensburg significantly exceeded the upper limit for the Northern pre-Alpine region due to their high variability (see Results) we excluded this region from calculations of MFM.

### Carbon and nitrogen isotope analysis

#### Sample preparation and analysis

Bone collagen was extracted following the protocol described in Siebke et al. [79]. Bones were cleaned in an ultrasonic bath with distilled water, air-dried, and ground into powder. 250–300 mg of powder were demineralized in 1M HCl at room temperature for 20 min, washed with distilled water, and transferred into 0.125M NaOH for 20 h at room temperature. After washing with distilled water until neutralization, they were gelatinized in 0.001M HCl at 90°C for 10–15 h. Solubilized collagen was filtered and lyophilized.

For serial incremental dentine sampling molars were bisected longitudinally. Samples were demineralized in 0.5M HCl at 4°C and then washed with distilled water until pH-neutral. The tooth halves were cut into 1-mm thick slices with a scalpel (see S2.2.1 section S2 Text). Dentine sections were incubated in 0.125M NaOH for 24 h at room temperature, and rinsed with distilled water until neutral pH and then gelatinized in 0.01M HCl for 17 h at 80°C [80]. Liquid fractions were frozen and lyophilized. 0.3–0.8 mg of bone or dentine collagen was transferred into tin capsules for mass spectrometry.

Samples were analyzed in singles at the GeoCenter (Friedrich-Alexander University, Erlangen-Nürnberg) with a Flash EA 2000 elemental analyzer connected to a ThermoFinnigan Delta V Plus mass spectrometer. Some measurements were carried out at the Isolab GmbH in Hanau using an Elementar Vario Cube EL connected to an Isoprime mass spectrometer (see S1.1 Table in S1 Text for detail). Results are reported in the conventional δ-notation in permil (‰) relative to internationally accepted standards, VPDB for carbon and AIR for nitrogen. Accuracy and precision were checked by replicate analyses of laboratory standards (e.g., Isolab: Collagen STD R (USGS 89), Collagen STD S, Collagen STD BRA; GeoCenter: Casein, Cyclo) calibrated to international standards USGS40 and 41. Analytical precision ensured by the GeoCenter is 0.1‰ for δ^13^C and δ^15^N as well as 0.1‰ for δ^13^C and 0.2‰ for δ^15^N by the Isolab GmbH. Comparing data from different laboratories results in an expanded uncertainty which is generally specified as twofold analytical precision. The collagen quality of samples was evaluated through the C/N atomic ratio (between 2.9 and 3.6 [81]) as well as carbon (in excess of 13%) and nitrogen content (in excess of 4.8%) to indicate sufficient collagen quality [81–84]. Samples were excluded if they had elevated carbon (>50%) and/or nitrogen (>19%) contents, indicative of contamination.

Approximate ages were assigned to dentine sections using a customized scheme based on *The London Atlas of Tooth Development and Eruption* [47]. For detailed description of dentine sampling and age assignment of samples see S2.2.2 section in S2 Text.

#### Determining basic human diet

To characterize the main components of the diet in Bavaria we used human δ^13^C and δ^15^N bone collagen data of adults from all investigated sites in comparison to faunal samples (see S3.1 section in S3 Text for details). We take inter- and intra-populational variability of δ^13^C and δ^15^N values into account, which can be influenced by various factors: Isotope signals in bone represent a mixture of dietary intake over a longer time due to tissue turnover (see above) and nutritional stress can influence δ^13^C and δ^15^N values [85–87]. Also, divergent natural conditions in the different areas that directly influence the isotopic ratios in plants (e.g., [88–90]). Inside natural limits, different subsistence strategies such as different practices in agriculture and husbandry, fishing and hunting as well as trading food, access to resources (possibly linked to sex or age), social position etc. (e.g., [91–93]) can result in intra-populational differences. However, variability in diet can also be linked to the mixed composition of communities including individuals of different origins and ethnicities [94].

#### Determining the “common intra-populational diet variability”

The study region is spatially rather narrow and has fairly uniform ecogeographic conditions and thus is likely inhabited by the same type of plants and animals. Human data therefore generally cluster in a range that is limited by the characteristics of the ecosystem and food resources. However, small inter-populational differences are possible.

Therefore, we subdivided population groups by their location. Data from neighboring sites (<5 km) like Munich-Perlach and Unterhaching, as well as Burgweinting, Irlmauth, and Alteglofsheim were combined. These sites have similar external conditions e.g., ground conditions (Munich gravel plain or river valleys covered with loess soils) and/or overlapping catchment areas for crops and animals [95, 96]. The scattering of δ^13^C and δ^15^N values of adults of combined sites supports this approach. Additionally, the sample size is increased, reducing the risk that the variability is underestimated.

To describe intra-populational variability we use KDEs and HDIs. The 99% intervals cover the variability determined by both external and internal factors and thus are suitable to define the “common variability” of a population’s diet.

#### Detecting dietary outliers

Individuals who show δ^13^C and/or δ^15^N values outside the 99% HDIs of the respective population are defined as outliers. The diet of these individuals was unusual for the region of their burial site, either because of food imports or because they moved there from a region with different diet [33, 34]. Similar to strontium isotope analysis, it is impossible to pinpoint the region where non-local diet or individuals originally came from. A specific kind of ecosystem can be suspected at the very most. It is also likely that people move from one land-locked region to another within the same ecosystem in temperate Europe without significantly changing their diet [22].

Different diet during early life is likely no longer evident in bones of an older individual because of the turnover of bone collagen. However, it can be detected through the analysis of tooth dentine.

#### Comparing childhood and adult diet

We investigated inter- and intra-individual variability aiming to detect shifts in isotope ratios possibly be linked to a change in residency. Bone samples from adults show dietary pattern in a later life stage. To analyze diet in childhood we used collagen data from dentine of the first molar. Serial dentine samples provide high-resolution biographical data pointing out dietary changes during an individual’s life (e.g., [97]). Fluctuations in micro-sample profiles can be linked physiological stress and migration [98]. Bulk dentine represents the mean values of root dentine sections from the serial analysis.

First, we compared δ^13^C and δ^15^N values of bulk dentine and bone samples from every individual and identified outliers from the predefined “common variability” of a population’s diet in the bulk dentine dataset. Then we tested the hypothesis whether these outliers also show deviating ^87^Sr/^86^Sr in enamel, deviating δ^13^C or δ^15^N in bone or the presence of ACD as all these features can indicate foreign origin. Individuals with evidence of foreign origin are also likely to show deviating stable light isotopic ratios in tooth dentine due to their potential stay in a different ecosystem. However, individuals that changed residency during early childhood can only be detected using sequential dentine analysis.

### Statistical methods

#### KDEs and HDIs

Gaussian KDEs and corresponding HDIs of ^87^Sr/^86^Sr, δ^15^N, and δ^13^C data groups were calculated with RStudio 1.4.1717 for Windows using hdr.den function of package hdrcde [99]. The optimal bandwidth was selected using the "solve-the-equation" method of Sheather & Jones [100] based on the adjusted reference sample set for ^87^Sr/^86^Sr (S2.3.2 Text) and combined sample sets of all sites for δ^13^C and δ^15^N (S2.4 Text). We calculated 99%, 95% and 90% HDIs below the most prominent mode in KDEs using Hyndman’s density quantile algorithm [99] but used the 99% HDIs to determine cutoffs most conservatively and minimize the risk of overestimating the number of individuals with deviating ratios due to methodological uncertainties (see S2.3.3 and S2.4 section in S2 Text for more detail).

#### Statistical tests

Non-parametric statistical tests like Mann-Whitney U test [101], Kruskal-Wallis test [102], and Dunn’s post hoc test [103] were primarily conducted on metric data due to small sample sizes and the properties of the data (e.g., no normal distribution). Parametric tests like t-test [104], ANOVA [105], Hochberg post hoc test (for equal variances, [106, 107]), and Dunnett T3 (for unequal variances, [108]) were used when appropriate (e.g., normal distribution). Nominal data were analyzed by non-parametric Chi-square tests [109]. Significance values for multiple comparisons were adjusted by the Bonferroni correction [110] to counteract alpha error accumulation. Calculations were made with SPSS 26.00 for Windows. Interpretations are based on explanations given by Wilcox [111].

## Results

### Strontium isotope analysis

#### Local ranges and outliers

The strontium ratios of the human enamel samples in our study had a mean of 0.71023±0.00149 (range: 0.70658 to 0.71481).

**Fig 3:**
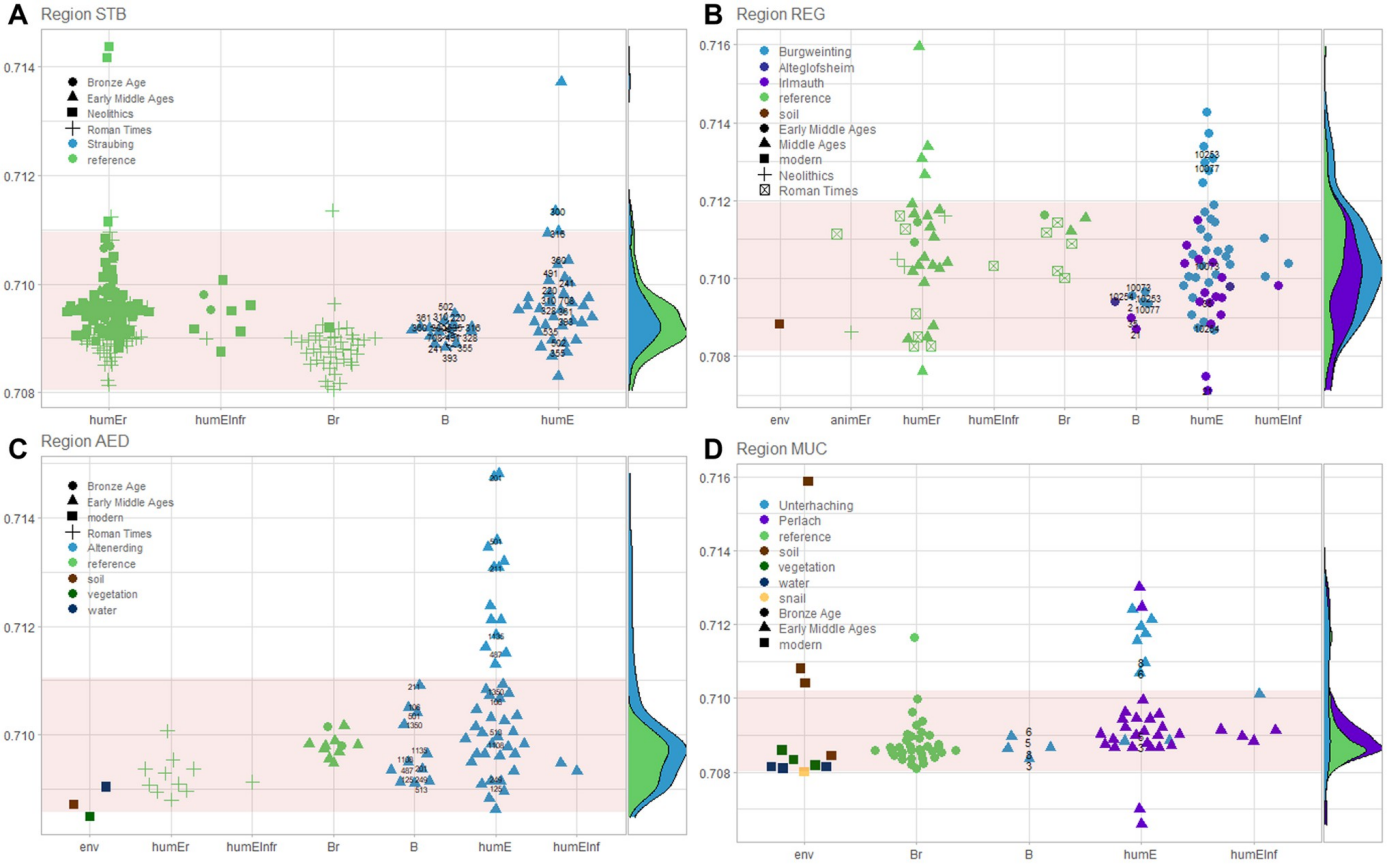
Local ranges and distribution of ^87^Sr/^86^Sr values per region and site. (A) Region Straubing (STB), (B) Region Regensburg (REG), (C) Region Erding (AED) and (D) Region Munich (MUC). ^87^Sr/^86^Sr (y-axis) of samples from predefined study region divided into groups (x-axis): env = environmental samples, animE = animal enamel, humE = human enamel, humEInf = human enamel of infans (0–12 years), B = bone (animal or human) (followed by an r = part of the reference data set, no r = archaeological site investigated). Red area: calculated range of bioavailable strontium based on all biomineral samples of the region. Symbols provide a chronological classification. Numbers = grave numbers of individuals from which both bone and enamel were measured. If values from environmental samples were known from the literature, they were added for comparative reasons: Lengfelder et al. [112]: soil, vegetation, water for AED and MUC, Hoogewerff et al. [113]: soil for REG, Neumann et al. [114]: soil and snail MUC. Right side: Distribution of samples separated by reference and respective site: Straubing (STB): N = 217, Regensburg (REG): N = 80, Erding (AED): N = 78, and Munich (MUC): N = 76 (illustration: R with packages ggplot2 [115, 116], ggbeeswarm [117], ggside [118], ggrepel [119]).

For the entire pre-Alpine region south of the Danube (N = 865) a local range of 0.7081–0.7110 could be determined (S.2.3.2 Text). The following ranges were calculated for smaller-scale regions (S.2.3.3 Text): Straubing (STB): 0.70805–0.71096, Regensburg (REG): 0.70813–0.71195, Erding (AED): 0.70857–0.71104, and Munich (MUC): 0.70802–0.71021. In Fig 3, calculated ranges are shown in red and relevant values are presented in groups.

Regional strontium ranges showed some shifted limits that partially exceeded the range of bioavailable strontium determined for the entire pre-Alpine region south of the Danube. This is especially true for the span of Regensburg. Compared to the Straubing, Erding, and Munich sites, which showed distinct main peaks at similar values, Regensburg had a broader and multi peaked distribution shifted toward higher ^87^Sr/^86^Sr values (also see S2.3.2 section in S2 Text).

As expected, in all regions human and animal bone values, as well as enamel values of children (which most likely not relocated) show a narrower range than values of dental enamel of adults (see S2.3.2 section in S2 Text and Methods). For some individuals, ^87^Sr/^86^Sr was measured in both bone and enamel (indicated by numbers in Fig 3A–3D). In all these cases bone ^87^Sr/^86^Sr values show a signal within the local range, even if the corresponding enamel values are far outside this range.

The range determined from the total set of regional biomineral samples (red area) exceeds in each case the variation of environmental samples. Except some soil samples (region MUC, Fig 3D) which show implausible high values and let suspect methodological problems (as discussed in Toncala et al. [54]). The range of bioavailable strontium based on the total biomineral of the region also exceeds those ranges covered by materials usually taken as reference for local values (tooth enamel of children and animals, bone values). Thus, we consider the chosen method for estimating the local range not only as reliable but also suitable to not overestimate potential newcomers.

For a total of 36 individuals, an origin outside a 10 km radius of their burial site (regional range) can be assumed on the basis of their strontium values in tooth enamel (Table 2). Of these, the ^87^Sr/^86^Sr values of nine individuals from three sites exceeded the local ranges but fall into the local ranges of another region in the Northern Alpine foreland of Bavaria (see Table 2). For the majority of these individuals (N = 7) the region around Regensburg with its higher ^87^Sr/^86^Sr range can be considered.

**Table 2:** Characteristics of assumed newcomers according to deviating isotopic ratios or presence of ACD.

Individual (N = 50)	Stable isotopes					
	^87^Sr/^86^Sr enamel	^87^Sr/^86^Sr bone	δ^13^C [‰] bulk bone	δ^15^N [‰] bulk bone	δ^13^C [‰] bulk dentine	δ^15^N [‰] bulk dentine
STB_228* ♀,m	-	0.70883	-18.7	9.0	-	-
STB_300 ♀,m	*0*.*71135*	0.70921	-19.9	8.8	-18.9	9.5
STB_328* ♀,m	0.70960	-	-19.6	8.8	-	-
STB_361* ♀,m	0.70953	0.70921	-19.6	9.2	-15.3	10.0
STB_395 ♂,s	0.71372	-	-20.0	10.2	-	-
STB_535* ♀,m	0.70923	0.70919	-20.1	9.1	-16.0	9.2
AED_94 ♀,m	0.71211	-	-20.0	8.7	-	-
AED_125* ♀,m	0.70908	0.70913	-19.3	9.3	-16.8	10.3
AED_160 ♀,s	0.71481	-	-20.4	9.6	-	-
AED_201 ♀,m	0.71475	0.70937	-20.0	9.0		
AED_211 ♀,m	0.71308	0.71090	-19.3	9.3	-19.4	11.7
AED_321 ♂,m	0.71238	-	-19.9	8.8		
AED_343 ♀,m	0.71345	-	-19.1	7.9	-17.3	9.6
AED_344 ♂,m	*0*.*71130*	-	-19.3	9.6	-19.7	9.7
AED_421 ♀,s	0.71092	-	-20.0	11.2		
AED_487 ♂,m	*0*.*71150*	0.70935	-19.5	9.7		
AED_492 ♂,m	*0*.*71161*	-	-19.5	8.9	-19.8	9.6
AED_501 ♂,m	0.71358	0.71041	-19.4	9.9	-19.8	9.8
AED_513* ♀,m	0.71006	0.70910	-16.8	9.1	-15.9	11.2
AED_521 ♀,m	0.71320	-	-18.8	9.8	-	-
AED_1108* ♀,s	0.70982	0.70950	-18.2	10.4	-	-
AED_1123 ♂,s	0.71212	-	-19.8	9.6	-	-
AED_1135 ♀,a	*0*.*71183*	0.70965	-20.3	10.1	-	-
AED_1143 ♂,a	0.71309	-	-19.7	10.0	-19.6	9.0
AED_1350* ♀,m-s	0.71076	0.71019	-18.9	9.6	-	-
IRM_20 ♀,j	0.70750	-	-20.0	8.9	-	-
IRM_21 ♀,m	0.70714	0.70870	-19.9	9.0	-	-
IRM_33* ♀,m	-	-	-19.8	9.5	-	-
BWB_3734 ♂,a	0.71247	-	-21.0	9.2	-	-
BWB_3735 ♀,m	0.71426	-	-20.2	9.5	-	-
BWB_3739 ♀,m	0.71340	-	-19.8	8.7	-	-
BWB_3740 ♀,a	0.71373	-	-20.7	9.2	-	-
BWB_3741 ♀,m	0.71278	-	-20.9	9.0	-	-
BWA_10071 ♀,a	0.71076	-	-15.0	7.7	-14.2	8.4
BWA_10077 ♀,a	0.71297	0.70936	-20.2	9.7	-	-
BWA_10253 ♂,m	0.71309	0.70941	-19.7	9.3	-	-
BWA_10254* ♀,s	0.70873	0.70956	-19.5	10.0	-16.8	11.9
AEH_145* ♀,m	0.70979	0.70941	-19.4	10.0	-	-
PEL_12 ♀,a	0.70658	-	-19.1	8.2	-18.2	9.9
PEL_18 ♀,s	0.70866	-	-18.1	8.9	-	-
PEL_19 ♀?,i II	0.70699	-	-19.1	8.2	-	-
PEL_22 ♂,a-m	0.71247	-	-19.7	9.0	-	-
PEL_27♂,m	0.71300	-	-19.2	9.0	-	-
UTH_1 ♀,m	*0*.*71156*	-	-19.2	9.3	-	-
UTH_2 ♀,m	0.71214	-	-	-	-	-
UTH_6 ♂,m-s	*0*.*71068*	0.70897	-19.5	9.0	-	-
UTH_7 ♂,m	*0*.*71175*	-	-19.7	9.3	-	-
UTH_8 ♀,m	*0*.*71096*	0.70863	-	-	-	-
UTH_9 ♀,a-m	0.71195	-	-19.2	8.7	-	-
UTH_10 ♀,a	0.71241	-	-	-	-	-

Skull shape: * = modified (ACD) highlighted in bold, no information = not modified. Sex: ♀ = female, ♀? = rather female, ♂ = male. Age at death: i-II = infans II, j = juvenile, a = adult, a-m = adult-mature, m = mature, m-s = mature-senile, s = senile. Isotope data that identify individuals as noticeable are highlighted by grey background. Italic ^87^Sr/^86^Sr enamel: Although these Individuals were identified as not being local to their burial site, an origin from another site of the North Alpine Bavarian foreland cannot be excluded.

Overall, the majority of outliers (N = 32) referred to regions with higher strontium signatures than in the Northern Alpine foothills, such as geologically older regions with granite bedrock in the Bavarian Forest or Bohemia. Only four isotope values were below the local range of our study region. This indicates an origin from regions partially shaped by younger rocks, such as volcanic stone, or those dominated by chalk/dolomite/limestone or impure carbonate sedimentary rocks (e.g., [64]), such as found in South Tirol.

#### Non-locals and migrants

**Fig 4:**
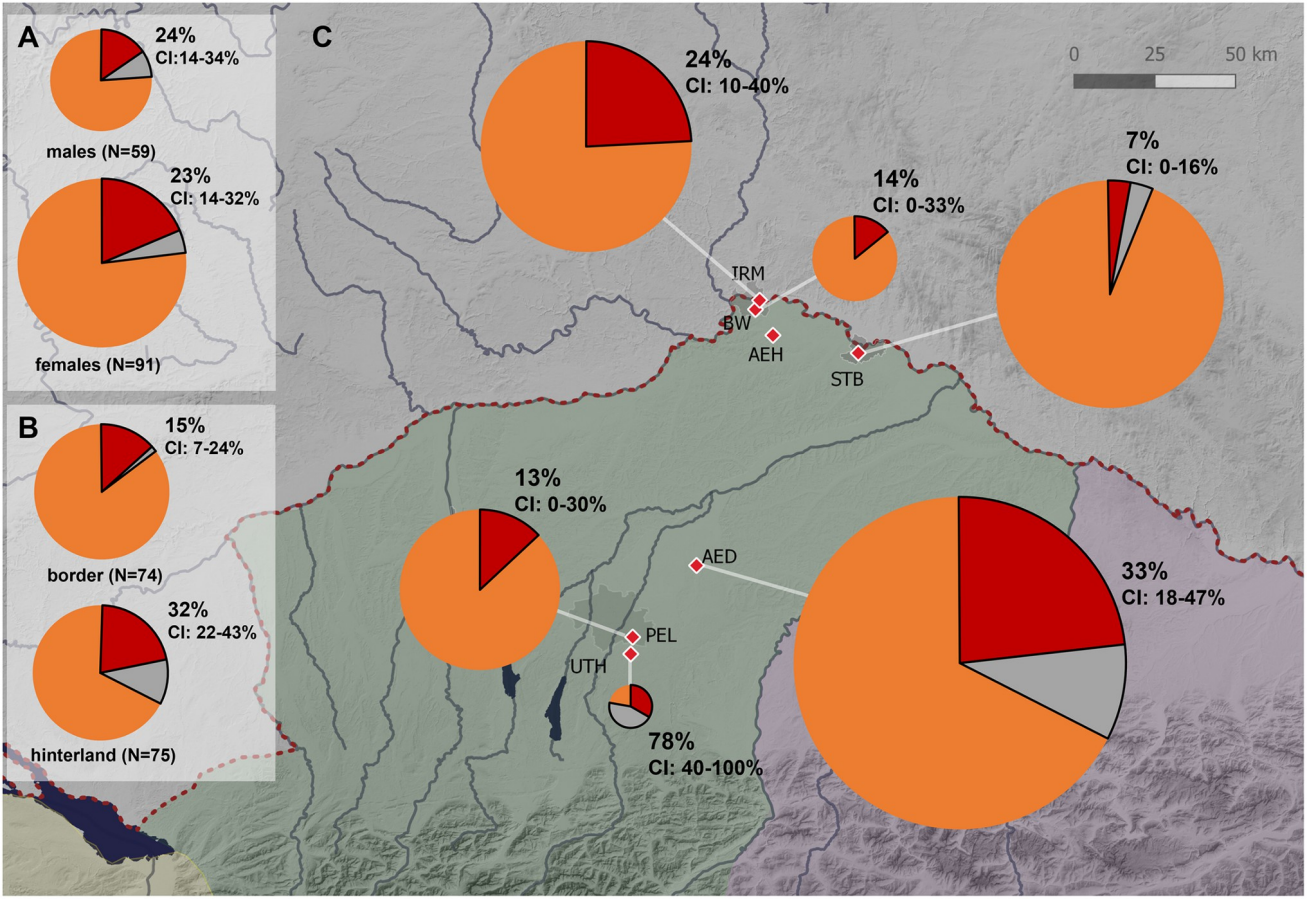
Proportion of non-locals. Circular charts illustrating the proportion of non-locals (A) in gender groups (females vs. males), (B) in settlement zones (border vs. hinterland), (C) for archaeological sites (VW Burgweinting, IRM Irlmauth, STB Straubing-Bajuwarenstraße, AED Altenerding, PEL Munich-Perlach, UTH Unterhaching). Given numbers are frequencies of all non-locals, whose strontium values are found outside the range of their burial region, which summarize the frequencies of individuals who show strontium ratios outside the local range of the Northern Alpine area in South Bavaria (filled in dark red) and individuals whose strontium values are found in other study regions in South Bavaria (filled in grey). (Map data: see Fig 1). (created with QGIS 3.18.3 Zürich and Excel).

In total 23% (95% CI: 17–30%) of all individuals are of non-local origin with an equal number of non-local females (23%, 95% CI: 14–32%) and males (24%, 95% CI 14–34%) (Fig 4A, S3.4.1.2 Table in S3 Text). Of the nine mobile individuals whose strontium values are not in the local range of their burial region, but who show values within the Northern Alpine region (especially Regensburg), no differences between the sexes can be detected (5 men and 4 women, see Table 2). If these nine individuals are excluded, in the group of individuals who show strontium ratios from geologically older or younger regions than South Bavaria females (19%, 95% CI: 11–28%) and males (15%, 95% CI: 7–25%) are still equally represented (S3.4.1.4 Table in S3 Text). Further characteristics (e.g., age at death etc.) for identified non-locals are given in Table 2. Notably, no woman with ACD has a strontium isotope ratio that falls outside the range of local bioavailable strontium of the respective regions or the Northern Alpine region.

#### Spatial pattern

A significant difference (Chi-Square Test: p = 0.014) between the predefined settlement zones was apparent; specifically, the frequency of non-locals in the border area (15%, 95% CI: 7–24%) was lower than in the hinterland (32%, 95% CI: 22–43%) (Fig 4B, S3.4.2.2 Table in S3 Text). In addition, some local differences (Chi-Square Test: p<0.001) in the proportion of non-locals between archaeological sites were observed (Fig 4C, S3.4.3.2 Table in S3 Text). Pairwise comparisons (S3.4.3.1 Table in S3 Text) of sites revealed a significantly higher number of non-locals in Unterhaching (UTH) than expected (Post Hoc Chi-Square Test: p<0.001). These differences are also visible if the frequency was calculated only for migrants, however not significant (S3.4.2.4, S3.4.3.4 Table in S3 Text).

#### MFM rates over time

**Fig 5:**
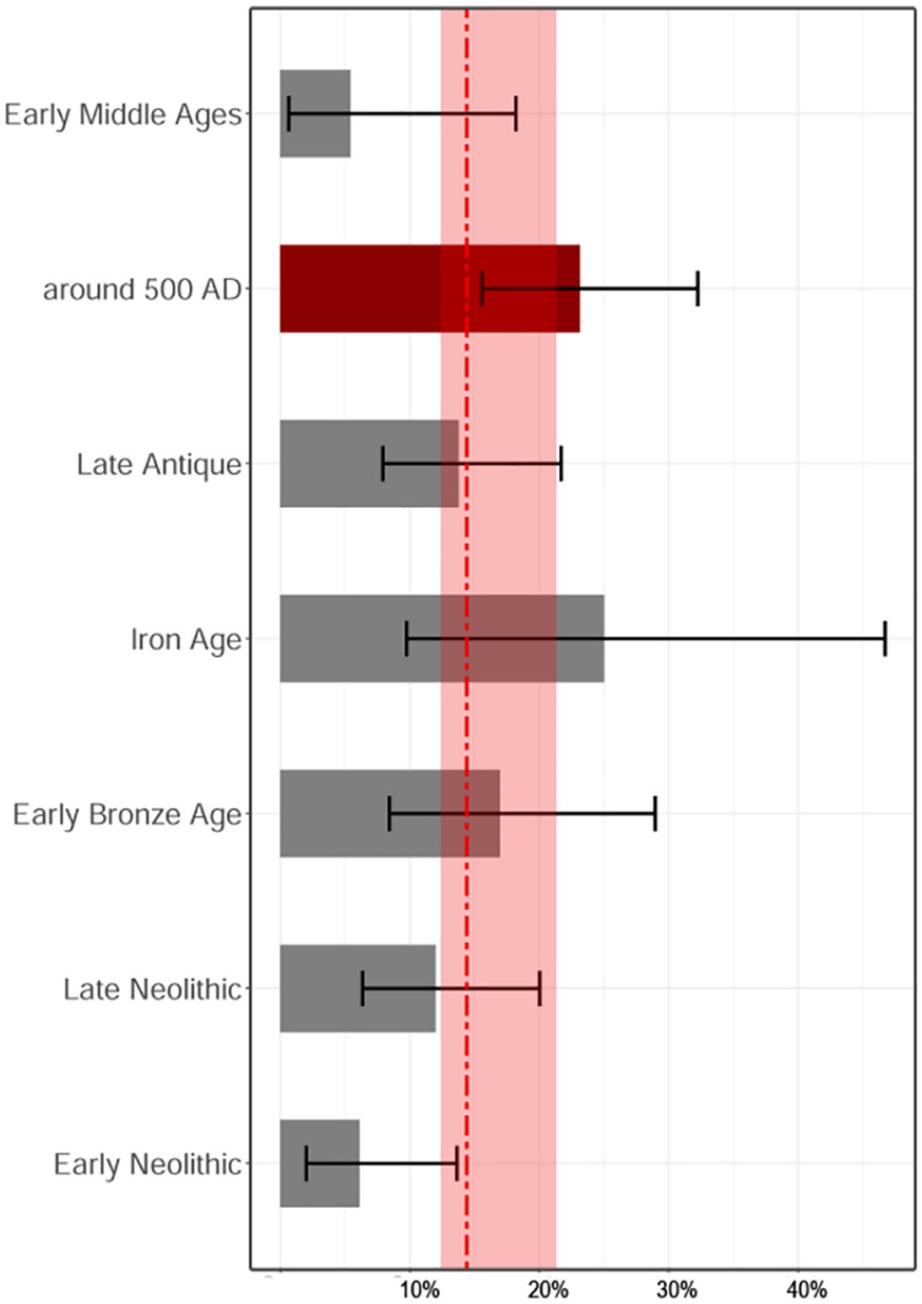
Minimum Frequency of Migrants (MFM) in different periods. Late Neolithic N = 108, Early Neolithic N = 82, Early Bronze Age N = 59, Iron Age N = 24, Late Antique N = 109, around 500 AD N = 108 and Early Middle Ages N = 37. Error bar = confidence interval, red line = mean frequency of immigrants if all periods are considered together, red box = confidence interval for this average.

The average frequency of MFM across all periods was around 15% (95% CI:14–22%), but we observed chronological variations (Fig 5, Chi-Square Test: p = 0.011). The MFM calculated for the cemeteries around 500 AD amounted to 23% (95% CI:15–31%), which was higher than the average, and also higher than the proportions in most other periods (Post Hoc Chi-Square Test: p = 0.0039, S3.5.2 Table in S3 Text).

### Carbon and nitrogen analysis

#### Bone collagen: Variability of diet and outliers

The human bone δ^15^N values clustered around a mean of 9.2±0.7‰, ranging from 7.4‰ to 11.2‰. The δ^13^C values had a mean of -19.7±0.7‰, with an overall range of -21.0‰ to -15.0‰. The scattering of stable light isotopes suggests that individuals consumed different proportions of plant and animal food sources. We found no significant differences between the δ^13^C or δ^15^N values of men and women (S3.1.1.1.1 Table in S3 Text), but females showed a higher variance in δ^13^C values (S3.1.1.1.2 Table in S3 Text).

**Fig 6:**
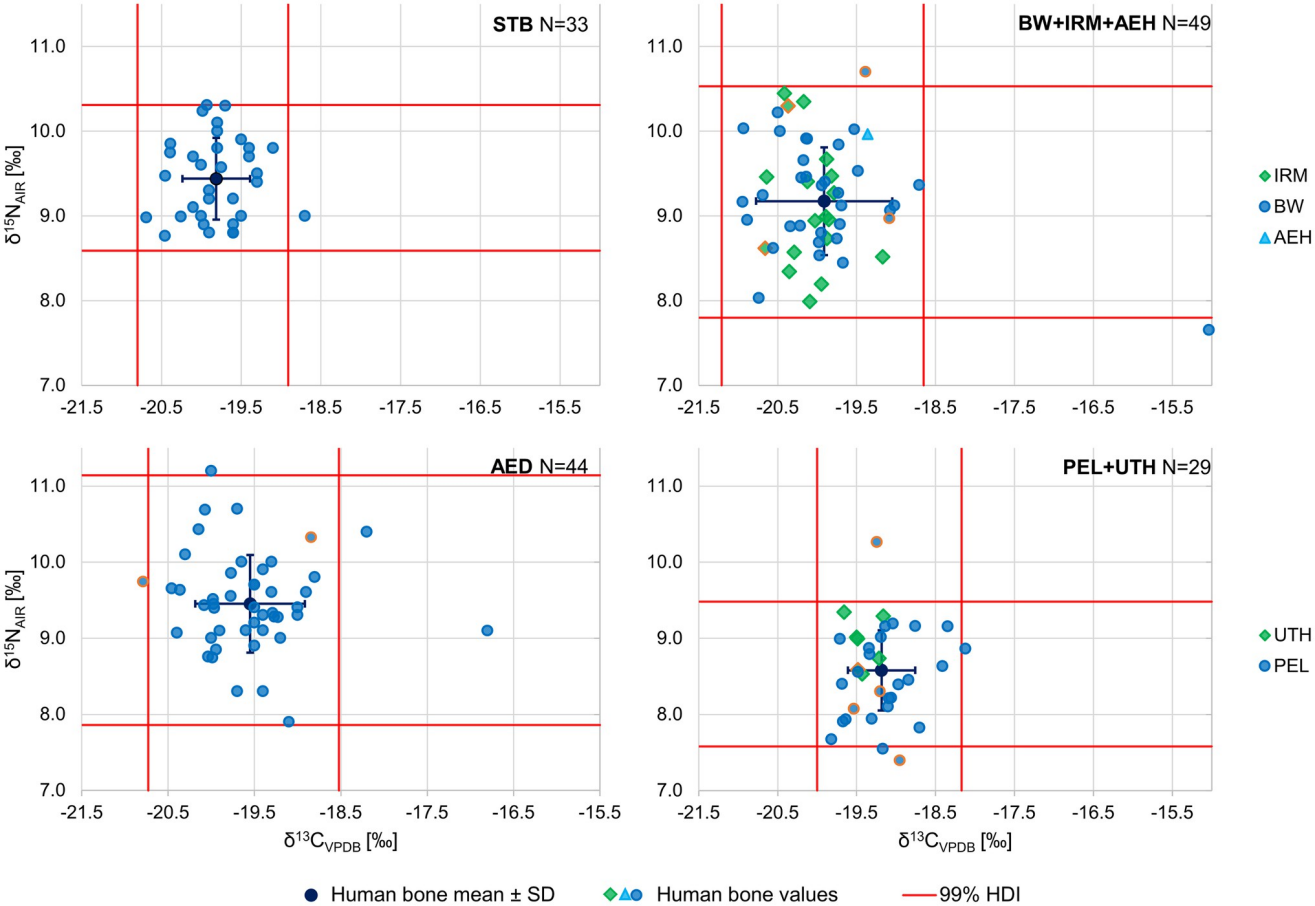
Variability of carbon and nitrogen isotopic ratios. Scatter plots of δ^13^C and δ^15^N values of human bone collagen: Straubing-Bajuwarenstraße (STB, N = 33), Burgweinting, Irlmauth and Alteglofsheim (BW+IRM+AEH, N = 49) in the border zone, and Altenerding (AED, N = 44), Munich-Perlach and Unterhaching (PEL+UTH, N = 29) in the hinterland. Mean values and standard deviations (SD) are based on postweaning individuals (excluding infans I children = values outlined in orange) and were calculated using SPSS. Red lines represent 99% HDIs (calculations: hdr.den function of R package hdrcde [99]) (illustration: Excel).

While the human isotope ratio values from human bone collagen generally overlapped (Fig 6), we also observed some site-specific variation (Kruskal-Wallis Test: p<0.001 for both δ^13^C and δ^15^N values). Samples from Munich-Perlach were shifted towards decreased δ^15^N values and increased δ^13^C values compared to most other sites (S3.1.1.4.2 Table in S3 Text). Moreover, Burgweinting, Irlmauth, and Altenerding showed more variable δ^13^C and δ^15^N values compared to those from Straubing-Bajuwarenstraße, Munich-Perlach, and Unterhaching. Six individuals, solely females, had isotopic values outside the common variability of the population (Table 2). Five outliers had increased δ^13^C bone values, but two δ^15^N bone values below or above the “common variability” were also found.

#### Bulk dentine collagen: Childhood diet

**Fig 7:**
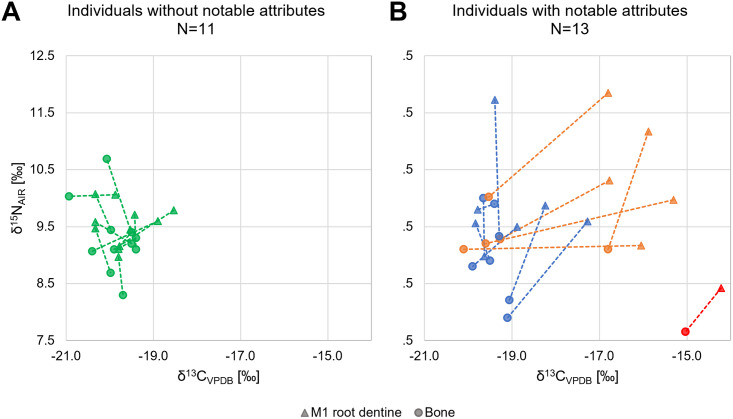
Differences between bulk dentine and bone collagen. (A) Differences between bulk dentine and bone collagen of individuals without notable attributes (N = 11, illustrated in green), and (B) individuals with notable attributes (outliers in enamel ^87^Sr/^86^Sr illustrated in blue, outliers in bone collagen δ^13^C and/or δ^15^N illustrated in red, or the presence of ACD illustrated in orange) (illustration: Excel).

The mean differences between the bone and dentine collagen of individuals without notable attributes (Fig 7A) were 0.7‰ for δ^13^C and 0.5‰ for δ^15^N, reflecting normal dietary variation over a person’s lifetime. The mean differences for individuals who show notable attributes (isotopic outliers or presence of ACD) (Fig 7B), by contrast, were more than twice as large (1.5‰ for δ^13^C and 1.1‰ for δ^15^N), which is significant for δ^15^N (Mann-Whitney-U-Test: p = 0.001, S3.2.1 Table in S3 Text). Dentine δ^13^C values of nine individuals, again solely females, exceeded the “common intra-populational variability” (Table 2). Most of these outliers had increased carbon ratios, but some also showed increased δ^15^N values.

Only some migrants (determined by Strontium isotope analysis) showed a deviating dietary pattern in childhood. However, uncommon dietary pattern in childhood and the presence of notable attributes in individuals was significantly correlated (Chi-Square Test: p = 0.011, S3.2.3 Table in S3 Text). Interestingly, deviating dietary patterns from childhood were not observable in the bone collagen of these individuals, except for in two females (AED_513*, BWA_10071).

#### Serial dentine collagen: Isotope profiles

**Fig 8:**
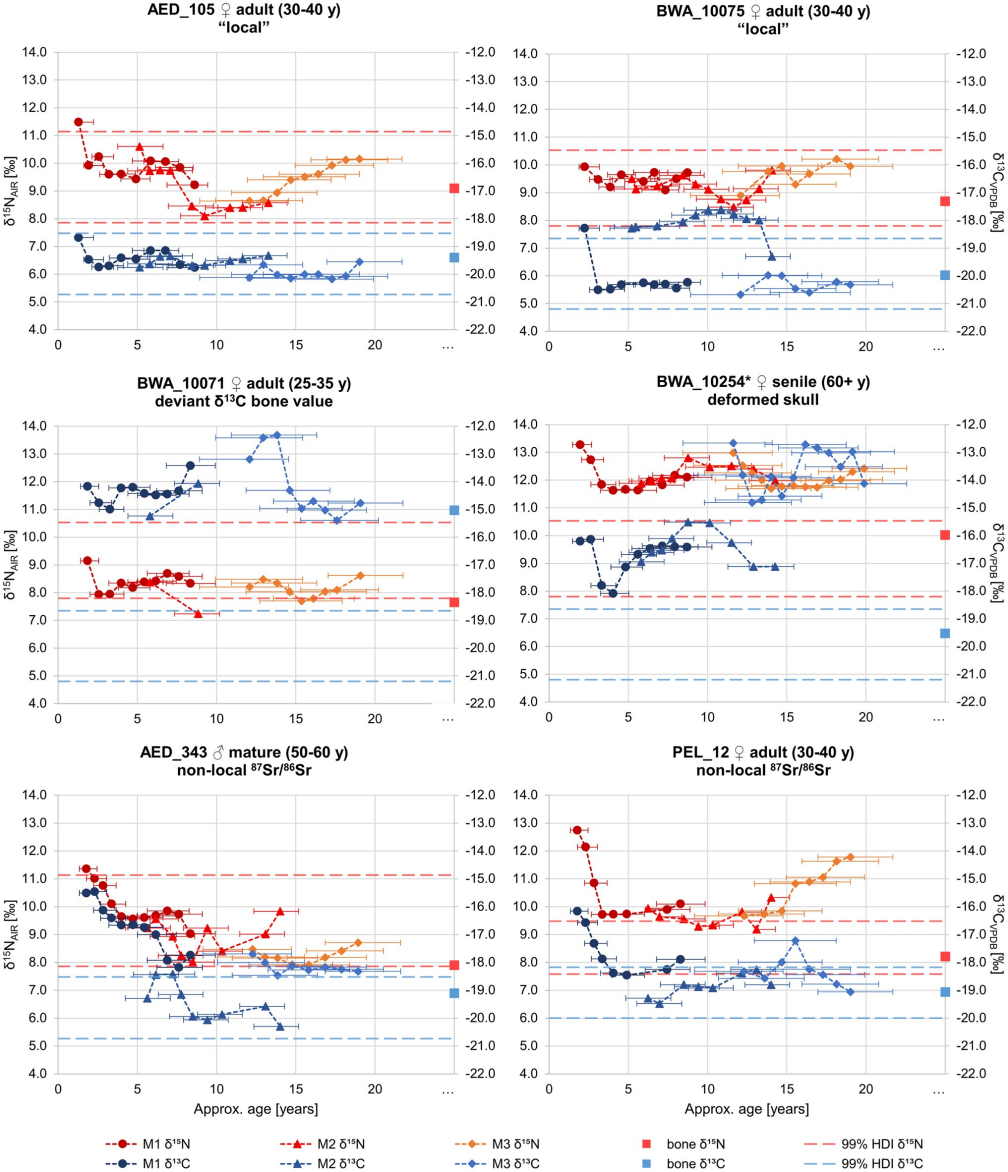
Stable carbon and nitrogen isotope profiles of six selected females (AED_105, AED_343, BWA_10071, BWA_10075, BWA_10254*, PEL_12). δ^13^C (blue shades) and δ^15^N (red shades) values of collagen from incremental dentine serial sections of molars (M1-M3) and bone collagen (square). Error bars represent the putative timespan each datapoint covers. Dotted lines represent common dietary signals at burial sites (99% HDIs) (illustration: Excel).

Selected stable C and N isotope profiles from incremental dentine serial samples of six individuals are shown in Fig 8. All profiles displayed normal variation in diet such as the decline in both isotopes at the beginning due to weaning or phases with rather low fluctuations (see S3.3 section in S3 Text for more detail). AED_105, previously determined to be “local,” showed the most stable profile, lacking any profound dietary changes in stable light isotope values within the local range. The isotopic dentine values of all predefined migrants were outside local ranges over longer periods, and their profiles included remarkable shifts in stable light isotopes outside of normal variability, especially in δ^13^C.

In the profiles of BWA_10071 and BWA_10254*, there was a shift in δ^13^C indicating a change in diet around the age of 15 years. Whereas BWA_10071 reached isotope values that corresponded to those in her burial environment, this was not the case for BWA_10254*. Here, the value only approached the burial environment in the bone, indicating that a change of diet must have occurred between the age of 20 and her death at an advanced age. The same observation was made for two other women (PEL_12 and AED_343). BWA_10075 showed a temporary shift in δ^13^C that slightly exceeded the common intra-populational variability.

## Discussion

### Diet

#### Average diet (bone collagen)

Overall, the human isotope ratio data from Early Medieval Bavaria reflected a mixed diet in a temperate C3 plant-based ecosystem. Archaeological evidence for Southern Germany from Late Antique and Early Medieval times shows that a wide range of C3 plants (cereal crops, oil and fiber plants, pulses) were cultivated [120–123]. The amount of animal protein in the diet was generally moderate and may also have been derived from low amounts of freshwater fish in addition to terrestrial animals (for more detail see S3.1 section in S3 Text).

In general, isotopic human bone collagen data from different sites show a large overlap as expected for nearby sites with rather similar ecogeographic conditions (S3.1 section in S3 Text). A notable exception is Munich-Perlach with lower δ^15^N values and higher δ^13^C values in human bone. Dietary features e.g., lower amounts of animal-derived foodstuffs and higher contents of plants, or plant parts with increased carbon ratios and/or lower amounts of freshwater fish in the diet, or different practices in agriculture e.g., land use could have been less intensive without or even with less manuring [91] (S3.1 Text).

The common assumption that men consume more animal protein or higher trophic level protein than women (e.g., [124]) is not supported by our data in general, but δ^15^N and δ^13^C mean values of males are slightly increased compared female ratios at most burial sites, except for the small necropolis in Burgweinting (BW) and Unterhaching (UTH) (S3.1.1.1.4 Table in S3 Text). The higher variability of δ^13^C values in females suggests a more variable diet, especially regarding plant resources. These findings correspond with the results of Hakenbeck et al. [22].

#### Diet in subadult age

More than half (67%) of the individuals show only slight deviations (up to 1.5‰ in δ^13^C and/or δ^15^N) between later childhood (3.5 to 9.5 years) and adulthood. This can be explained by a varying protein intake and/or varying intake of photosynthetically inactive parts of C3 plants such as roots, seeds and fruits [125] or cereal grains [126] between childhood and adulthood.

Individuals that show greater differences (more than 1.5‰ in δ^13^C and/or δ^15^N) in nutrition between childhood and adulthood also show non-local strontium isotope signals or ACD. Moreover, childhood dietary signals outside the common intra-populational variability significantly correlate with the presence of a notable attribute (ACD, deviant diet in adulthood, non-local ^87^Sr/^86^Sr) which indicate foreign origin (Chi-Square Test: p = 0.011).

A finer resolution of childhood and adolescence diets through serial dentine sampling of six women show high intra-individual variability within the life phases, in particularly in δ^13^C values. Previous studies only found higher intra-individual variability in δ^15^N [94, 127], making general growth and developmental processes unlikely to be the cause for the high variability of δ^13^C values observed in our study. Hence, we believe that strongly divergent diets in childhood and adulthood are rather caused by a change in the ecosystem than by physiological processes or culturally determined differences in the dietary habits of children and adults.

Major changes in δ^13^C appear largely around the age of 13 to 15, an age at which a social transition from childhood to adulthood can be assumed for women [128, 129]. However, the changes are not uniform, they move in different directions in different individuals. This makes traditional, local or culture-related behavioral patterns of eating as causes of these fluctuations less likely and again rather points towards individual movements between areas with different ecosystems and food bases (for detailed discussion of each individual, see S3.3 section in S3 Text).

#### Interpreting deviant diet

Significantly increased (>2‰) δ^13^C values, as detected in some individuals are usually interpreted as an indicator for the consumption of C4 plants, such as millet [130, 131]. In Late Antiquity and the Early Middle Ages millet was not unknown in Central Europe [132–134], but its cultivation was rather uncommon [121, 130, 135, 136]. Also according to isotope studies, C4 plants are known to comprise only an insignificant part of the human diet in Early Medieval Central Europa [22, 137], but was commonly consumed in Southern and Eastern Europe [e.g., 138–143], as well as in Northern Central Asia at the time [e.g., 144].

However, millet is known to be used as “back-up-crop” in case of poor harvest or crop failure of more commonly cultivated C3 crops (e.g., [124]) and archaeobotanical evidence from Early Medieval sites in Bavaria showed an isolated and unregular occurrence of millet [145]. Thus, the cultivation of millet in Southern Bavaria, at least temporarily, cannot be excluded (for details see S3.1 section in S3 Text). Such short-term use of millet can lead to an increase of δ^13^C for a short period, e.g., as seen in the nutritional profile from serial dentin samples of individual BWA_10075. In bone collagen, however, sporadic consumption of millet causes a slight increase of δ^13^C at most, since this represents a mixed signal spanning several years. The distinctly elevated δ^13^C values observed in bone values or in dentine values over several years of some individuals can only be explained by long-term consumption of a substantial amount of millet. This may have happened locally due to special circumstances (e.g., several years of continuous crop failure or exclusive consumption of traded food), or due to a previous residency in an area where the consumption of C4 plants was more common practice. Given the uneven distribution of millet consumption in Europe during this time, the latter is the most common interpretation of increased δ^13^C values in bones of isolated individuals in Early Medieval central European cemeteries (e.g., [124, 130, 146, 147]). Although extremely deviant dietary practice of individuals, which may have a cultural, religious, or pragmatic background, cannot entirely be excluded, there is little evidence for it in this time and region. Due to this, the archaeobotanical evidence and fact that increased δ^13^C values are most frequently found in women with ACD (see below), a change of residence seems to be the most likely explanation for elevated δ^13^C values in our sample as well.

The occasionally found elevated δ^15^N values in bone or dentin collagen on the other hand might be also a result of an individually different local diet, which is on average richer in animal proteins or includes more seafood. An origin from a region with a higher nitrogen baseline (such as marine coastal areas or terrestrial ecosystems with greater aridity or higher growth season temperatures [148–150]) can also be an explanation. This is likely to be the case for the elevated bone values of a woman AED_421. Her deviant isotopic signal compatible with a further marine diet together with her grave goods commonly found in Scandinavian regions showed a clear signal for a marine diet which strongly indicate a migration event (see Hakenbeck et al. [22]).

Overall, we believe that these observed association of highly deviant diets with foreign adjuncts or customs, suggest that isotopic values ranging clearly outside 99% of the population can be explained by change of residency of these individuals in most of the cases. This is especially likely if the C and N isotopes of dentin and bone in an individual differ substantially. As C and N isotopes from bone display a composite of the dietary intake over long periods (e.g., [49]), previous uncommon diets are masked. By including tooth dentin analysis, we were able to identify potential migrants, who would not have been detected by bone bulk analysis only.

#### Women with ACD

While ACD is a worldwide phenomenon, during the Migration period in Europe its spread is often associated with the expansion of the Hunnic Empire [24, 151]. For the 5^th^ and the beginning of the 6^th^ century, the findings of skulls with ACD are mainly centered in the Pannonian Basin, where more than half of the skulls found in graveyards show ACD. It was equally common among males and females and across all age classes [151, 152]. In contrast, only isolated individuals with ACD can be found in Bavarian burials of the late 5^th^ and early 6^th^ century AD and these are mainly adult females and never children or juveniles [51]. Already early on, this pattern was interpreted by some scholars as an indication that the isolated finds of ACD in Bavarian cemeteries around 500 were the remains of immigrant women from the East [24, 152, 153]. Veeramah et al. [23] have recently been able to show that these women have a strong genetic resemblance to present-day South-Eastern European populations, which was absent in individuals without ACD buried in the same graveyards. However, a deviant genetic ancestry alone may not be sufficient to infer a change of location during a person’s life, but this is also supported by our observation that all examined women with ACD in this study show signs of increased millet consumption in their childhood, and just some of them show such signals in their later life strongly supports this assumption. Early Medieval populations from the Pannonian basin show similar δ^13^C values (Hungary: [70, 142, 154, 155]; Romania: [98]) as well as some individuals with ACD from Croatia and Hungary [70, 156]. Additionally, the mean δ^15^N value in the bulk dentine of women with ACD was often slightly elevated, which also corresponds well with δ^15^N data of burials found in South-Eastern Europe (e.g., [70, 142, 154–156]).

Our serial dentine analysis provides interesting insights into the life history of women with ACD. One woman (BWA_10254*) who was buried at old age (over 60 years) shows elevated δ^13^C and δ^15^N values in childhood, but her bone value does not differ from the “common variability” (Fig 8 center right). A first remarkable dietary change of hers can be detected around the age of 15 years, which could be explained by a change of location, yet, neither the place of origin nor the place where she supposedly first moved to could have been Southern Bavaria. Her tooth isotope values before and after this event are far outside the “common variability”, indicating that she reached the region where she was buried, after the age of 20 years, when the formation of her wisdom tooth was completed. Whereas her bone collagen values inside the “common variability” indicated that her movement to Bavaria must have happened long before her death.

### Detecting migration and mobility by strontium

#### Local ranges

The geological conditions are relatively uniform in Southern Bavaria (Fig 2). This agrees with the fact that little variability exists in the local strontium isotope ratios of the different archaeological sites investigated. However, this is not the case for Regensburg (S2.3.3.2d Fig in S2 Text), what might be explained by its proximity to regions with elevated strontium isotope signatures, which are located just north of the Danube. The same is true for the site of Straubing, however, without showing a similar pattern. There are also indications that the strontium isotope values of the old town of Regensburg could be higher than the surroundings [9]. This area was built on Roman rubble, which might have influenced local strontium isotope ratios. To resolve this question, further analyses of environmental samples from the region would be necessary. Until then, the assessment of a local range for Regensburg should be considered preliminary.

It is noticeable that the determined local ranges derived from biomineral samples (reference as well as actual sample set), always exceed the range covered by the environmental samples. This may indicate that the local range is overestimated (Fig 3A–3D). However, only very few environmental samples per site are available, too few to capture the entire variability of ^87^Sr/^86^Sr on site (discussion of this aspect see Toncala et al. [54]). The local range in humans can also be increased by imported food sources. Using environmental samples only would not include this, but the distribution of the isotope values in the population (on which our determination of the local area is based on) does. Overall, the approach used here minimizes the risk of overestimating the number of non-local individuals but might increase in the risk of not recognizing non-local individuals.

#### Underestimation of human movement

The quite uniform geology and the use of rather broad local ranges results in small-scale mobility within South Bavaria staying mostly undetected. Furthermore, due to the geographical redundancy of the isotope values it is likely, that some individuals with "local" signature come from an area outside of Southern Bavaria with a similar geological substratum. Thus, the number of non-locals is most certainly underestimated regardless of the approach used to determine the local range. This becomes particularly clear in the case of women with modified skulls. Despite they most likely did not grow up in the region where they were buried, they show "local" ^87^Sr/^86^Sr isotope values. But their ^87^Sr/^86^Sr values can also be found in other parts of Europe, such as the Eastern European lowlands or the Eurasian steppe (e.g., Sjögreen et al. [58]).

#### Nature of human movement

Except a few individuals (6%, 95% CI: 3–10%), mainly non-locals originating from outside of the region between the Danube and the Alps were detected. The nearest areas with significantly deviating strontium isotope values outside the study area are quite close and north of the Danube (e.g., in the Bavarian Forest, ca. 140 km). Although geographically close, even immigration from these nearby regions into *Raetia II* meant crossing a cultural border, namely from Barbaricum to the former Roman province. Therefore, at least 17% (95% CI:12–23%) can be considered migrants (since migration is defined as long-term relocation of persons who cross cultural and/or political boundaries).

#### Extent of migration

An interpretation of the detected extent of migration is difficult. Comparisons with today’s migration rates or migration rates from other sites are not feasible. The number of migrants that can be reliably detected in a region not only depends on the number of individuals that actually migrated, but also on the geological and environmental conditions of both, region of origin and the burial site, as well as the type of data available and methods used to determine local isotope ranges. By taking archaeological sites from the Bavarian alpine foothill for comparison and calculating a local range that encompasses the entire region, we avoided some of the interpretation problems.

The depicted fluctuations of MFM over time (Fig 5) can be explained by both (1) variation in the actual number of non-locals, or (2) differences in the migrants’ region of origin. Since in different periods different places of origin might have dominated, which could have been detectable to varying degrees by means of strontium, the second explanation can never be excluded. But the average frequency, which equals the percentage of detectable migrants considering the individuals of all time periods together (red line in Fig 5) should balances out fluctuations based on differently detectable regions of origin. Because the rate of detectable immigrants for the second half of the 5^th^ century was not only well above this average but also exceeded any other time period, we suggest that an above-average migration rate is indeed evident for the North-Alpine foreland around 500 AD.

#### Regional differences in migration rates

The lower frequency of migrants in the border region (14%, 95% CI: 7–22%) indicates that, compared with the hinterland, immigration played a minor role there.

Only a few migrants were detected in the border region cemeteries of Straubing-Bajuwarenstraße (3%, 95% CI: 0–10%) and Irlmauth (14%, 95% CI: 0–36%) although Germanic grave goods were found there [37, 39]. This may support the hypothesis that the presence of Germanic grave goods is better explained by increased trade in goods, and only to a limited extent by migration. The site of Burgweinting show the highest proportion of identifiable migrants in the border region (24%, 95% CI:10–41%). Whether this rather high number, which is more comparable to those in the hinterland, can be related to a higher social status (as indicated by Codreanu-Windauer [36]) and thus to migration of elites (analogous to Unterhaching, see below) must remain unclear, since finds and grave goods have not yet been published in a complete and interpretable form. However, this site is also the furthest away from the former border and thus may not actually be subject to the dynamics of the border region.

A higher proportion of migrants in the hinterland (21%, 95% CI: 12–31%) supports the idea that the area was preferentially settled by newcomers, as also shown by 23% migrants (95% CI: 12–37%) in Altenerding. The highest proportion of migrants (33%, 95% CI: 0–67%) from outside Bavaria was found in the group of burials from Unterhaching. Including all non-locals, as much as 78% (95% CI: 44–100%) do not come from the region. The high proportion of non-locals among the group and the rich equipment of the graves suggests that these individuals belonged to an elite class that was sent to represent the new rulers of the region at the end of the 5^th^ century [43]. In Munich-Perlach, just 5 km away, we found a substantially lower frequency of migrants (13%, 95% CI: 0–26%). Consideration of the stable light isotope values suggests that individuals from Unterhaching are only found in the upper half of the data range for Munich-Perlach. The higher average of δ^15^N in Unterhaching could be explained by a higher intake of animal protein associated with a higher social status (e.g., [157, 158]) and might therefore be interpreted as a sign of the elite status of the individuals. An alternative explanation for the observed differences in stable light isotopes between Perlach and Unterhaching could be that subsistence strategies or environmental conditions were somewhat different in the region of origin of the non-locals detected in Unterhaching.

Again, it cannot be ruled out that the regional differences observed were caused, not only by actual differences in the number of migrants but also by the different detectability of the potential places of origin. Interestingly, at sites with a relatively low frequency of detectable migrants (PEL, STB, IRM) the δ^13^C values from bone bulk collagen were less variable than those at sites with a higher frequency of non-locals (BW, AED). Hence, the variability of carbon isotope ratios at excavation sites seems to reflect the frequency of non-locals.

Again, only the site of Unterhaching is an exception in this pattern. This anomaly can be explained as being due to the exceptionally low number of individuals analyzed from this site, or by the fact that the cemetery was the only one mainly occupied by non-local individuals. If all of those individuals came from an identical or similar region of origin, variation in values is not to be expected. This pattern suggests that different proportions of non-locals reflect real differences in the frequency of immigrants and are not caused by different origins.

### Identity of non-locals

#### Sex differences

Using strontium isotope analysis, we were able to identify not only women but also several men, as migrants. Indeed, in our study, the proportions of male and female migrants did not differ significantly (Fig 4A). However, it should be repeated here, that mobility between settlements within the study area is mostly not detectable with this approach and here gender related mobility rates might have been different. Overall, our results showed that in the Early Middle Ages, both sexes traveled to Southern Bavaria from outside of the former province *Raetia II*. Interesting in this context is, that we found strong deviating δ^13^C and δ^15^N values only in women. This indicates that men and women have partly migrated from different regions. Since we strongly assume that such a deviating diet is a sign of migration (migration, not mobility, because they must have come from a region outside of *Raetia II*), this would increase the proportion of women with a migration background.

#### Origin

Genetic associations and elevated δ^13^C values (indicating regular millet consumption) of women with ACD suggest an origin from Southeastern Europe as already discussed. This is also true for two other females (BW_10071, STB_310) without ACD [23].

The genetic ancestry of another female buried in Straubing (STB_300) with ^87^Sr/^86^Sr-values different from the study region and the region of origin of the women with ACD indicates an origin somewhere in Southern Europe [23].

For a woman who was buried in Altenerding (AED_421), a region of origin in Northern Europe has been suspected (see Hakenbeck et al. [22]).

Despite limitations on being able to define places of origin more precisely, the further observed variability of non-local ^87^Sr/^86^Sr but also of δ^13^C and δ^15^N values indicates that migrants originated from several different, geologically distinct areas and/or ecosystems with different food bases.

#### Life-stage when moving

We assume that the observed distinct changes in the diet of some women, especially if it happens quite abruptly, represent a change in residence in most cases, it is possible to reconstruct at what life stage the movement took place. For instance, some females (BWA_10254*, AED_343, PEL_12) show an “uncommon” diet in childhood but do not differ from native locals in their bone values. This indicates they grew up elsewhere and immigrated to the region a long time before they were buried there. As their bone value became indistinguishable from the rest of the local population, they apparently adopted common dietary habits.

In contrast, four females (AED_421, AED_513*, AED_1108*, STB_228) were identified as “newcomers”, with diverging stable isotope ratios in their bones. Since they were of late adult or senior age at the time of their death. These women moved to Bavaria at rather advanced ages, they must have moved to Bavaria at rather advanced ages. These women were most likely past the reproductive phase, which contests marriage migration as the only reason for a change of location of women.

Serial sampling of the dentin from M1 to M3 sometimes allows even further delineation of the time of change of residence. The isotope profile of one female (AED_10071) revealed a dietary change around the age of 15 years, which can be associated with migration into Southern Bavaria.

This clearly demonstrates the complexity of women’s life histories during the Early Middle Ages in Bavaria.

## Conclusion

Regardless of how high the actual immigration rate may have been, this study shows that migrants increasingly entered the former province of *Raetia II* towards the end of the 5^th^ century. Therefore, the assumption that only marginal migration took place during this time can be rejected. Our results correspond well with archaeological evidence regarding the continuity of settlement at the provincial borders, and the profound changes that affected settlement structures in more distant rural areas. We could also show that both sexes undertook migration. Furthermore, the reconstructed life histories of some women hint at the diversity of female life paths during the Early Middle Ages.

Emerging differences in the mobility among sexes, as well as the finding that individuals migrated from a diversity of regions of origin, suggests that immigration and mobility in *Raetia II* after the fall of the Roman Empire was highly complex, and will likely only gradually be uncovered.

## Supporting information

S1 Text Data set. (XLSX)

S2 Text Detailed methods. (DOCX)

S3 Text Statistics and detailed results. (DOCX)
