# Supplementary material for: Between Raetia Secunda and the dutchy of Bavaria: Exploring patterns of human movement and diet
Source: PLoS One. 2023 Apr 5;18(4):e0283243. doi: 10.1371/journal.pone.0283243 (PMC10075417; doi:10.1371/journal.pone.0283243)
Supplement: S2 Text — (DOCX) [file pone.0283243.s002.docx]

# S2 Detailed methods

## S2.1 Indication from previous studies and their limits

Veeramah et al. [1] generated genomic data from around 40 individuals from several burial grounds in the region. The study showed that men generally had a fairly homogenous ancestry, similar to modern Northern and Central Europeans. By contrast, women exhibited a very high genetic heterogeneity. Furthermore, Veeramah et al. [1] demonstrated that in most cases, women buried in Bavaria who have modified skulls (ACD) showed a strong genetic resemblance to present-day South-Eastern European populations that was absent in individuals without ACD. However, genetic ancestry data alone is not sufficient to infer a change of location during a person’s life because genetic differentiation may be the result of an earlier ancestry. Furthermore, due to natural human mobility and associated gene flow, it is difficult to categorically define even stationary populations, and clearly assign them to geographical, national, social, or cultural boundaries. The genetic differences between populations are almost always fluid, and can only be detected at greater geographical distances.

Hakenbeck et al. [2] determined carbon and nitrogen isotope values from the bone bulk collagen of various individuals from Early Medieval Bavaria. They found evidence of an alternative diet in 5% of the individuals tested, most of whom were women with ACD. Increased δ^13^C values in bone collagen of these individuals indicated frequent consumption of millet, a C4 plant which is known to comprise only a minor part of human diet in Early Medieval Bavaria (e.g., [3-4]). However, millet was more commonly eaten in Southern and Eastern Europe (e.g., [5-10]), as well as in Northern Central Asia at the same time (e.g., [11]). C4 plants such as millet are adapted to the warm and dry climatic conditions not found in Central Europe, and are therefore suitable indicators for different ecotopes. As the authors concluded, if an alternative diet is taken to be an indicator of immigration, then the proportion of immigrants appears to have been rather low and predominantly female. However, Hakenbeck et al. [2] point out that there are many regions in Europe (and worldwide) that share similar isotopic signatures (see the new database of Cocozza et al. [12]). Individuals originating from regions where isotopic signatures differ from those of the burial site can be identified as allochthonous, but migration from one land-locked region to another within temperate Europe may have no noticeable effect on dietary bone isotope data [4, 13]. Furthermore, it must be considered that bones, such as those used in the study by Hakenbeck et al. [2], are remodeled throughout life. Thus, they only record isotope signals from later life stages of individuals [14-17]. Consequently, only migrants whose isotope levels have not yet equalized with local conditions are likely to be identified by analyzing bones.

## S2.2 Sampling of serial dentine sections

### S2.2.1 Cutting of demineralized dentine

The demineralized dentine of a tooth half is placed in a rail, so that the occlusal crown part sits directly against the wall of the rail, and the straight cutting surface rests on the bottom of the rail. The blade of a scalpel is guided through the recess while applying light pressure on the tooth half to hold it in position, to cut dentine slices. The distance between the inner wall of the rail and the middle of the recess measures 1 mm.


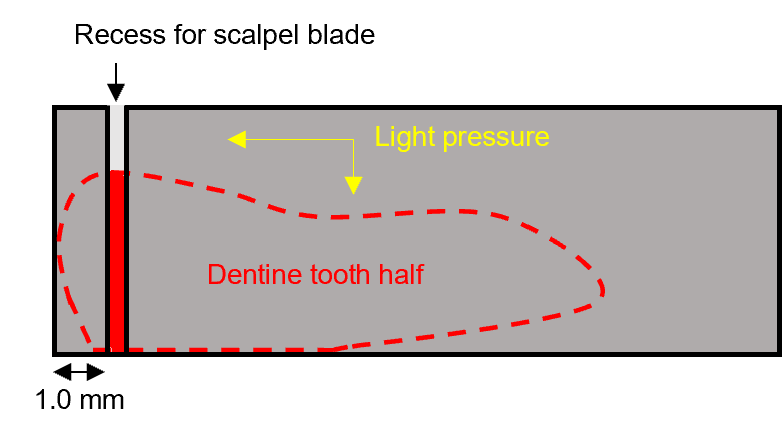


*Figure S2.2.1: Schematic construction of cutting device for tooth dentine. Constructed by Ferdinand Neuberger.*

### S2.2.2 Estimating chronological age of dentine sections

Sectioning is performed in a linear way, cutting the demineralized dentine horizontally into equal 1 mm thick sections, only differentiating between crown sections and root sections. Since teeth show a biological variation in size, we do not have equal number of sections for each tooth and sections do not represent single formation stages. To assign those sections individual age spans, we developed a scheme based on the *London Atlas of Human Tooth Development and Eruption* of AlQahtani et al. [18]. They analyzed 704 radiographs of known age individuals and known age at death skeletal remains and documented minimum (Min), maximum (Max) and median (Med) tooth formation stages for defined ages (midpoint of three months for individuals from 1 week to <12 month or midpoint of one year for individuals from 1 to <24 years, sexes were combined). Table S2.2.2.1 shows the developmental stages of multiple rooted teeth modified according to Moorrees et al. [19-20] (similar to Fig 2 in AlQahtani et al. [18]).

*Table S2.2.2.1: Development stages of multiple rooted teeth modified according to Moorrees et al. [19-20].*

| 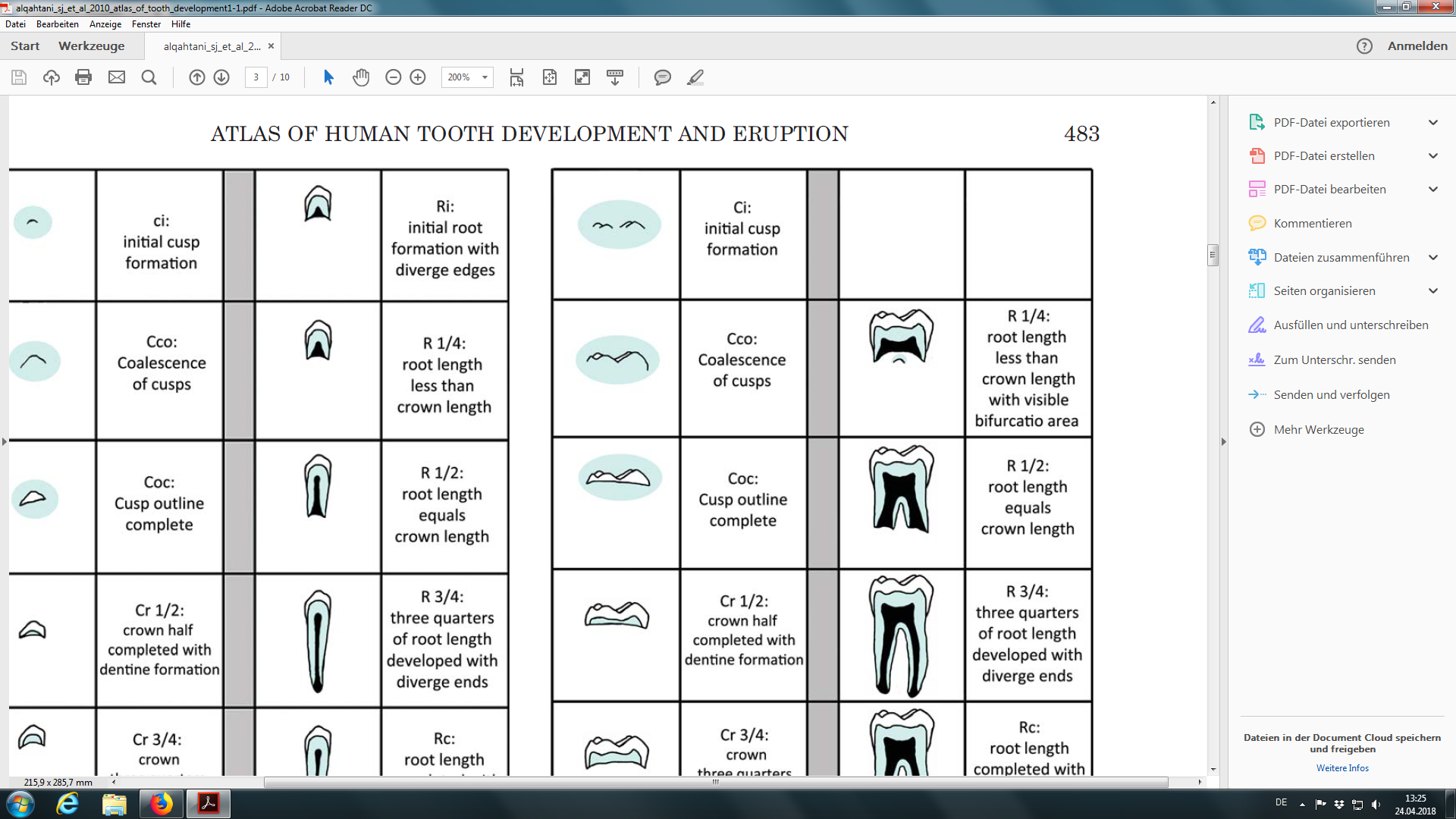 | Ci: initial cusp formation | 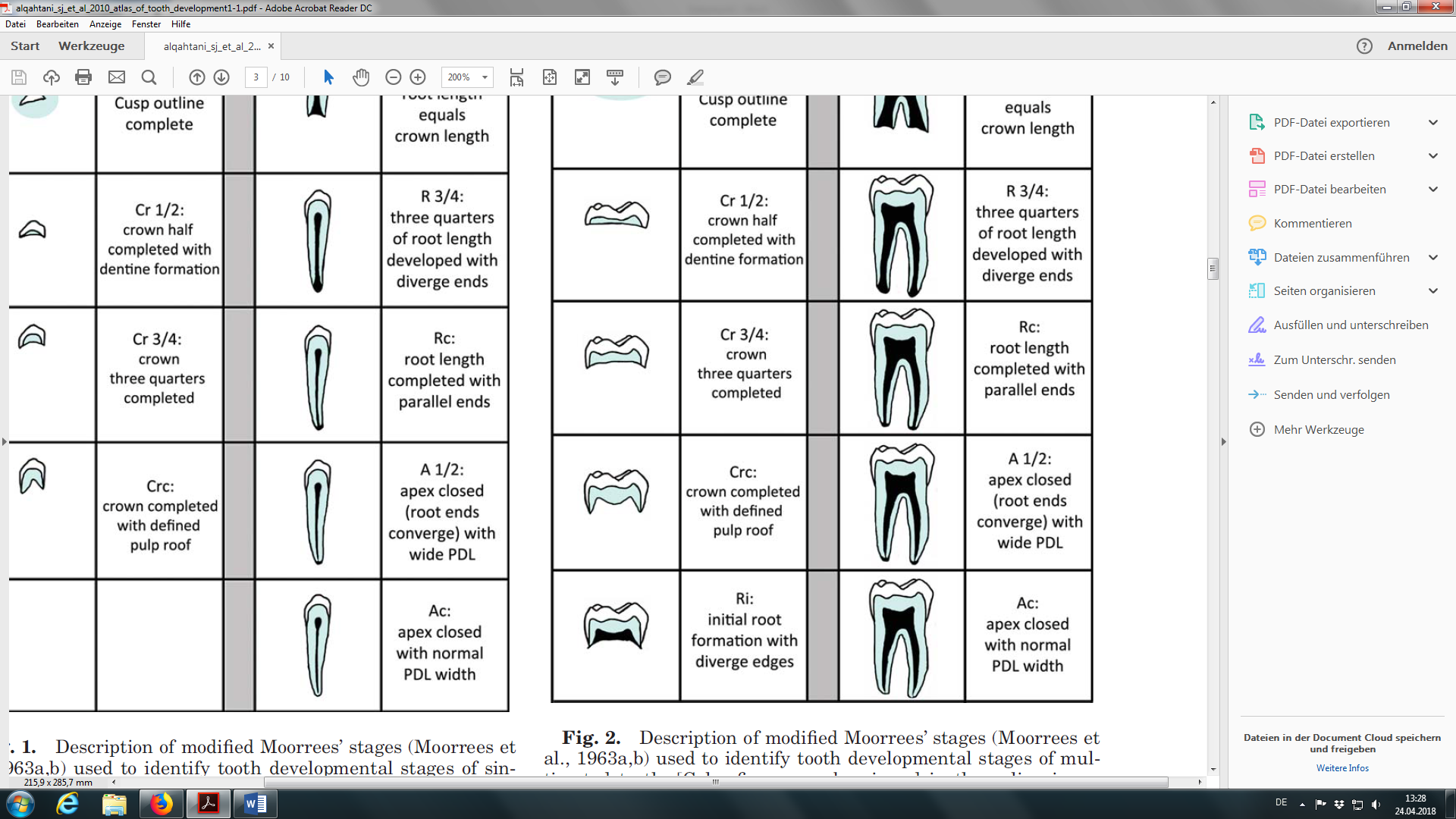 | Ri: initial root formation with diverge edges |
| --- | --- | --- | --- |
| 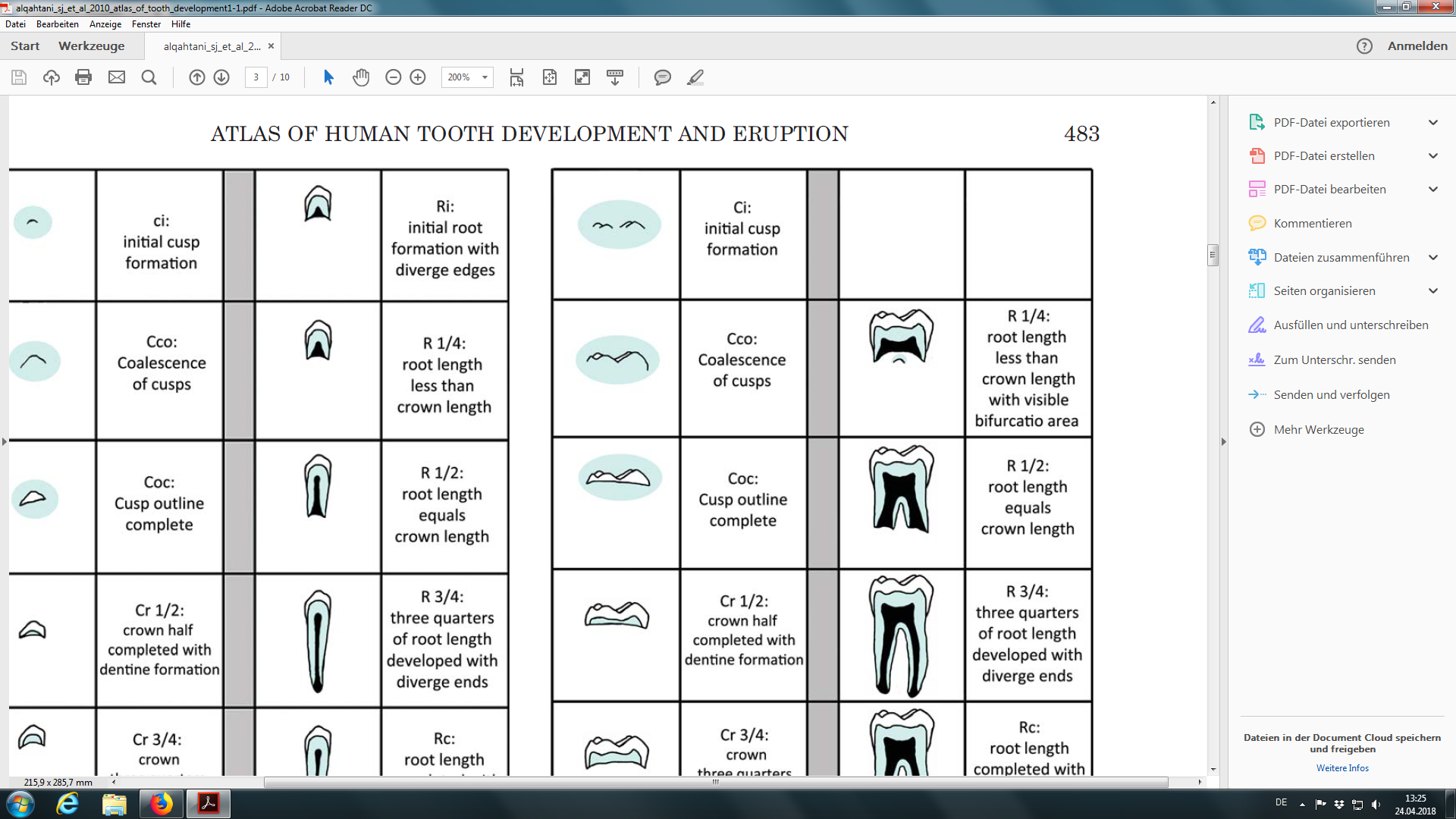 | Cco: coalescence of cusps | 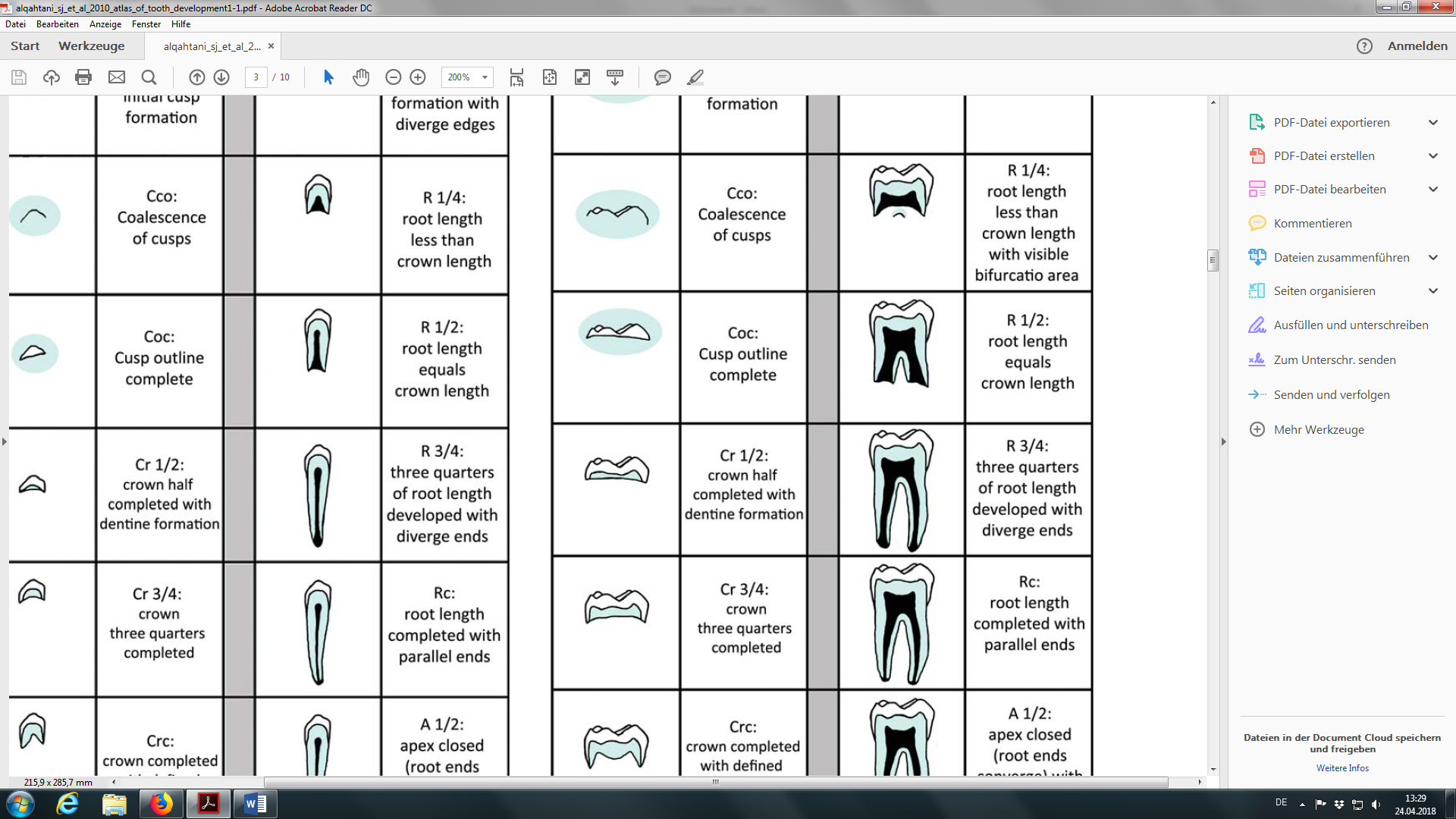 | R¼: root length less than crown length with visible bifurcation area |
| 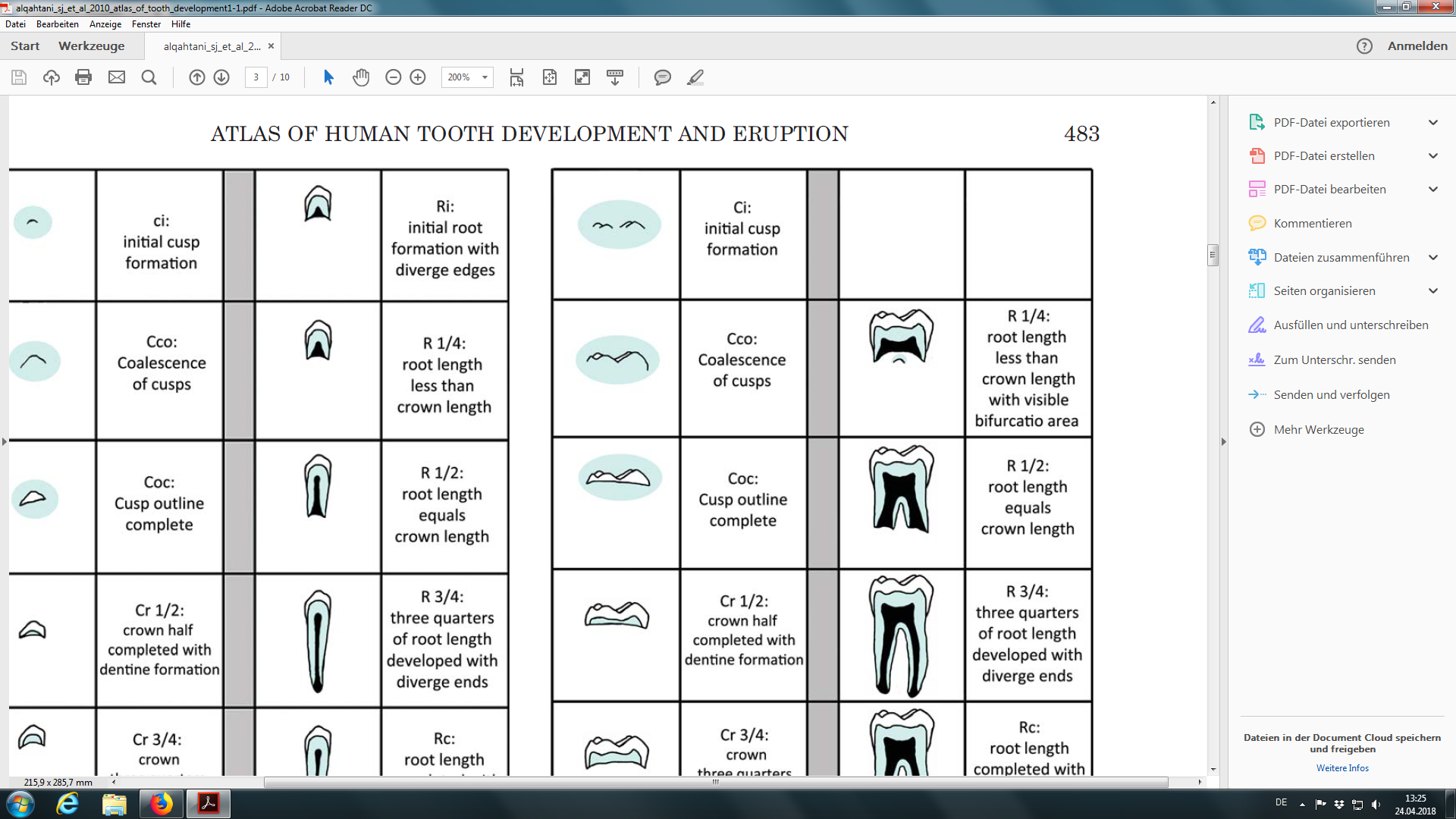 | Coc: cusp outline complete | 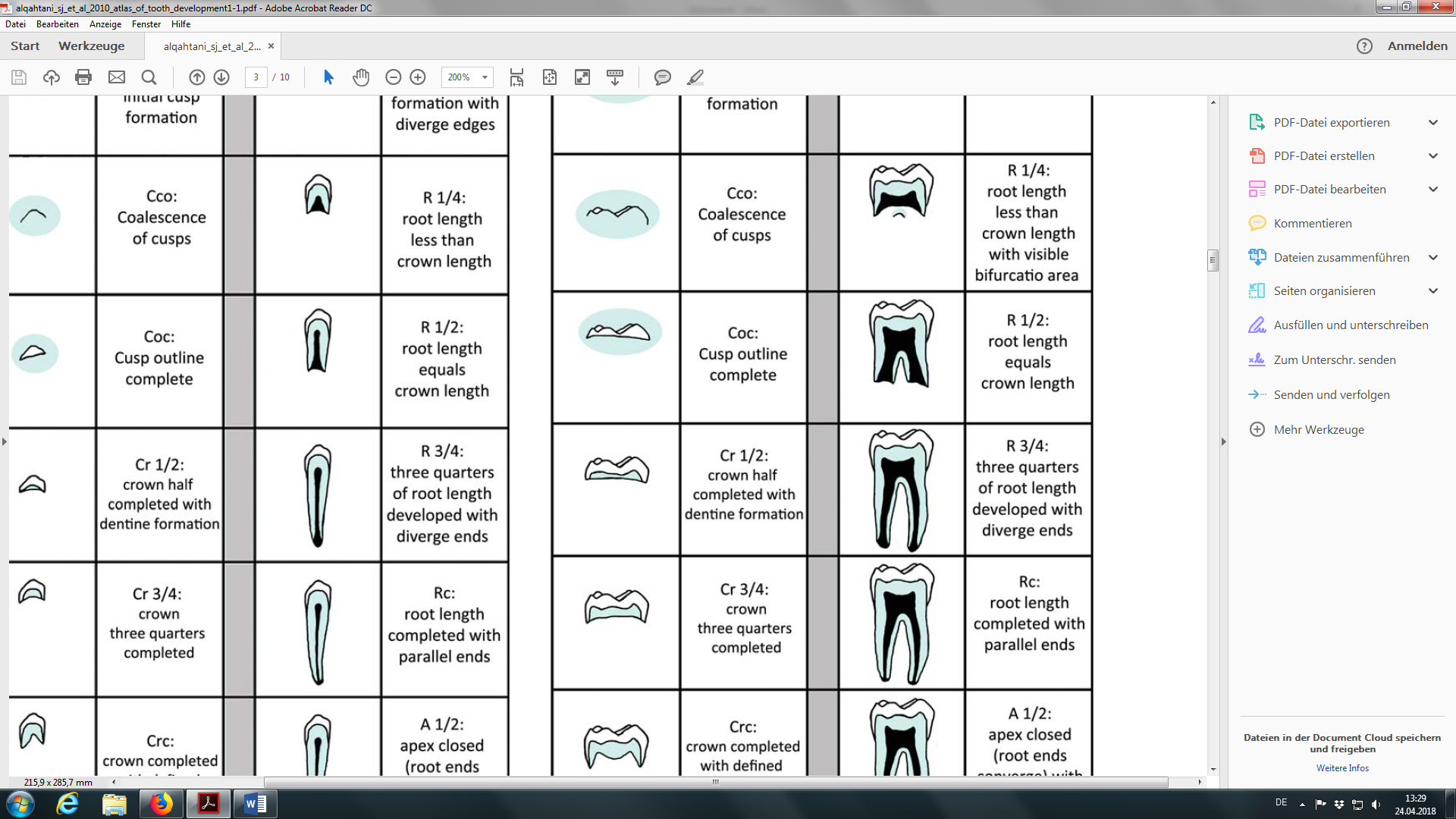 | R½: root length equals crown length |
| 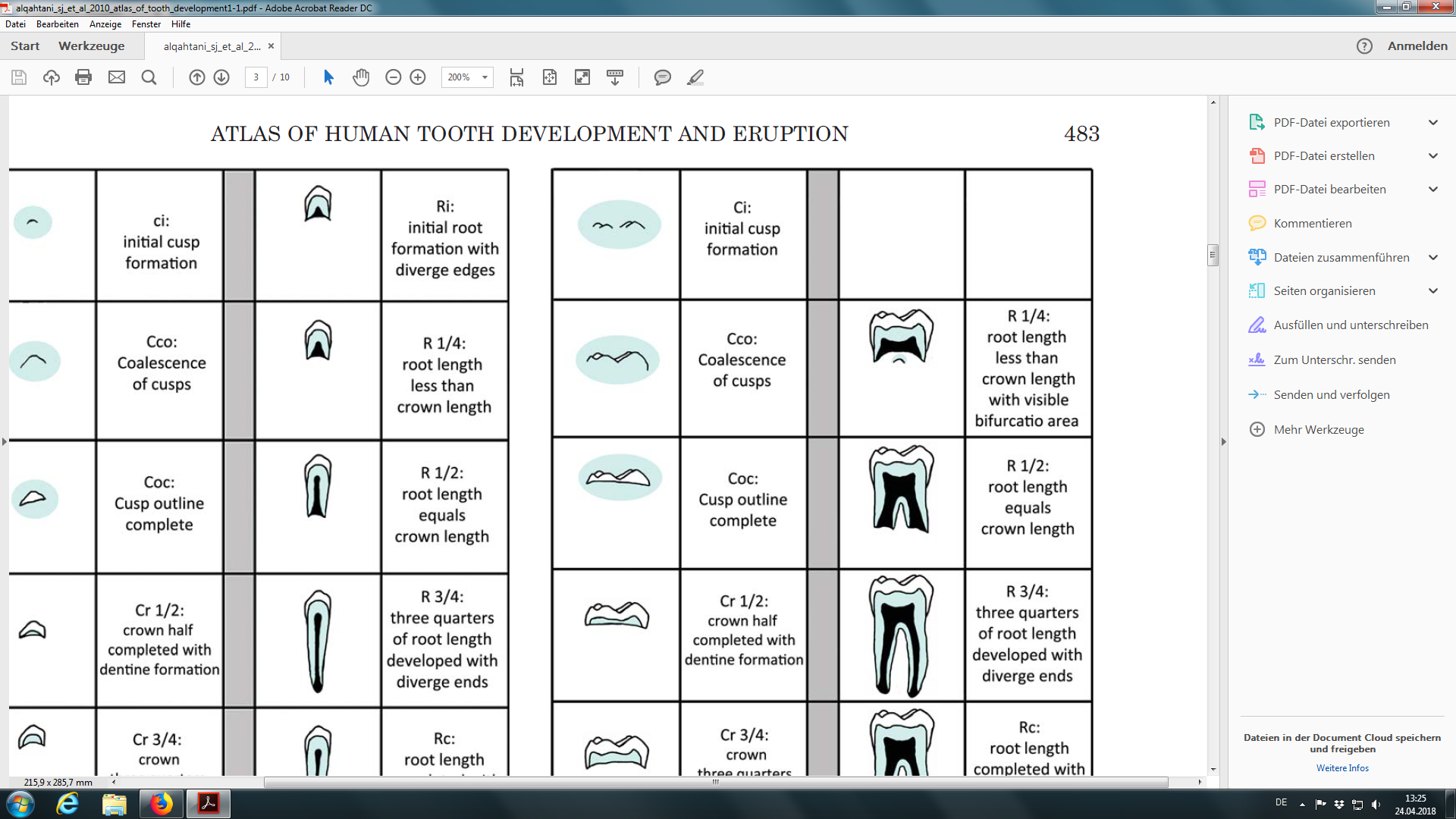 | Cr½: crown half completed with dentine formation | 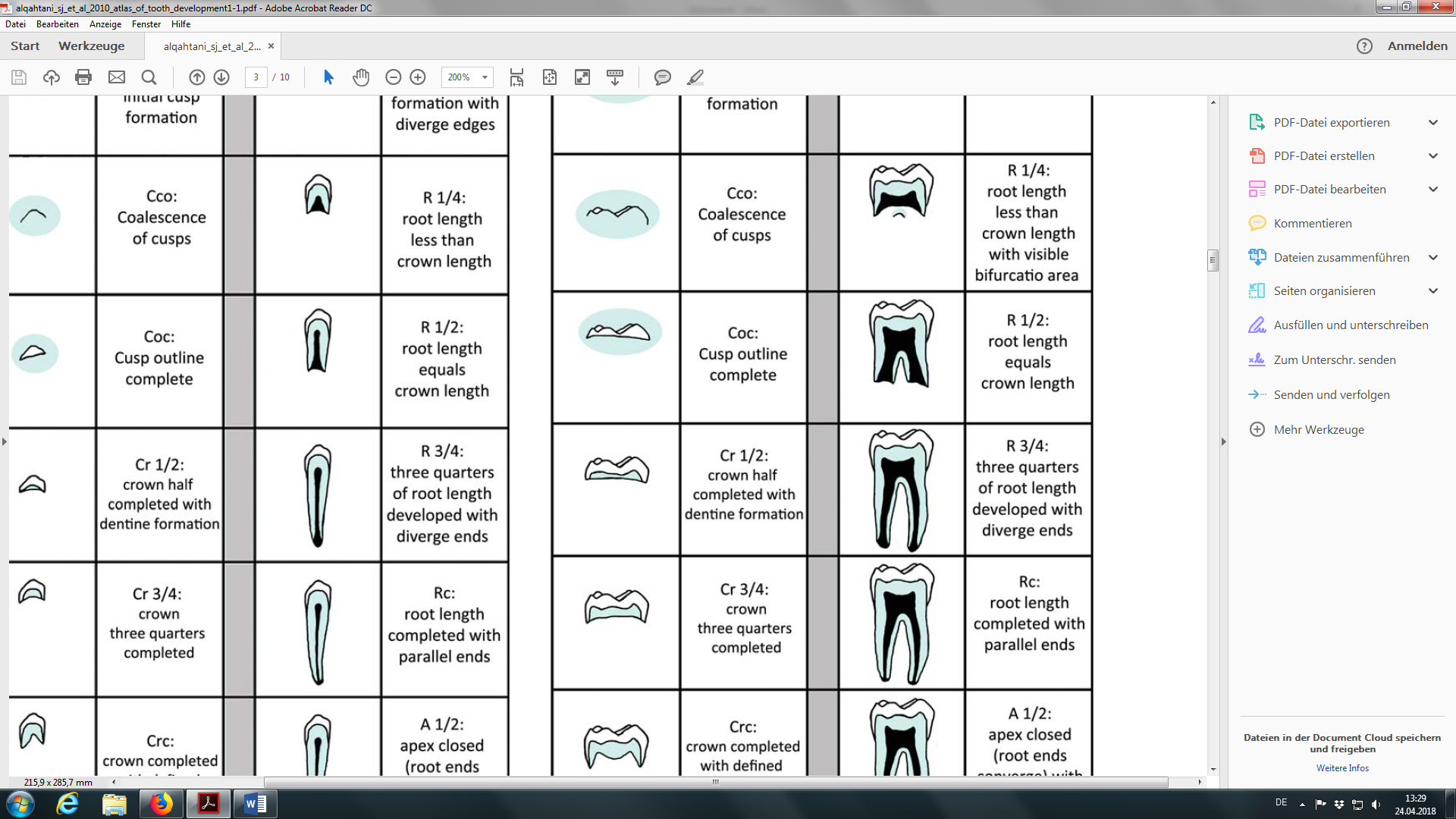 | R¾: three quaters of root length developed with diverge ends |
| 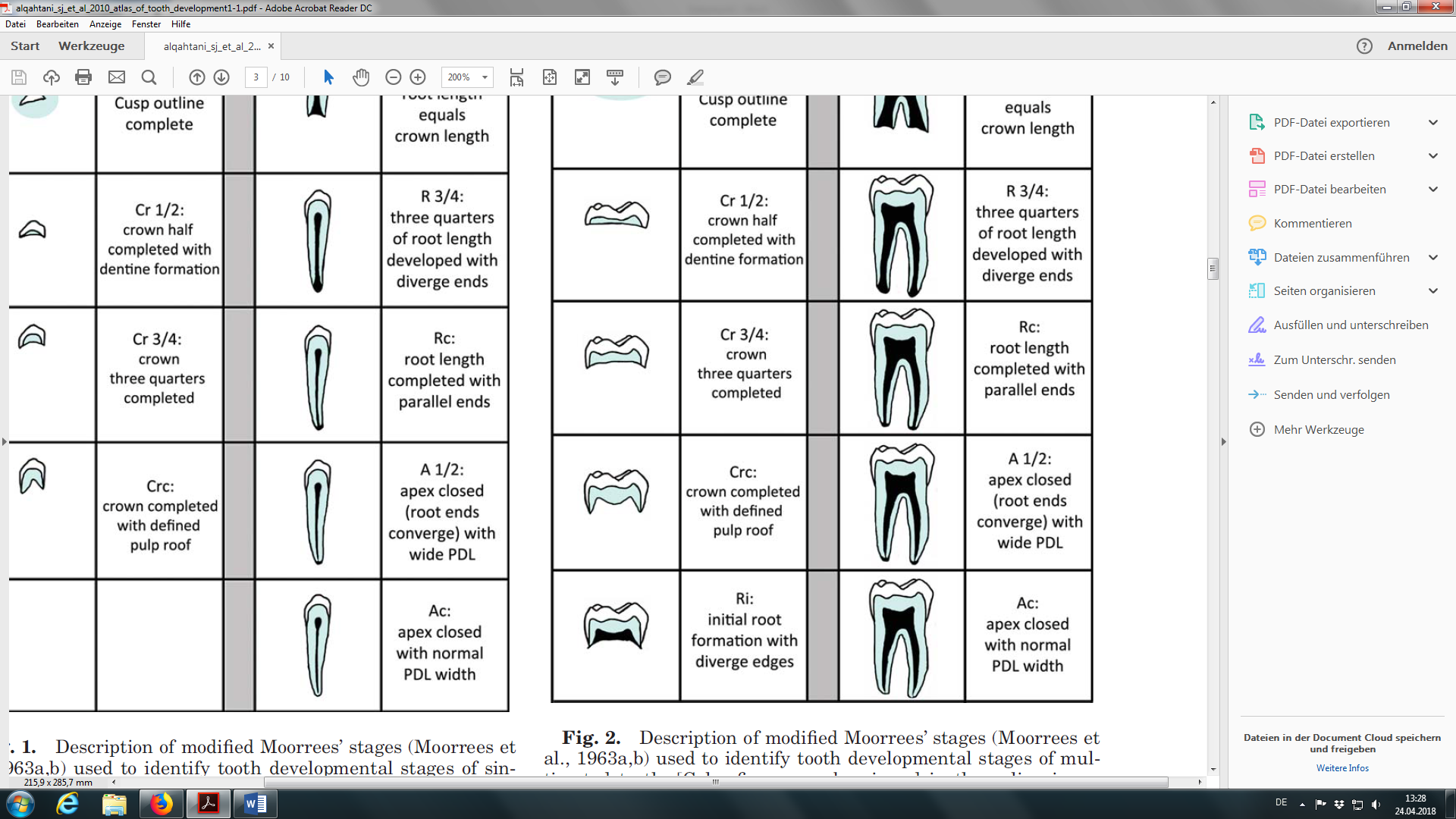 | Cr¾: crown three quarters completed | 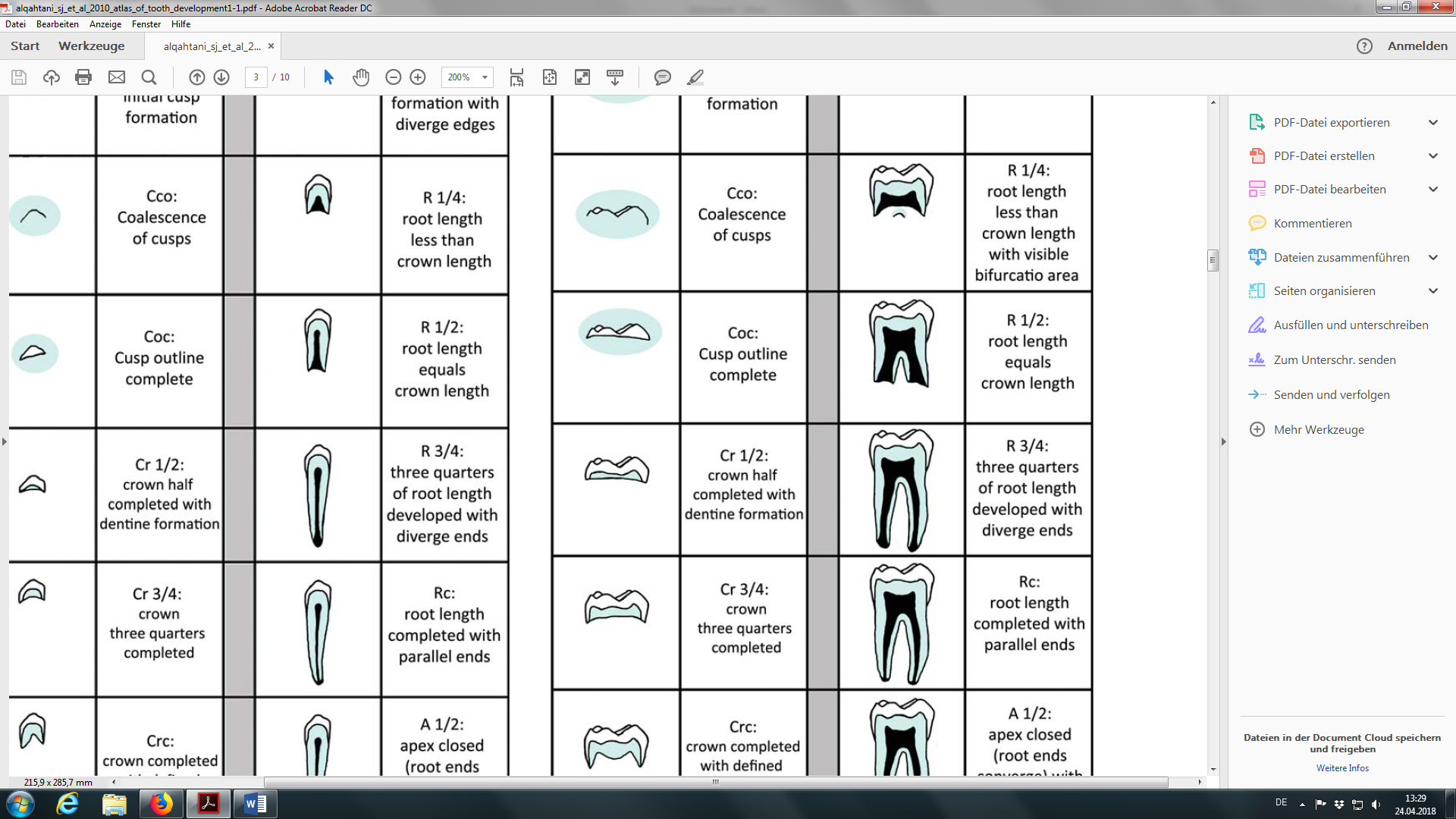 | Rc: root legth completed with parallel ends |
| 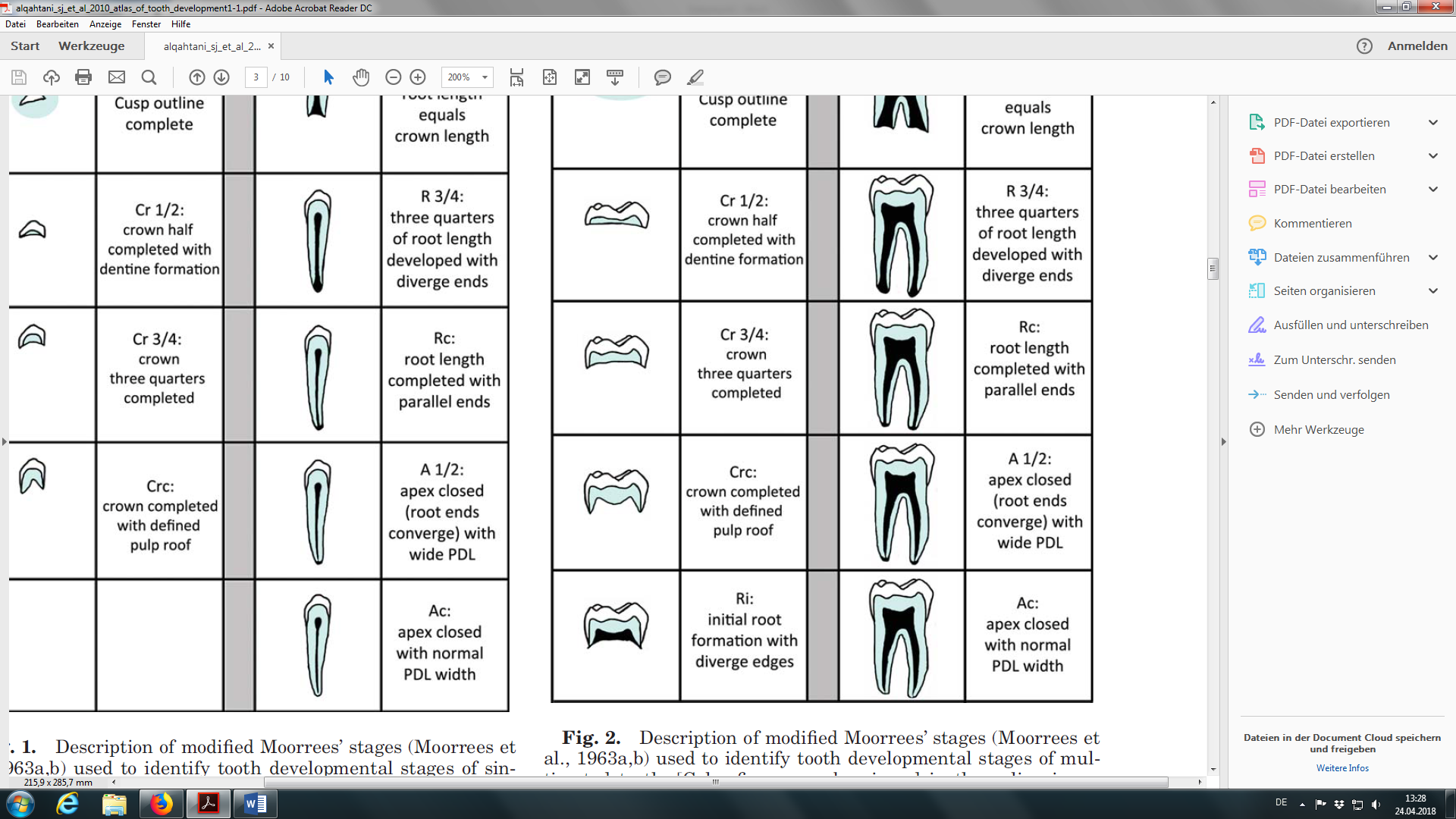 | Crc: crown completed with defined pulp roof | 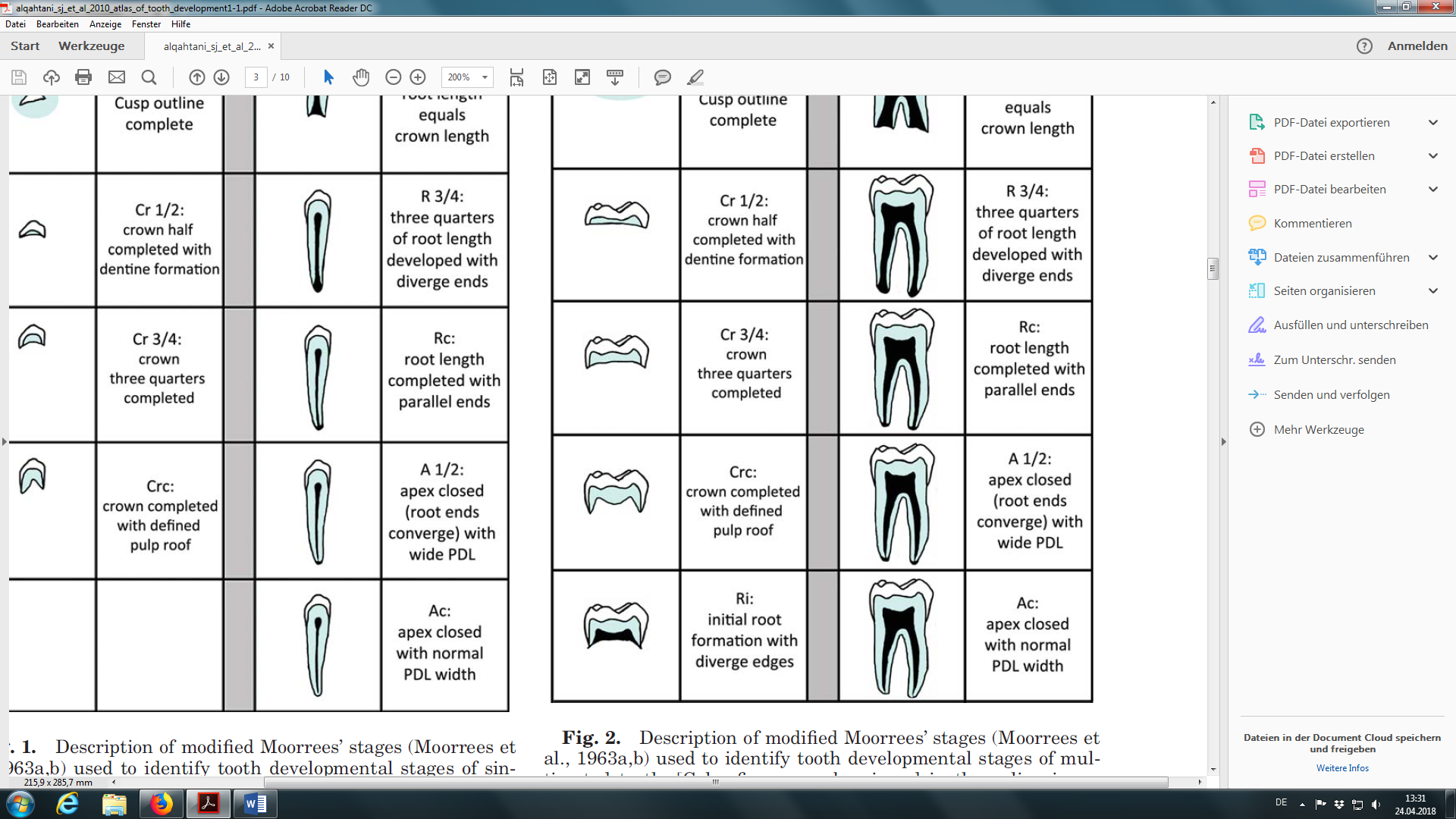 | A½: apex closed (root ends converge) with wide PDL (peridontal ligament space) |
|  | | 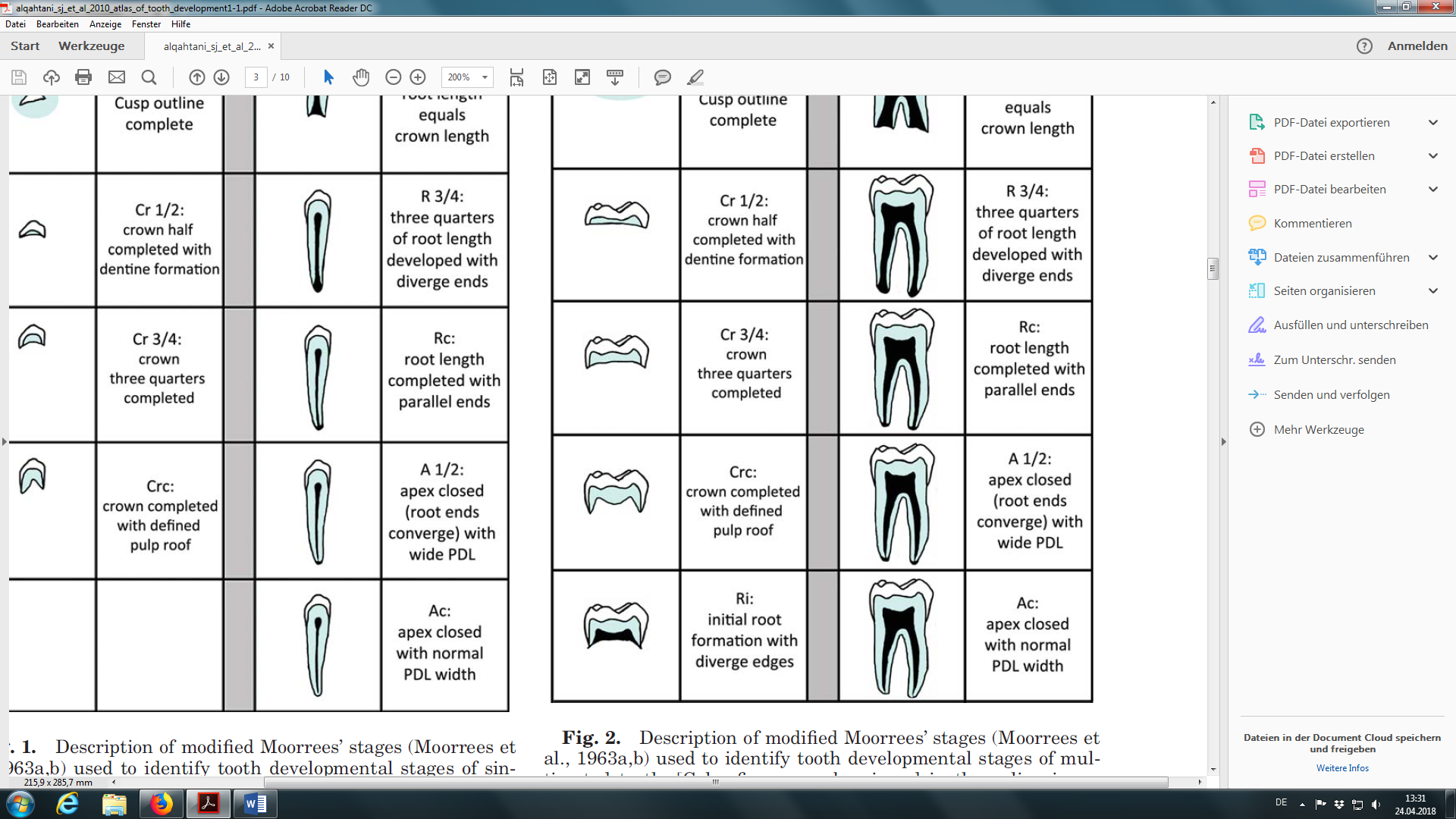 | Ac: apex closed with normal PDL width |

Table S2.2.2.2a–c shows information of Table 3–9 in AlQahtani et al. [18] for mandibular and maxillary molars.

*Table S2.2.2.2a: Combined sex tooth development data of the first mandibular and maxillary molar.*

| M1 |  | Formation stage | | | | | | | | | | | | | | | | | | | | | | | | | | |
| --- | --- | --- | --- | --- | --- | --- | --- | --- | --- | --- | --- | --- | --- | --- | --- | --- | --- | --- | --- | --- | --- | --- | --- | --- | --- | --- | --- | --- |
| Jaw part | Age [years] | Ci | | | | Cco | | Coc | | Cr½ | Cr¾ | | Crc | Ri | | R¼ | | R½ | R¾ | | Rc | A½ | Ac | | | | | |
| Mandibula | 0.125** |  | | | | Max | |  | |  |  | |  |  | |  | |  |  | |  |  |  | | | | | |
|  | 0.375** | Min | | Med | | Max | |  | |  |  | |  |  | |  | |  |  | |  |  |  | | | | | |
|  | 0.625** |  | | | | Min | | Med | Max |  |  | |  |  | |  | |  |  | |  |  |  | | | | | |
|  | 0.875** |  | | | | Min | | Med | Max |  |  | |  |  | |  | |  |  | |  |  |  | | | | | |
|  | 1.5* |  | | | | Min | | Med | | Max |  | |  |  | |  | |  |  | |  |  |  | | | | | |
|  | 2.5* |  | | | |  | |  | | Min | Med | | Max |  | |  | |  |  | |  |  |  | | | | | |
|  | 3.5* |  | | | |  | |  | | Min |  | |  | Med | Max |  | |  |  | |  |  |  | | | | | |
|  | 4.5* |  | | | |  | |  | |  |  | |  |  | | Min | Med | Max |  | |  |  |  | | | | | |
|  | 5.5* |  | | | |  | |  | |  |  | |  |  | | Min | Med | Max |  | |  |  |  | | | | | |
|  | 6.5* |  | | | |  | |  | |  |  | |  |  | | Min | | Med | Max | |  |  |  | | | | | |
|  | 7.5* |  | | | |  | |  | |  |  | |  |  | |  | |  | Min | Med |  | Max |  | | | | | |
|  | 8.5* |  | | | |  | |  | |  |  | |  |  | |  | |  | Min | Med |  | Max |  | | | | | |
|  | 9.5* |  | | | |  | |  | |  |  | |  |  | |  | |  | Min | |  | Med | Max | | | | | |
|  | 10.5* |  | | | |  | |  | |  |  | |  |  | |  | |  |  | | Min |  | Med | | | | Max | |
|  | 11.5* |  | | | |  | |  | |  |  | |  |  | |  | |  |  | |  | Min | Med | | | | Max | |
|  | 12.5* |  | | | |  | |  | |  |  | |  |  | |  | |  |  | |  |  | Min | Med | | | | Max |
| Maxilla | 0.125** | Max | | | |  | |  | |  |  | |  |  | |  | |  |  | |  |  |  | | | | | |
|  | 0.375** | Min | Med | | Max |  | |  | |  |  | |  |  | |  | |  |  | |  |  |  | | | | | |
|  | 0.625** |  | | | | Min | Med | Max | |  |  | |  |  | |  | |  |  | |  |  |  | | | | | |
|  | 0.875** |  | | | | Min | | Med | Max |  |  | |  |  | |  | |  |  | |  |  |  | | | | | |
|  | 1.5* |  | | | |  | | Med | | Max |  | |  |  | |  | |  |  | |  |  |  | | | | | |
|  | 2.5* |  | | | |  | |  | | Min | Med | Max |  |  | |  | |  |  | |  |  |  | | | | | |
|  | 3.5* |  | | | |  | |  | | Min |  | | Med |  | | Max | |  |  | |  |  |  | | | | | |
|  | 4.5* |  | | | |  | |  | |  |  | |  |  | | Min | Med | Max |  | |  |  |  | | | | | |
|  | 5.5* |  | | | |  | |  | |  |  | |  |  | | Min | Med | Max |  | |  |  |  | | | | | |
|  | 6.5* |  | | | |  | |  | |  |  | |  |  | | Min | | Med | Max | |  |  |  | | | | | |
|  | 7.5* |  | | | |  | |  | |  |  | |  |  | | Min | |  | Med | |  | Max |  | | | | | |
|  | 8.5* |  | | | |  | |  | |  |  | |  |  | |  | | Min |  | | Med |  | Max | | | | | |
|  | 9.5* |  | | | |  | |  | |  |  | |  |  | |  | |  |  | | Min |  | Med | | | Max | | |
|  | 10.5* |  | | | |  | |  | |  |  | |  |  | |  | |  |  | | Min |  | Med | | | Max | | |
|  | 11.5* |  | | | |  | |  | |  |  | |  |  | |  | |  |  | |  | Min | Med | | | Max | | |
|  | 12.5* |  | | | |  | |  | |  |  | |  |  | |  | |  |  | |  |  | Min | | Med | | | Max |
| ** Midpoint of three month  * Midpoint of one year | | | | | | | | | | | | | | | | | | | | | | | | | | | | |

*Table S2.2.2.2b: Combined sex tooth development data of the second mandibular and maxillary molar.*

| M2 |  | Formation stage | | | | | | | | | | | | | | | | | | | |
| --- | --- | --- | --- | --- | --- | --- | --- | --- | --- | --- | --- | --- | --- | --- | --- | --- | --- | --- | --- | --- | --- |
| Jaw | Age [years]* | Ci | | Cco | Coc | Cr½ | Cr¾ | Crc | Ri | R¼ | R½ | | R¾ | Rc | | A½ | Ac | | | | |
| Mandibula | 2.5 | Med | Max |  |  |  |  |  |  |  |  | |  |  | |  |  | | | | |
|  | 3.5 |  | | Med | Max |  |  |  |  |  |  | |  |  | |  |  | | | | |
|  | 4.5 |  | | Min | Med | Max |  |  |  |  |  | |  |  | |  |  | | | | |
|  | 5.5 |  | |  | Min | Med | Max |  |  |  |  | |  |  | |  |  | | | | |
|  | 6.5 |  | |  | Min | Med |  | Max |  |  |  | |  |  | |  |  | | | | |
|  | 7.5 |  | |  |  | Min | Med |  |  | Max |  | |  |  | |  |  | | | | |
|  | 8.5 |  | |  |  |  | Min |  | Med | Max |  | |  |  | |  |  | | | | |
|  | 9.5 |  | |  |  |  |  |  | Min | Med | Max | |  |  | |  |  | | | | |
|  | 10.5 |  | |  |  |  |  |  |  | Min | Med | Max |  |  | |  |  | | | | |
|  | 11.5 |  | |  |  |  |  |  |  | Min | Med | | Max |  | |  |  | | | | |
|  | 12.5 |  | |  |  |  |  |  |  | Min |  | | Med | Max | |  |  | | | | |
|  | 13.5 |  | |  |  |  |  |  |  |  | Min | | Med |  | | Max |  | | | | |
|  | 14.5 |  | |  |  |  |  |  |  |  |  | |  | Min | Med |  | Max | | | | |
|  | 15.5 |  | |  |  |  |  |  |  |  |  | |  | Min | | Med | Max | | | | |
|  | 16.5 |  | |  |  |  |  |  |  |  |  | |  |  | | Min | Med | | | Max | |
|  | 17.5 |  | |  |  |  |  |  |  |  |  | |  |  | |  | Min | Med | | | Max |
| Maxilla | 2.5 | Med | Max |  |  |  |  |  |  |  |  | |  |  | |  |  | | | | |
|  | 3.5 |  | | Med | Max |  |  |  |  |  |  | |  |  | |  |  | | | | |
|  | 4.5 | Min | |  | Med | Max |  |  |  |  |  | |  |  | |  |  | | | | |
|  | 5.5 |  | |  | Min | Med | Max |  |  |  |  | |  |  | |  |  | | | | |
|  | 6.5 |  | |  | Min | Med |  | Max |  |  |  | |  |  | |  |  | | | | |
|  | 7.5 |  | |  |  | Min | Med |  |  | Max |  | |  |  | |  |  | | | | |
|  | 8.5 |  | |  |  |  |  | Min | Med | Max |  | |  |  | |  |  | | | | |
|  | 9.5 |  | |  |  |  |  |  | Min | Med | Max | |  |  | |  |  | | | | |
|  | 10.5 |  | |  |  |  |  |  |  | Min | Med | Max |  |  | |  |  | | | | |
|  | 11.5 |  | |  |  |  |  |  |  | Min | Med | |  | Max | |  |  | | | | |
|  | 12.5 |  | |  |  |  |  |  |  | Min |  | | Med | Max | |  |  | | | | |
|  | 13.5 |  | |  | Min |  |  |  |  |  |  | | Med |  | | Max |  | | | | |
|  | 14.5 |  | |  |  |  |  |  |  |  |  | |  | Min | Med |  | Max | | | | |
|  | 15.5 |  | |  |  |  |  |  |  |  |  | |  | Min | | Med | Max | | | | |
|  | 16.5 |  | |  |  |  |  |  |  |  |  | |  |  | | Min | Med | | | Max | |
|  | 17.5 |  | |  |  |  |  |  |  |  |  | |  |  | |  | Min | | Med | | Max |
| * Midpoint of one year | | | | | | | | | | | | | | | | | | | | | |

*Table S2.2.2.2c: Combined sex tooth development data of the third mandibular and maxillary molar.*

| M3 |  | Formation stage | | | | | | | | | | | | | | | | | | |
| --- | --- | --- | --- | --- | --- | --- | --- | --- | --- | --- | --- | --- | --- | --- | --- | --- | --- | --- | --- | --- |
| Jaw | Age [years] | Ci | Cco | Coc | Cr½ | Cr¾ | Crc | Ri | R¼ | | | R½ | R¾ | Rc | A½ | | | Ac | | |
| Mandibula | 7.5 | Max |  |  |  |  |  |  |  | | |  |  |  |  | | |  | | |
|  | 8.5 | Med | Max |  |  |  |  |  |  | | |  |  |  |  | | |  | | |
|  | 9.5 |  | Med |  |  | Max |  |  |  | | |  |  |  |  | | |  | | |
|  | 10.5 |  | Med |  | Max |  |  |  |  | | |  |  |  |  | | |  | | |
|  | 11.5 | Min |  | Med |  |  |  |  | Max | | |  |  |  |  | | |  | | |
|  | 12.5 | Min |  |  | Med |  |  |  | Max | | |  |  |  |  | | |  | | |
|  | 13.5 | Min |  |  | Med |  |  |  | Max | | |  |  |  |  | | |  | | |
|  | 14.5 |  |  |  | Min |  |  |  | Med | Max | |  |  |  |  | | |  | | |
|  | 15.5 |  |  |  | Min |  |  |  | Med | | |  | Max |  |  | | |  | | |
|  | 16.5 |  |  |  |  |  | Min |  |  | | | Med | Max |  |  | | |  | | |
|  | 17.5 |  |  |  |  |  |  |  | Min | | | Med |  | Max |  | | |  | | |
|  | 18.5 |  |  |  |  |  |  |  | Min | | |  | Med | Max |  | | |  | | |
|  | 19.5 |  |  |  |  |  |  |  | Min | | |  |  | Med | Max | | |  | | |
|  | 20.5 |  |  |  |  |  |  |  |  | | |  | Min |  | Med | Max | |  | | |
|  | 21.5 |  |  |  |  |  |  |  |  | | |  |  | Min | Med | | | Max | | |
|  | 22.5 |  |  |  |  |  |  |  |  | | |  |  | Min | Med | | | Max | | |
|  | 23.5 |  |  |  |  |  |  |  |  | | |  |  |  |  | | | Min | Med | Max |
| Maxilla | 7.5 | Max |  |  |  |  |  |  |  | | |  |  |  |  | | |  | | |
|  | 8.5 | Med |  | Max |  |  |  |  |  | | |  |  |  |  | | |  | | |
|  | 9.5 |  |  | Med |  | Max |  |  |  | | |  |  |  |  | | |  | | |
|  | 10.5 |  |  | Med | Max |  |  |  |  | | |  |  |  |  | | |  | | |
|  | 11.5 | Min |  |  | Med |  |  | Max |  | | |  |  |  |  | | |  | | |
|  | 12.5 |  | Min |  |  | Med |  |  | Max | | |  |  |  |  | | |  | | |
|  | 13.5 | Min |  |  |  | Med |  |  | Max | | |  |  |  |  | | |  | | |
|  | 14.5 |  |  |  |  | Min |  |  | Med | | Max |  |  |  |  | | |  | | |
|  | 15.5 |  |  |  | Min |  |  |  | Med | | |  | Max |  |  | | |  | | |
|  | 16.5 |  |  |  |  |  |  | Min |  | | | Med | Max |  |  | | |  | | |
|  | 17.5 |  |  |  |  |  | Min |  |  | | | Med |  | Max |  | | |  | | |
|  | 18.5 |  |  |  |  |  | Min |  |  | | |  | Med | Max |  | | |  | | |
|  | 19.5 |  |  |  |  |  |  |  | Min | | |  |  | Med | Max | | |  | | |
|  | 20.5 |  |  |  |  |  |  |  |  | | | Min |  |  | Med | | Max |  | | |
|  | 21.5 |  |  |  |  |  |  |  |  | | |  |  | Min | Med | | | Max | | |
|  | 22.5 |  |  |  |  |  |  |  |  | | |  |  | Min | Med | | | Max | | |
|  | 23.5 |  |  |  |  |  |  |  |  | | |  |  |  |  | | | Min | Med | Max |
| * Midpoint of one year | | | | | | | | | | | | | | | | | | | | |

To set up a growth curve for tooth dentine we first must estimate relative dentine amounts of formation stages. In stages Ci, Cco and Coc primarily tooth enamel is produced, which is why they are not considered here. Even if a thin layer of cells is already producing first microscopical layers of dentine, those layers are not analyzed separately but included in the first 1 mm broad dentine section. Their contribution to the isotopic signal of first crown section is assessed as negligible small. Due to that we define Coc as the starting point of dentine formation (dentine amount=0). Crc is the last stage of crown formation. In the following stage Ri already first layers of root dentine are formed thus Crc can also be seen as the starting point of root formation. Dentine formation is nearly ending with stage Rc since maximal root length is completed. The last dentine section includes some dentine formed for Apex closure, but which is assumed to contribute only minor to the isotope signal. Especially for root sections determined age spans are not that precise anyway because sectioning is performed horizontally but dentine is formed rather diagonally, which means that sections include parts of different dentine layers and overlapping developmental stages. But relative differences between sections are assessed to remain true.

We estimate the relative amount of dentine for required stages using pictures and descriptions in AlQahtani et al. [18]. We assume a relatively constant dentine ratio of 1:2 for crown:root and we have eight stages with dentine formation. To calculate with larger numbers and to have similar scales for x (relative dentine amount) and y axis (age in years), we distributed nine parts of dentine (3 for crown and 6 for the root) on these stages (Tab S2.2.2.3).

*Table S2.2.2.3: Estimated relative dentine amount of formation stages*

| Formation stage | Estimated dentine amount |
| --- | --- |
| Coc | 0 |
| Cr½ | 1 |
| Cr¾ | 2 |
| Crc | 3 |
| Ri | 3.5 |
| R¼ | 4.5 |
| R½ | 6 |
| R¾ | 7.5 |
| Rc | 9 |

For nearly all considered age spans the minimal, median and maximal observed stage is given (see Tab S2.2.2.2a–c), but not all formation stages represent the minimal, median and maximal stage of a specific age span. These gaps would make growing curves less accurate especially when information of the starting or end point of dentine formation (Coc, Rc) are missing. Therefore, we estimated chronological ages of all formation stages for an early development (Max), normal development (Med) and late development (Min) for each molar (Tab S2.2.2.4a–b). Some ages are directly readable from tables (e.g., stage Ci of mandibular M1 matches age 0.375 years for normal development (Med)). If there are more than one Max, Med or Min for single stages, we used the mean values. Other ages have to be calculated, as the mean of latest stage before and earliest stage after (e.g., age of stage Cco of mandibular M1 is the mean of 0.375 years (=(latest) Med of stage Ci) and 0.625 years (=earliest Med of stage Coc) (=0.5 years).

*Table S2.2.2.4a: Chronological age of formation stages for an early development, normal development, and late development of the first, second and third mandibular molar*

|  |  | Formation stage (relative dentine amount) | | | | | | | | |
| --- | --- | --- | --- | --- | --- | --- | --- | --- | --- | --- |
| Mandibula | Development | Coc (0) | Cr½ (1) | Cr¾ (2) | Crc (3) | Ri (3,5) | R¼ (4,5) | R½ (6) | R¾ (7,5) | Rc (9) |
| M1 | Early (Max) | 0.75 | 1.5 | 2 | 2.5 | 3.5 | 4 | 5 | 6.5 | 7 |
|  | Normal (Med) | 1 | 2 | 2.5 | 3 | 3.5 | 5 | 6.5 | 8 | 9 |
|  | Late (Min) | 2 | 3 | 4 | 4 | 4 | 5.5 | 7 | 8.5 | 10.5 |
| M2 | Early (Max) | 3.5 | 4.5 | 5.5 | 6.5 | 7 | 8 | 10 | 11.5 | 12.5 |
|  | Normal (Med) | 4.5 | 6 | 7.5 | 8 | 8.5 | 9.5 | 11 | 13 | 14.5 |
|  | Late (Min) | 6 | 7.5 | 8.5 | 9 | 9.5 | 11.5 | 13.5 | 14 | 15 |
| M3 | Early (Max) | 9.5 | 10.5 | 9.5 | 10.5 | 10.5 | 13 | 15 | 16 | 18 |
|  | Normal (Med) | 11.5 | 13 | 14 | 14 | 14 | 15 | 17 | 18.5 | 19.5 |
|  | Late (Min) | 14 | 15 | 16 | 16.5 | 17 | 18.5 | 20 | 20.5 | 22 |

*Table S2.2.2.4b: Chronological age of formation stages for an early development, normal development, and late development of the first, second and third maxillary molar*

|  |  | Formation stage (relative dentine amount) | | | | | | | | |
| --- | --- | --- | --- | --- | --- | --- | --- | --- | --- | --- |
| Maxilla | Development | Coc  (0) | Cr½ (1) | Cr¾ (2) | Crc (3) | Ri (3.5) | R¼ (4.5) | R½ (6) | R¾ (7.5) | Rc (9) |
| M1 | Early (Max) | 0.75 | 1.5 | 2.5 | 3 | 3 | 3.5 | 5 | 6.5 | 7 |
|  | Normal (Med) | 1.1875 | 2 | 2.5 | 3.5 | 4 | 5 | 6.5 | 7.5 | 8.5 |
|  | Late (Min) | 1.6875 | 3 | 4 | 4 | 4 | 6 | 8.5 | 9 | 10 |
| M2 | Early (Max) | 3.5 | 4.5 | 5.5 | 6.5 | 7 | 8 | 10 | 11 | 12 |
|  | Normal (Med) | 4.5 | 6 | 7.5 | 8 | 8.5 | 9.5 | 11 | 13 | 14.5 |
|  | Late (Min) | 8.5 | 7.5 | 8 | 8.5 | 9.5 | 11.5 | 13.5 | 13.5 | 15 |
| M3 | Early (Max) | 8.5 | 10.5 | 9.5 | 10.5 | 11.5 | 13.5 | 15 | 16 | 18 |
|  | Normal (Med) | 10 | 11.5 | 13 | 14 | 14 | 15 | 17 | 18.5 | 19.5 |
|  | Late (Min) | 14 | 15.5 | 14.5 | 18 | 16.5 | 19.5 | 20.5 | 21 | 22 |

When using the estimated dentine amount and chronological age of formation stages, we can display dentine formation as the following functions (Fig S2.2.2.1). The linear trend lines are used to describe the chronological formation of molars (Tab S2.2.2.5). Differences between normal and early development as well as normal and late development represent the error due to biological variation.

One could also consider crown and root dentine formation separately. But we perform cutting on macroscopic level when no enamel is left, which is why we cannot ensure a separation of sections at the direct limit of crown and root dentine and formulars of crown and root dentine taken together are more suitable to determine age spans of dentine sections.


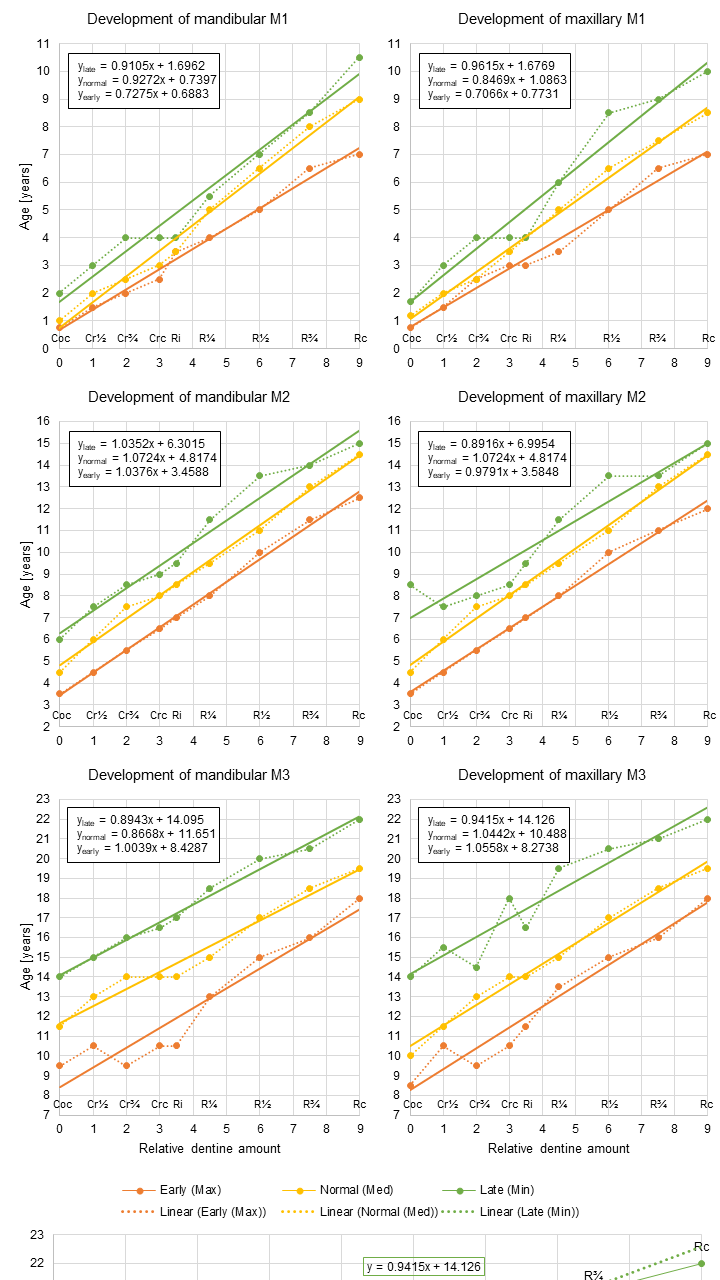


*Figure S2.2.2.1: Dentine growth lines and linear approximations for an early, normal, and late development of mandibular (left) and maxillary molars (right).*

*Table S2.2.2.5: Linear approximation of molar dentine development. x is the relative dentine amount.*

| Jaw part | Molar | Early development | Normal development | Late development |
| --- | --- | --- | --- | --- |
| Maxilla | M1 | 0.7066x+0.7731 | 0.8469x+1.0863 | 0,9615x+1.6769 |
|  | M2 | 0.9791x+3.5848 | 1.0724x+4.8174 | 0,8916x+6.9954 |
|  | M3 | 1.0558x+8.2738 | 1.0442x+10.488 | 0,9415x+14.126 |
| Mandibula | M1 | 0.7275x+0.6883 | 0.9272x+0.7397 | 0,9105x+1.6962 |
|  | M2 | 1.0376x+3.4588 | 1.0724x+4.8174 | 1,0352x+6.3015 |
|  | M3 | 1.0039x+8.4287 | 0.8668x+11.651 | 0,8943x+14.095 |

Each 1 mm dentine section represents a time span no time point. The isotopic signal measured in a section represents a mixing of dietary resources that were consumed during that time, thus a mean value. To calculate the mean of this time span we use the midpoint of relative dentine amount of the section. Crown sections represent 3 parts of absolute tooth dentine (d_c_), if no or only minimal tooth abrasion is present. If a tooth is showing abrasion affecting dentine amount (min stage 3) we must estimate a loss of dentine in absolute dentine (Tab S2.2.2.6)

*Table S2.2.2.6: Estimated loss of dentine amount due to tooth abrasion.*

| Stadium | Description | Absolute crown dentine amount (d_c_) |
| --- | --- | --- |
| 1-2 | Unused surface or small facets (no dentin visible yet) or tooth cusps blunted, dentin may be slightly exposed at tips. | 3 |
| 3 | Tooth cusp completely worn away, some dentin exposed. | 2.75 |
| 4 | Several larger dentin exposures, which have not yet connected, however. | 2.5 |
| 5 | Two sites where dentin is exposed have connected. | 2.25 |
| 6 | Three or four sites where dentin is exposed are connected. | 2 |
| 7 | Dentin exposed on the entire occlusal surface, but enamel at the margin still intact or almost intact. | 1.75 |
| 8 | Severe loss of tooth crown height, enamel margin no longer completely intact, tooth crown surface similar to tooth root surface. | 1.5 |

Midpoints of relative dentine amount of crown sections can be calculated according to the formula:

((d_c_/c)*(n-1)+(d_c_/c)*n)/2

d_c_ = absolute crown dentine amount
c = total number of crown sections
n = number of crown section

Root sections represent 6 parts of absolute tooth dentine (d_r_). Midpoints of relative dentine amount of root sections can be calculated according to the formula:

3+((d_r_/r)*(m-1)+(d_r_/r)*m)/2

d_r_ = absolute root dentine amount
r = total number of root sections
m = number of root section (starting with 1)

Midpoints of relative dentine amounts and the equation for a normal development can be used to estimate the approximate chronological age in which the dentine was formed that matches the measured isotopic signal. Negative and positive error are calculated as the difference between the values using the equation for an early development and normal development or the equation for a late development and normal formation.

## S2.3 Determining the local range of bioavailable strontium

### S2.3.1 The geology of Southern Bavaria

The archaeological sites studied are found in the North Alpine foreland Molasse basin located north of the Alps and south of the Danube (Fig 1).

The geological basement in the North alpine foreland of Bavaria is almost completely covered by younger deposits (e.g., [21]). During the emerge of the Alps, an elongated depression was formed there, which accumulated all the mountain debris: fine sediments, sand, and gravel. Later the interaction of this sediment supply, the basin subsidence and sea-level fluctuation led to shifts between a marine (“Marine Molasse”) and a continental environment (“Freshwater Molasse”) in two great cycles, forming an upper and a lower molasse layer.

The south and west of the molasse basin is characterized by traces of glaciers and their adjacent gravel terraces. During the time of the four major glacial stages (Günz, Mindel, Riss and Würm) in the Quaternary, debris-bearing lacier moved from the Alps into the foreland depositing moraines and gravel with their melt waters. This led to the formation of the so-called *Munich Gravel Plain* during the last glacial, which consists of glaciofluvial deposits overlaying the Neogene sediments of the Upper Freshwater Molasse. Furthermore, fine material of periglacial areas, poor in vegetation, was blown out and redeposited as aeolinan sand or loess. The fertile Gäu-landscape (surrounding Straubing) along the Danube is characterized by such calcareous loess Also, river valleys are often accompanied by terrace complexes composed of gravel and sand which originated from increased sediment supply of glacial melting water.

Adjacent to the foreland basin, to the northwest, lays the cuesta region, revealing increasingly older sections of the Mesozoic era. The landscape of the north-eastern and eastern neighboring region is mainly formed by crystalline basement rocks. To the south of the region lie the Alps which were formed by Mesozoic to Tertiary rocks that were folded and piled up by tectonic activities [21].

Overall, our study area has a fairly uniform, relatively young geological surface and is surrounded by regions that have an, at least partly, different geology e.g., with exposed older rocks.

### S2.3.2 Range of bioavailable strontium isotopes

The range of bioavailable strontium is usually defined through inferring a “local” isotopic range from control data such as geological data, or strontium values of modern plants, archaeological faunal teeth or human bones, or a combination of it (e.g., [22-24]). Another approach is to determine the “local” range from human enamel without the need of proxy information (e.g., [25-26]).

In terms of archaeological strontium isotopy, the Bavaria north Alpine region is one of the best-studied areas in the world. There is now a sample set of 940 strontium ratios for this area available (Fig S2.3.2.1) which can serve as a reference data set for the characterization of the local isotopic ranges for the sites investigated here. In addition to data from previously published studies the reference sample set also contains data from the archaeological sites that are the focus of this study which are published for the first time (S1.2 Table). The data set consists of strontium data from human and animal bones and teeth from adults and children as well as from animals. For further analysis, human and animal bones are grouped together because it can be assumed for both, that their strontium signal represents an unknown mixture of the local burial ground signature and the original signal of the individual, due to diagenetic overprinting). The samples collected in this reference sample set come from 74 archaeological sites ranging from Neolithic to Medieval times (see S2.3.4 for literature references).


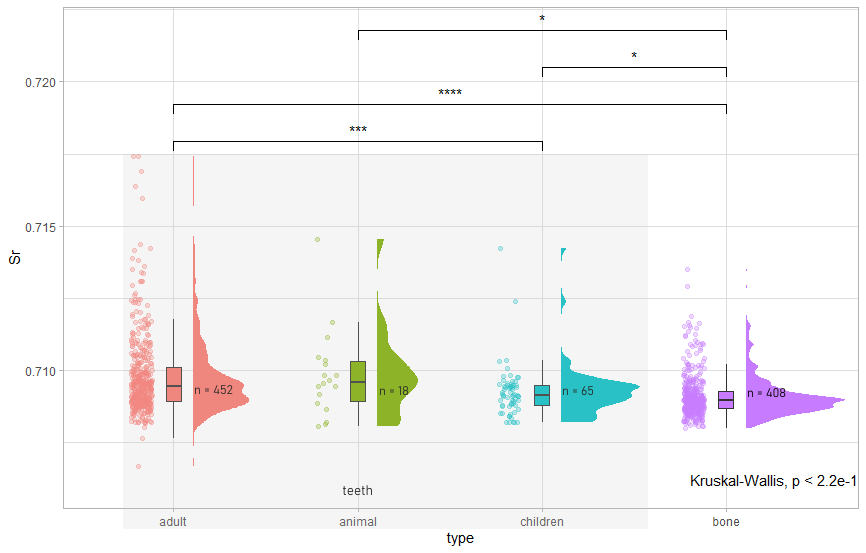


**Fig S2.3.2.1: Reference data grouped by origin (human adult, children and animal teeth and bones)**

Distribution per group shown as points, boxplot, and kernel density estimation. Significant differences between groups are indicated by stars, (* P ≤ 0.05, ** P ≤ 0.01, *** P ≤ 0.001 **** P ≤ 0.0001) (calculation/illustration: R packages ggplot2 [27-28]/ggpubr [29]/ggdist [30]/gghalves [31]).

When using this data set as a reference for the local span of bioavailable strontium, it has to be considered that it almost certainly contains a number of immigrants and thus also non-local signals. This becomes clear in the distribution of the data according to the sampled skeletal element (Fig S2.3.2.1): While bones are more likely to reflect the local ratio, either because they are (at least partially) diagenetically overprinted by the local soil signal or because the individual signal of later life is preserved, teeth are more likely to show a non-local ratio. Teeth are not remodeled and almost not diagenetically altered and therefore show the childhood signal of the individual under study. This may be more likely to deviate from the local bioavailable span than the bone signal in immigrants. Therefore, bones should show less variation than teeth of adult individuals. This also applies to children's teeth, as there is a lower probability that they have already changed location in their few years of life compared to adults (although this cannot be ruled out, of course). However, it does not necessarily apply to animal teeth, as import may have taken place here as well. But the probability of a non-local signal is arguably lower than a change of location of adult humans, depending on the animal species. This should also explain the observed significant group differences in the reference data set (Fig S2.3.2.1).

Nevertheless, it can be assumed, that the majority of individuals are likely to reflect local ratios, while statistical outliers are indicative of immigrant individuals. Therefore, to get a better indication of the of bioavailable strontium for the entire Bavarian Northern Alpine region, outliers were removed. For this purpose, the first and third quartile (Q1=0.70877, Q3=0.70967) as well as the interquartile range (IQR=0.00091) of the whole reference data set were determined. Outliers are defined as samples whose ratios are found outside the range of Q1–1.5*IQR and Q3+1.5*IQR. The resulting dataset of samples with ratios inside the range of 0.70741 to 0.71103 (n=865) is called “adjusted reference dataset”. It is also used to determine the bandwidth for Kernel density determinations (S2.3.3 Text).

The resulting Kernel Density diagram (Fig S2.3.2.2) suggests that the distribution of the adjusted reference dataset is not a purely unimodal distribution, as might be expected from a population of a rather uniform geological background.


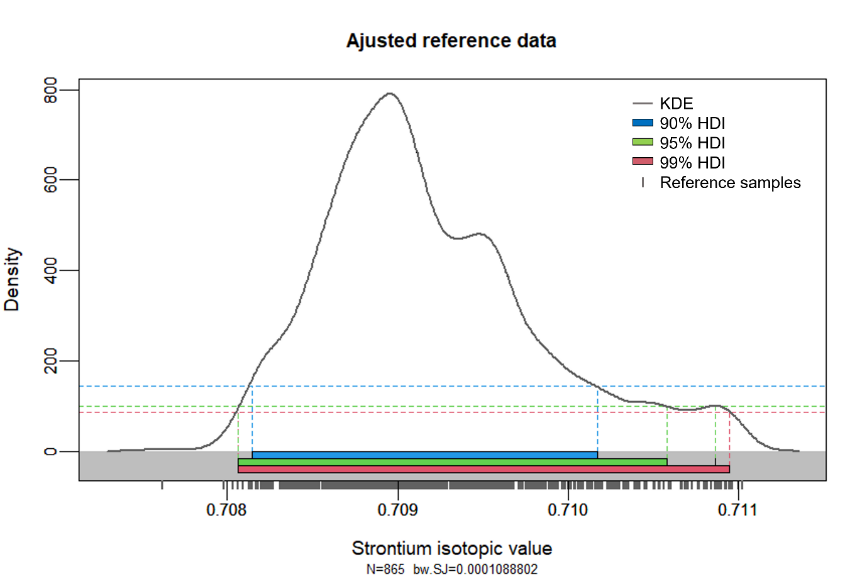


**Fig S2.3.2.2: Kernel Density Plot of the adjusted reference dataset.**

KDE is based on Gaussian kernel. Bandwith for Gaussian kernel is selected using the "solve-the-equation" method of Sheather & Jones [32] (calculation/illustration: R packages hdrcde [33]/ggplot2 [27-28]).

One reason for this is, that not all immigrants were statistically removed. In addition, geographic variations might play a role. It can be seen in Fig S2.3.1 that within the Bavarian Northern Alpine Foreland the average strontium ratios are varying slightly depending on the region. In general, the median values of particular sites in the reference sample set range between 0.70986 and 0.70970. But there seem to be regions with more decreased average ratios than those observed in most parts of the area between Alps and Danube - one of them being the geological unit of the *Munich Gravel Plain* around Munich. It is also noticeable that above-average ratios are only found in sites with less than 10 samples examined, so that it seems rather likely that these are statistical artefacts. Generally, the distribution of ratios within the region should not be overestimated because the geographic distribution of sites is unbalanced, and some sites have a bigger impact because more individuals could be samples there. Hence any existing regional variation of an area with fewer samples can be overlaid by other regions comprising many samples. However, since geographically differing strontium variation inside the Northern Alpine foreland cannot be excluded, it seems reasonable to define local ranges for the study regions Straubing, Regensburg, Erding and Munich (S2.3.3 Text).

Nevertheless, calculating the highest density intervals of the whole area, that span 90, 95 and 99% of the distribution, can indicate probable limits for the bioavailable strontium (Table S2.3.2.1). The HDI of a distribution indicates which points of a distribution are most credible, and which cover most of the distribution. Thus, the HDI summarizes the distribution by specifying an interval that spans most of the distribution, such that every point inside the interval has higher credibility than any point outside the interval [34]. The HDI, which covers 90% of the distribution, seems to be the most suitable for setting the cut offs, since the upper tail, which probably contains subintervals caused by non-local signals, is not included (Fig S2.3.2.2). However, if one wants to set absolute limits that conservatively encompass all possible variation, the 99% HDI seems more appropriate, but there is a greater risk of encompassing a range of non-local signals.

Table S2.3.2.1: Cut off values of the Highest Density Intervals of the adjusted reference data set.

| HDI | Cut offs | |
| --- | --- | --- |
|  | Lower | Upper |
| 99% | 0.70806 | 0.71095 |
| 95% | 0.70806 | 0.71058 |
| 90% | 0.70815 | 0.71017 |

### S2.3.3 Local strontium ranges

With the exception of individuals from the archaeological site Unterhaching the Early Medieval strontium ratios produced in this study fall well within the assumed local range of bioavailable strontium for South Bavaria (Fig S2.3.3.1). Therefore, it can be assumed that the main part of them is “local”. This allows the use of the variation of the respective adult teeth sample sets of the particular sites to further narrow down the local range for each of the sites investigated.


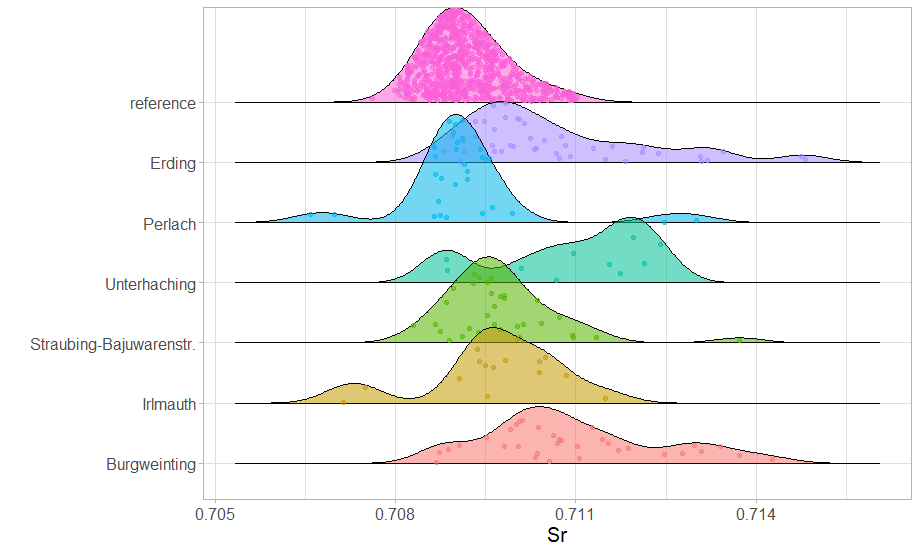


**Fig S2.3.3.1: Distributions of strontium isotope ratios of juvenile and adult teeth**

Ratios of burials from particular archaeological sites are compared with each other and with the adjusted reference sample set (KDE – bandwidth = 0.000413) (calculation/illustration: R packages ggplot2 [27-28]/ggridges [35]).

Therefore, archaeological sites that are found within a 10 km radius of each other were grouped together. In addition, those samples of the adjusted reference data set (human and animal bone and tooth samples from archaeological sites dating from Neolithic to Medieval times) who fall into this radius as well as data from bone samples of our sample set were added. This procedure results in four groups shown in Table S2.3.3.1, for each of which local ratios are to be determined.

Table S2.3.3.1: Groups in study regions (for references see Chapter S2.3.4)

| Sample set  including infants I/II  N=enamel samples | Archaeological sites | Reference sites (within 10 km radius)  human bone and enamel as well as animal bone and enamel samples from reference sites (within 10 km radius) and study sites | N  = sample set+reference data |
| --- | --- | --- | --- |
| Munich (MUC)  N=39 | Unterhaching (UTH), Munich-Perlach (PEL) | Grünwald, München Trudering, München Engelschalking, Unterhaching am Rodelberg, Unterhaching Urnfield culture (all from Toncala et al. 2017), Unterhaching Early Medieval (Harbeck et al. 2013), | 76 |
| Erding (AED)  N=45 | Altenerding (AED) | Erding Late Antique (Sofeso et al. 2012), Erding Prehistory (Toncala et al. 2017), Langengeisling (Toncala et al. 2017), Altenerding Early Medieval (S2.1 Table) | 78 |
| Straubing  N=31 | Straubing-Bajuwarenstr. (STB) | Aiterhofen-Ödmühle (Bentley et al. 2012), Straubing-Öberau (Price et al. 2004), Straubing Azlburg (Schweissing 2005), Alburg Medieval (S2.2 Table), Alburg-Lerchenhaid (Bertemes & Heyd 2015), Alburg Bell Beaker (Sjögren et al. 2020), Straubing-Bajuwarenstraße Early Medieval (S2.1 Table, S2.2 Table) | 217 |
| Regensburg  N=47 | Irlmauth (IRM), Burgweinting A+B (BWA, BWB),  Alteglofsheim (AEH) | Mintraching LBK (Bentley & Knipper 2005), Mintraching Corded Ware (Sjörgren et al. 2016), Regensburg BW Roman (Codreanu-Windauer & Harbeck 2016), Trothengasse (Harbeck et al. 2018), Ostengasse (Harbeck et al. 2018), Minoritenweg (von Heyking 2012, Harbeck et al. 2018), Irlmauth Early Medieval (S2.1 Table, S2.2 Table), Alteglofsheim (S2.1 Table), Regensburg BW Early Medieval (Codreanu-Windauer & Harbeck 2016) | 80 |

Kernel-Density-Plots and HDIs are calculated using the hdr.den function of R package hdrcde with predefined bandwidths [33] and shown in Fig S2.3.3.2a–d. Optimal bandwidth is selected using the "solve-the-equation" method of Sheather & Jones [32] and relies on the adjusted reference dataset: 0.0001088802.

For the regions of Munich, Erding and Straubing the determination of the main peak seems quite clear, whereas for Regensburg the situation appears more complex. As the Regensburg region contains the most geological variation and is close to areas with other isotope ratios, this does not seem to be far-fetched, but it makes the estimation of the cut offs less reliable than for the other areas (see also discussion in Codreanu-Windauer&Harbeck [36]).

However, also in Erding, Munich and Straubing indications are present that there may be several sub-distributions. But in general, it would be difficult to decide whether these are based on migration processes or are an expression of geological variation, food preferences or likewise. Furthermore, it would be impossible to decide which of the main distributions reflects “local” conditions with the available data. Therefore, the main part of the distribution, even if it appears quite clearly bimodal as in the case of Straubing, is considered as a whole to determine the local ratio. But the situation is different for the lower modes in upper and lower tails of the distribution, which again can clearly be interpreted as an indication of a non-local population share (see Chapter S2.3.2). To exclude them, an HDI of 90% seems appropriate to determine the local range of bioavailable Strontium, but contrary involve the risk that isolated local individuals are wrongly classified as migrants. Therefore, also HDIs are given that cover 95% or 99% of the data (Table S2.3.3.2). Using 99% HDIs ensures that we are not overestimating but rather underestimating the number of migrants.


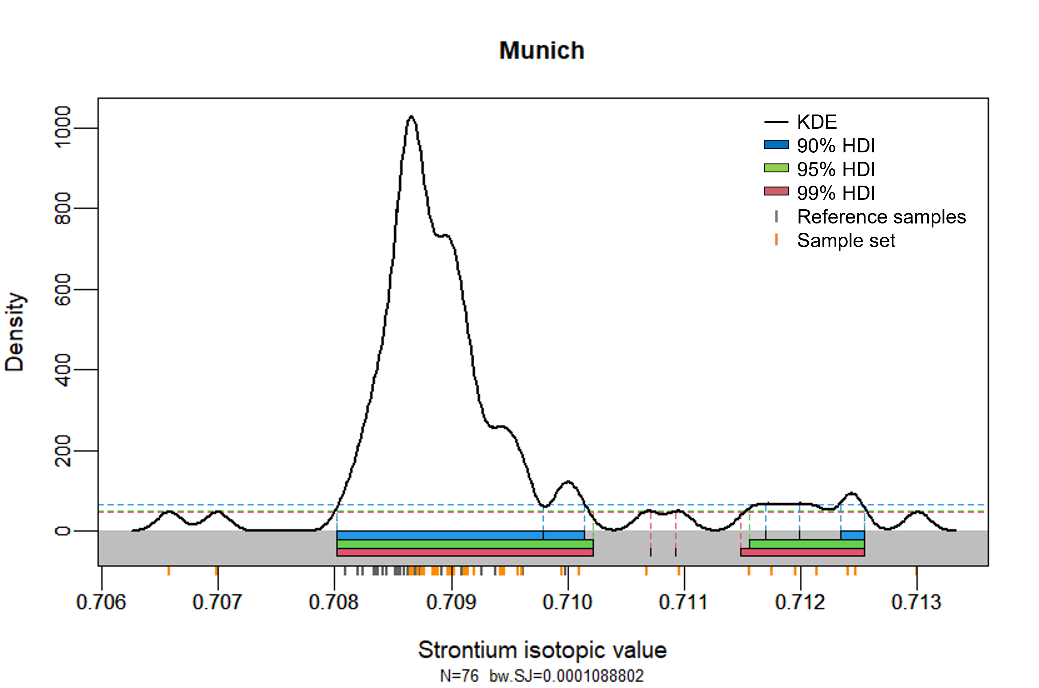


**Fig S2.3.3.2a: Kernel Density Plot of ^87^Sr/^86^Sr at Munich.**

KDE is based on Gaussian kernel. Bandwidth for Gaussian kernel is the bandwidth of the adjusted reference sample set selected using the "solve-the-equation" method of Sheather & Jones [32] (calculation/illustration: R packages hdrcde [33]/gglpot2 [27-28]).


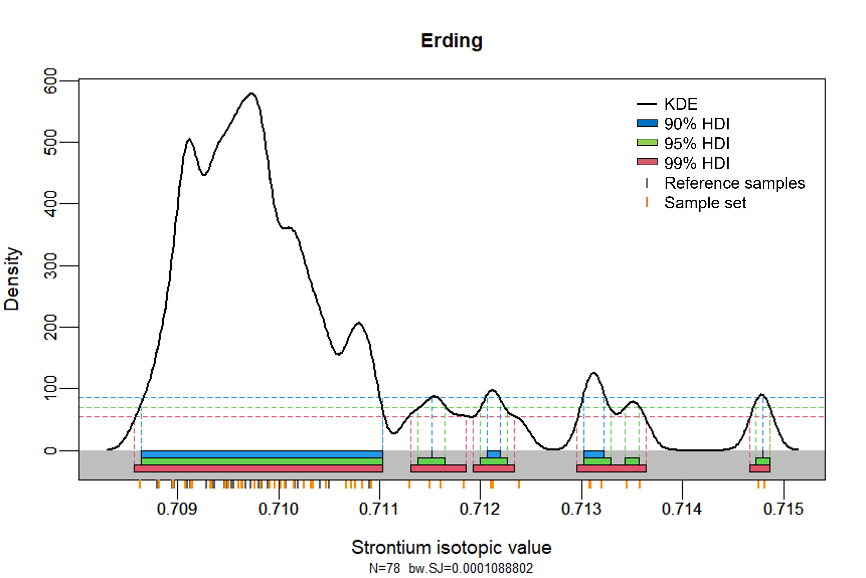


**Fig S2.3.3.2b: Kernel Density Plot at ^87^Sr/^86^Sr at Erding.**

KDE is based on Gaussian kernel. Bandwidth for Gaussian kernel is the bandwidth of the adjusted reference sample set selected using the "solve-the-equation" method of Sheather & Jones [32] (calculation/illustration: R packages hdrcde [33]/gglpot2 [27-28]).


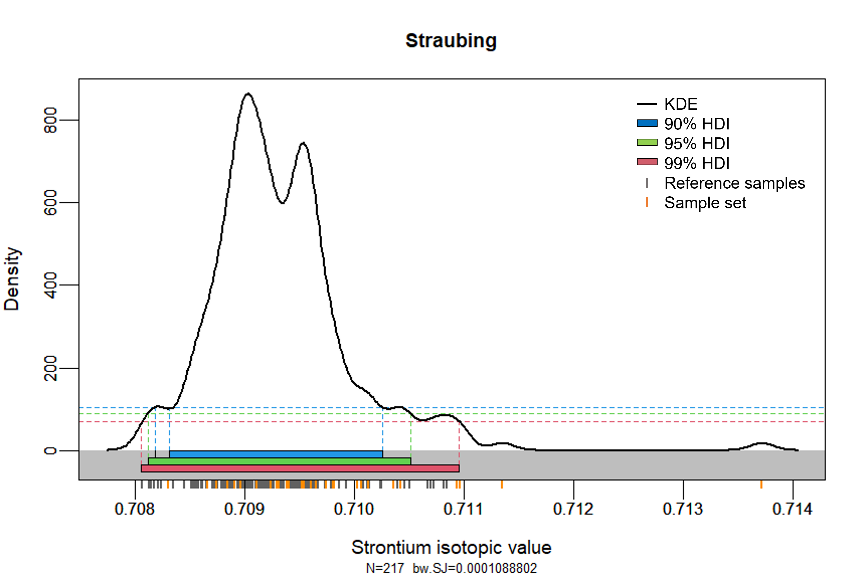


**Fig S2.3.3.2c: Kernel Density Plot at ^87^Sr/^86^Sr in Straubing**.

KDE is based on Gaussian kernel. Bandwidth for Gaussian kernel is the bandwidth of the adjusted reference sample set selected using the "solve-the-equation" method of Sheather & Jones [32] (calculation/illustration: R packages hdrcde [33]/gglpot2 [27-28]).


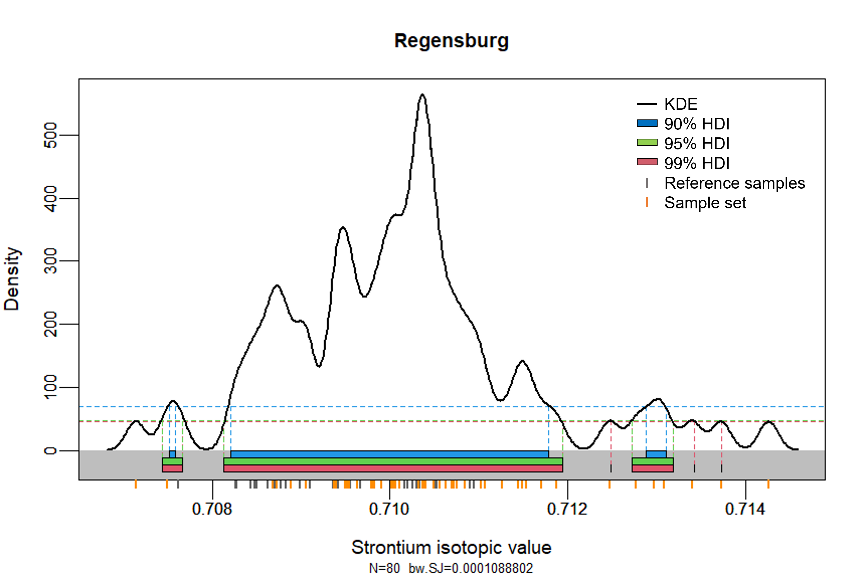


**Fig S2.3.3.2d: Kernel Density Plot at ^87^Sr/^86^Sr in Regensburg.**

KDE is based on Gaussian kernel. Bandwidth for Gaussian kernel is the bandwidth of the adjusted reference sample set selected using the "solve-the-equation" method of Sheather & Jones [32] (calculation/illustration: R packages hdrcde [33]/gglpot2 [27-28]).

Table S2.3.3.2: HDI of study regions (STB=Straubing, REG=Regensburg, AED=Erding, MUC=München).

| Group | 90% HDI | 95% HDI | 99% HDI |
| --- | --- | --- | --- |
| STB | 0.70831 – 0.71026 | 0.70812 – 0.71051 | 0.70805 – 0.71096 |
| REG | 0.70821 – 0.71179 | 0.70813 – 0.71195 | 0.70813 – 0.71195 |
| AED | 0.70864 – 0.71104 | 0.70864 – 0.71104 | 0.70857 – 0.71104 |
| MUC | 0.70802 – 0.70979 | 0.70802 – 0.71021 | 0.70802 – 0.71021 |

Enamel samples with ^87^Sr/^86^Sr ratios outside 99% HDI identify related individuals as non-locals. Individuals showing ^87^Sr/^86^Sr ratios between 90% and 99% HDIs are recognized as potential migrants (Table S2.3.3.3).

Table S2.3.3.3: Non-locals and potential migrants found at the study regions identified by stable strontium analysis. (STB=Straubing, REG=Regensburg, AED=Erding, MUC=München).

| Zone | Region | Non-locals | | Potential migrants | |
| --- | --- | --- | --- | --- | --- |
|  |  | ^87^Sr/^86^Sr > 99% HDI | ^87^Sr/^86^Sr  < 99% HDI | ^87^Sr/^86^Sr > 90% HDI | ^87^Sr/^86^Sr  < 90% HDI |
| Border | STB | STB_300, STB_395 | - | STB_316, STB_317, STB_360, STB_377 | STB_600 |
|  | REG | BWB_3734, BWB_3735, BWB_3739, BWB_3740, BWB_3741, BWA_10077, BWA_10253 | IRM_20, IRM_21 | BWB_3743 |  |
| Hinterland | AED | AED_94, AED_160, AED_201, AED_211, AED_321, AED_343, AED_344, AED_487, AED_492, AED_501, AED_521, AED_1123, AED_1135, AED_1143 | - | - | AED_1129 |
|  | MUC | PEL_22, PEL_27, UTH_1, UTH_2, UTH_6, UTH_7, UTH_8, UTH_9, UTH_10 | PEL_12, PEL_19 | PEL_7, UTH_4 | - |
|  | N=164 | N=34 | N=2 | N=7 | N=2 |

### S2.3.4 Literature for reference data set

A few strontium ratios of the reference data set are published here for the first time and can be found in S1.2 Table. Most strontium ratios of the reference data, including human and animal bone and teeth samples from more than 70 archaeological sites ranging from Neolithic to Medieval times, were taken from the following publications:

Alt KW, Oelze VM, Lahaye Y, Weix J, Dörr W, Klein S. Identifizierung von Einheimischen und Ortsfremden in der Mehrfachbestattung Grab 244 aus Ergolding anhand standorttypischer Strontiumisotopie. In: Koch H, editor. Frühmittelalterliche Adelsgräber aus Ergolding. Büchenbach: Verlag Dr. Faustus. 2014; pp. 70-73.

Bentley RA, Bickle P, Fibiger L, Nowell GM, Dale CW, Hedges REM, et al. Community differentiation and kinship among Europe’s first farmers. PNAS. 2012; 109(24): 9326-9330.

Bentley RA, Price TD, Lüning J, Gronenborn D, Wahl J, Fullagar PD. Prehistoric Migration in Europe: Strontium Isotope Analysis of Early Neolithic Skeletons. Curr Anthropol. 2002 Dec; 43(5):799-804.

Bentley RA, Knipper C. Geographical patterns in biologically available strontium, carbon and oxygen isotope signatures in prehistoric SW Germany. Archaeometry 2005; 47:629-644.

Bertemes F, Heyd V. 2200 BC – Innovation or Evolution? The genesis of the Danubian Early Bronze Age. In: Meller H, Risch R, Jung R, Arz HW, editors. 2200 BC – A climatic breakdown as a cause for the collapse of the old world? 7th Archaeological Conference of Central Germany October 23-26, 2014 in Halle (Saale), Tagungen des Landesmuseums für Vorgeschichte Halle 12. Halle/Saale: Landesamt für Denkmalpflege und Archäologie Sachsen-Anhalt. Landesmuseum für Vorgeschichte. 2014; 561-578.

Bickle P, Hofmann D, Bentley RA, Hedges R, Hamilton J, Laiginhas F, et al. Roots of diversity in a Linearbandkeramik community: isotope evidence at Aiterhofen (Bavaria, Germany). Antiquity. 2011 Nov; 85(330):1243-1258.

Codreanu-Windauer S, Harbeck M. Neue Untersuchungen zu Gräbern des 5. Jahrhunderts: Der Fall Burgweinting - In: Geisler H, editor. Wandel durch Migration? Büchenbach: Dr. Faustus. 2016; 243-260.

Grupe G, Price TD, Schröter P., Söllner F., Johnson CM, Beard BL. Mobility of Bell Beaker people reveald by stronium isotpe ratios of tooth and bone: a study of southern Bavarian skeletal remains. Appl Geochem. 1997 Jul; 12:517-525.

Harbeck M, Codreanu-Windauer S, McGlynn G, Müller R, and Haberstroh J. Living at the outskirts of the Roman Empire after the fall. A study of 5th century Bavarian burials. Interdiscip Archaeol. 2016; 7(1):123-135.

Harbeck M, Zäuner S, von Heyking K. Anthropologische Spurensuche im "Melting pot" Regensburg: Bestimmung der lokalen Strontiumisotopenwerte und Analyse der Skelettfunde der Trothen- und Ostengasse. Fines Transire. 2018; 27:179-195.

von Heyking K. Anthropologie einer frühstädtischen Randgruppe morphologische und archäometrische Untersuchung eines hoch- bis spätmittelalterlichen Armenhausgräberfeldes in Regensburg. Ludwig-Maximilians-Universität. 2012; 302 p.

Knipper C, Mittnik A, Massy K, Kociumaka C, Kucukkalipci I, Maus M, et al. Female exogamy and gene pool diversification at the transition from the Final Neolithic to the Early Bronze Age in central Europe. PNAS. 2017 Sep 19;114(38):10083-10088.

Neumann D, Pütz A, Vohberger M. Ein schnurkeramisches Grab mit Silexdolchbeigabe aus Aschheim (Lkr. München). Absolute Datierung, Stronitumisotopenanalysen und archäologische Vergleiche. Archäologisches Korrespondenzblatt. 2015; 45:37-51.

Price TD, Knipper C, Grupe G, Smrcka V. Strontium isotopes and prehistoric human migration: The Bell Beaker period in central Europe. Eur J Archaeol. 2004; 7:9-40.

Sofeso C, Vohberger M, Wisnowsky A, Päffgen B, Harbeck M. Verifying archaeological hypotheses: Investigations on origin and genealogical lineages of a privileged society in Upper Bavaria from Imperial Roman times (Erding, Kletthamer Feld). In: Burger J, Kaiser E, Schier W, editors. Population dynamics in pre- and Early History. New Approaches by using Stable Isotopes and Genetics. Berlin: De Gruyter. 2012; 115-132.

Schweissing M. Archäometrische Analyse spätantiker Gräber aus Bayern. In: Moosbauer G, editor. Kastell und Friedhöfe der Spätantike in Straubing. Passauer Universitäts-Schriften zur Archäologie. Passau: Marie-Leidorf. 2005; 249-295.

Schweissing MM, Grupe G. Local or nonlocal? A research of strontium isotope ratios of teeth and bones on skeletal remains with artificial deformed skulls. Anthropol Anz. 2000 Mar; 58(1):99-103.

Schweissing M. Ergebnisse der Strontiumisotopenanalyse (^87^Sr/^86^Sr) an Zähnen aus Manching. In: Sievers S, Leicht M, Ziegaus B, editors. Ergebnisse der Ausgrabungen in Manching-Altenfeld 1996-1999. Wiesbaden: Reichert. 2013; 705-707.

Schweissing M, Grupe G. Stable strontium isotopes in human teeth and bone: a key to migration events of the late Roman period in Bavaria. J Archaeol Sci. 2003; 30:1373-1383.

Strott N, Czermak A, Grupe G. Are biological correlates to social stratification depicted in skeletal finds? Investigation of early medieval separate burial grounds in Bavaria. Documenta Archaeobiologiae. 2008; 5:68-86.

Sjögren KG, Olalde I, Carver S, Allentoft ME, Knowles T, Kroonen G, et al. Kinship and social organization in Copper Age Europe. A cross-disciplinary analysis of archaeology, DNA, isotopes, and anthropology from two Bell Beaker cemeteries. PLoS ONE. 2020 Nov 16; 15(11):e0241278.

Sjögren KG, Price TD, Kristiansen K. Diet and Mobility in the Corded Ware of Central Europe. PLoS ONE. 2016 May 25;11(5):e0155083.

Toncala A, Söllner F, Mayr C, Hölzl S, Heck K, Wycisk D, et al. Isotopic Map of the Inn-Eisack-Adige-Brenner Passage and its Application to Prehistoric Human Cremations. In: Grupe G, Grigat A, McGlynn GC, editors. Across the Alps in Prehistory. Cham: Springer International Publishing. 2017; 127-227.

Toncala A, Trautmann B, Velte M, Kropf E, McGlynn G, Peters J, et al. On the premises of mixing models to define local bioavailable ^87^Sr/^86^Sr ranges in archaeological contexts. Sci Total Environ. 2020 Nov; 745:1-13.

## S2.4 Determining the “common intra-populational variability” of carbon and nitrogen from the diet

We use KDEs and HDIs to describe the distribution of δ^13^C and δ^15^N which display the “common variability” of diet in a population. In contrast to the procedure in strontium isotope analysis no reference data is used. Information about human basic diet in Bavaria results from comparison with faunal samples (S3.1 Text). Observed differences in variability and position of stable light isotopes in human bone collagen between study sites indicate that common variability of diet may differ due to some ecogeographic factors or varying subsistence strategies. However, data of really close (<5 km) sites (Munich-Perlach and Unterhaching, as well as Burgweinting, Irlmauth and Alteglofsheim) show great overlap (S3.1.2 Fig) and presumably had a similar catchment area [37-38], why we combine data of these sites which also increases sample sizes. Samples of infans I children (n=11) were excluded from analysis to eliminate the effect of breastfeeding, which could lead to unusual isotopic ratios.

Table S2.4.1: Grouping of δ^13^C and δ^15^N data.

| Number of bone samples | Archaeological sites |
| --- | --- |
| 29 | Unterhaching (UTH), Munich-Perlach (PEL) |
| 44 | Altenerding (AED) |
| 33 | Straubing-Bajuwarenstr. (STB) |
| 49 | Irlmauth (IRM), Burgweinting A+B (BWA, BWB),  Alteglofsheim (AEH) |

Bandwidth for Gaussian Kernel density estimations is based on all human bone collagen samples (N=155) independent from site according to the “solve the equation” method by Sheather & Jones [32]: 0.2398928 for δ^15^N and 0.2046038 for δ^13^C.

Fig S2.4.1a–d and Fig S2.4.2a–d show KDE plots of nitrogen and carbon ratios at study regions including 90, 95 and 99% HDIs.


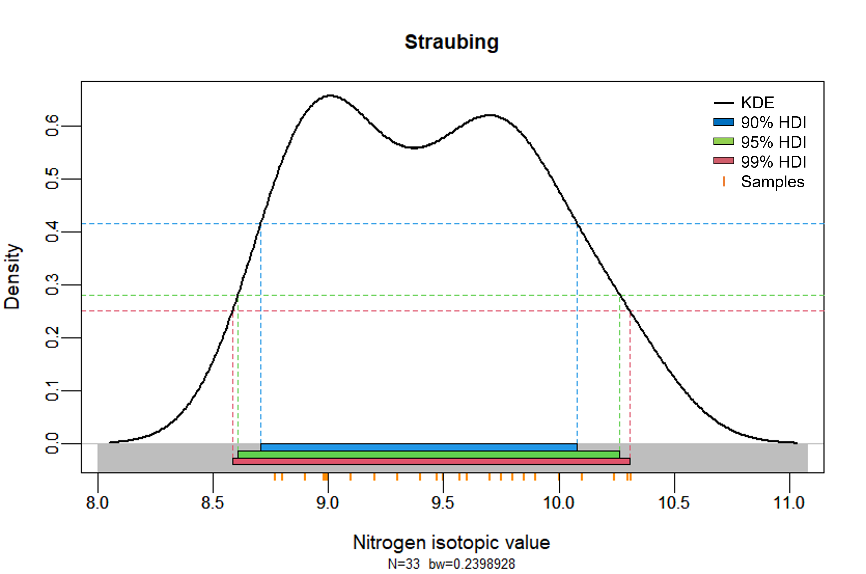


**Fig S2.4.1a: Kernel Density Plot of δ^15^N of Straubing-Bajuwarenstraße.**

KDE is based on Gaussian kernel. Bandwidth for Gaussian kernel based on all human bone collagen samples independent from site using the "solve-the-equation" method of Sheather & Jones [32] (calculation/illustration: R packages hdrcde [33]/gglpot2 [27-28]).


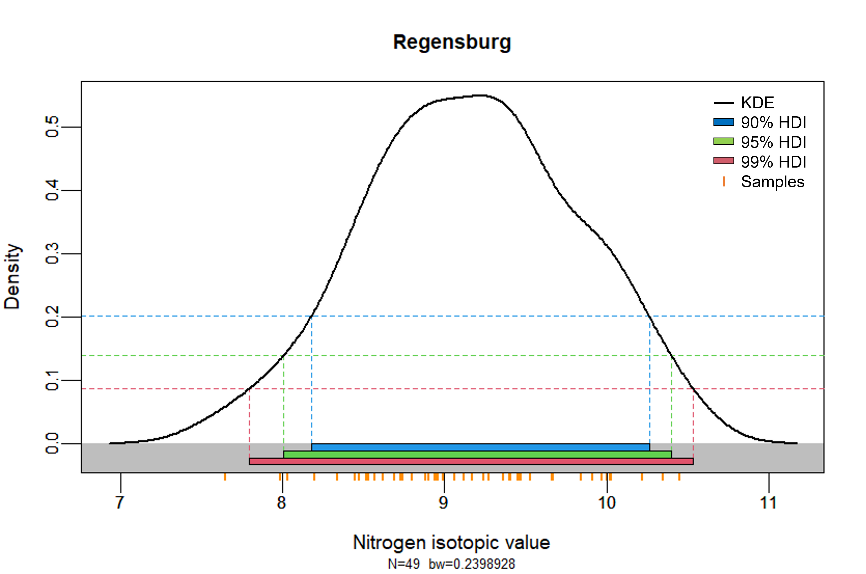


**Fig S2.4.1b: Kernel Density Plot of δ^15^N of Burgweinting A+B, Irlmauth and Alteglofsheim.**

KDE is based on Gaussian kernel. Bandwidth for Gaussian kernel based on all human bone collagen samples independent from site using the "solve-the-equation" method of Sheather & Jones [32] (calculation/illustration: R packages hdrcde [33]/gglpot2 [27-28]).


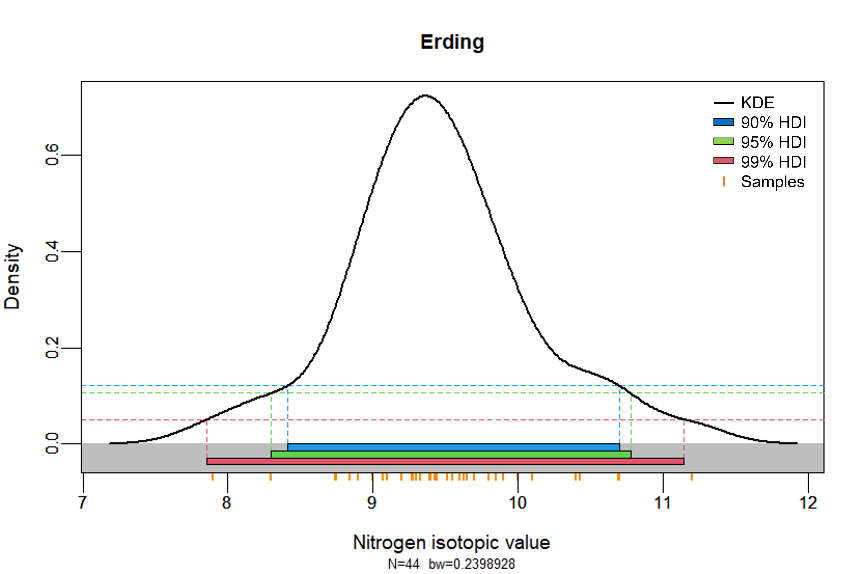


**Fig S2.4.1c: Kernel Density Plot of δ^15^N of Altenerding.**

KDE is based on Gaussian kernel. Bandwidth for Gaussian kernel based on all human bone collagen samples independent from site using the "solve-the-equation" method of Sheather & [32] (calculation/illustration: R packages hdrcde [33]/gglpot2 [27-28]).


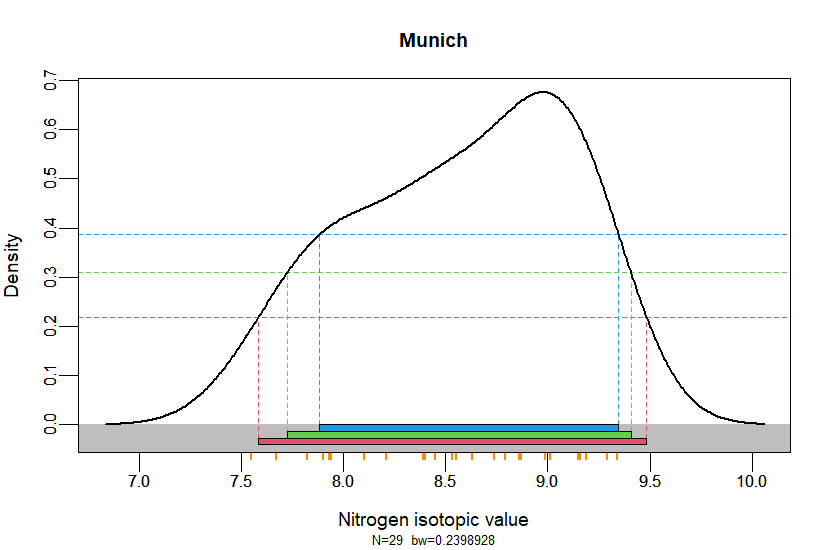


**Fig S2.4.1d Kernel Density Plot of δ^15^N of Munich-Perlach and Unterhaching**.

KDE is based on Gaussian kernel. Bandwidth for Gaussian kernel based on all human bone collagen samples independent from site using the "solve-the-equation" method of Sheather & Jones [[32] (calculation/illustration: R packages hdrcde [33]/gglpot2 [27-28]).


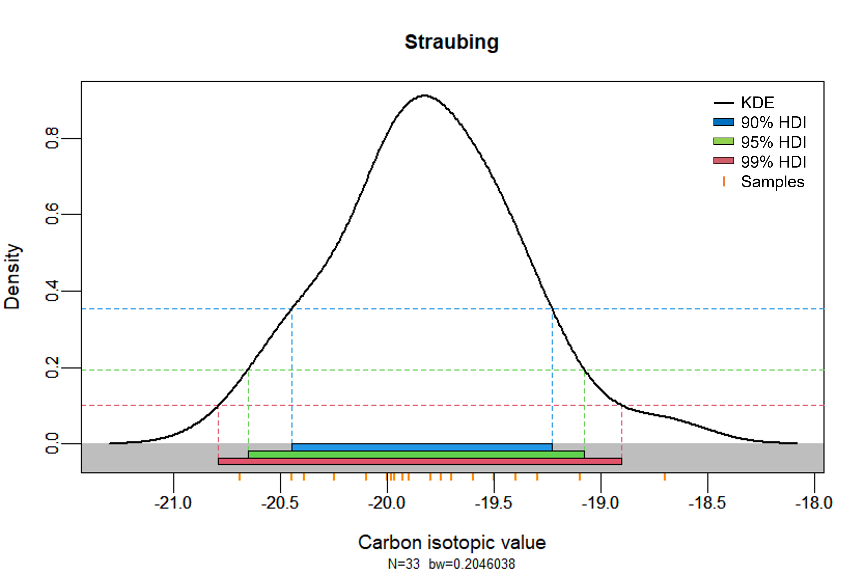


**Fig S2.4.2a: Kernel Density Plot of δ^13^C of Straubing-Bajuwarenstraße**.

KDE is based on Gaussian kernel. Bandwidth for Gaussian kernel based on all human bone collagen samples independent from site using the "solve-the-equation" method of Sheather & Jones [32] (calculation/illustration: R packages hdrcde [33]/gglpot2 [27-28]).


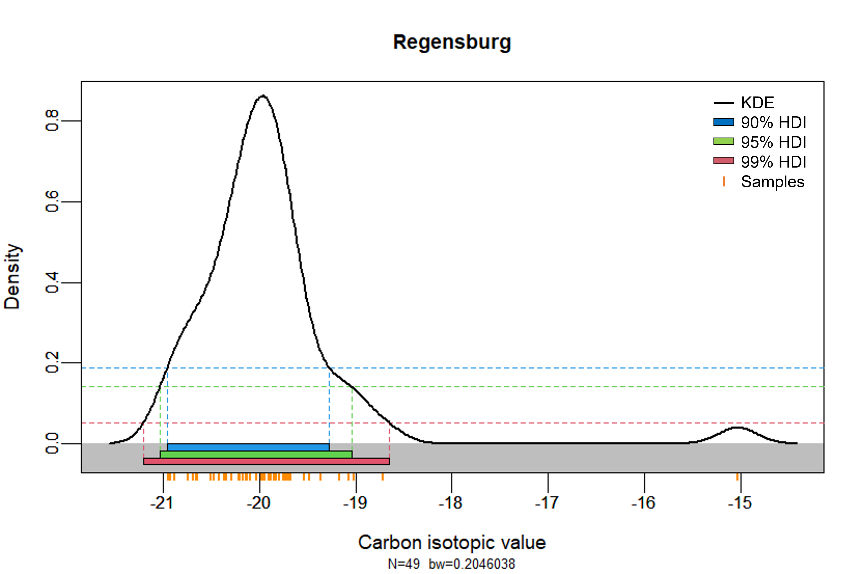


**Fig S2.4.2b: Kernel Density Plot of δ^13^C of Burgweinting A+B, Irlmauth and Alteglofsheim**.

KDE is based on Gaussian kernel. Bandwidth for Gaussian kernel based on all human bone collagen samples independent from site using the "solve-the-equation" method of Sheather & Jones [32] (calculation/illustration: R packages hdrcde [33]/gglpot2 [27-28]).


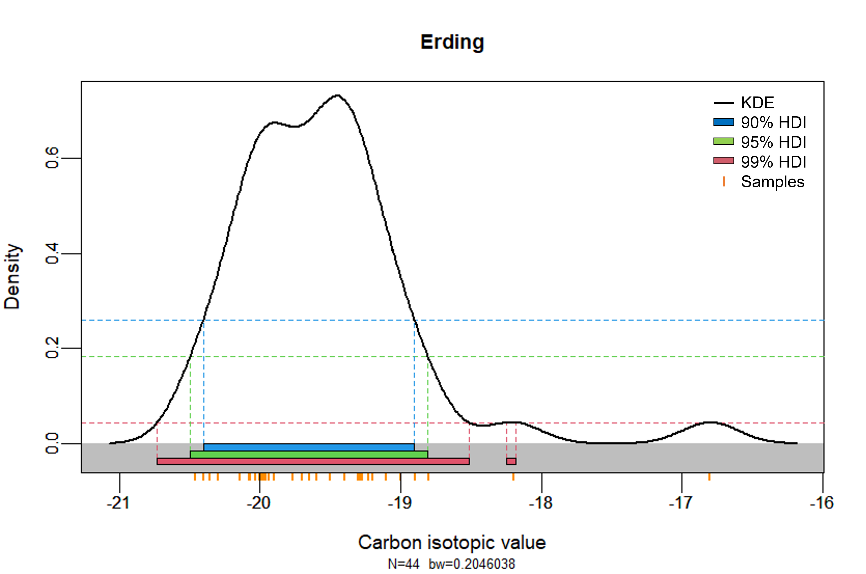


**Fig S2.4.2c: Kernel Density Plot of δ^13^C of Altenerding**.

KDE is based on Gaussian kernel. Bandwidth for Gaussian kernel based on all human bone collagen samples independent from site using the "solve-the-equation" method of Sheather & Jones [32] (calculation/illustration: R packages hdrcde [33]/gglpot2 [27-28]).


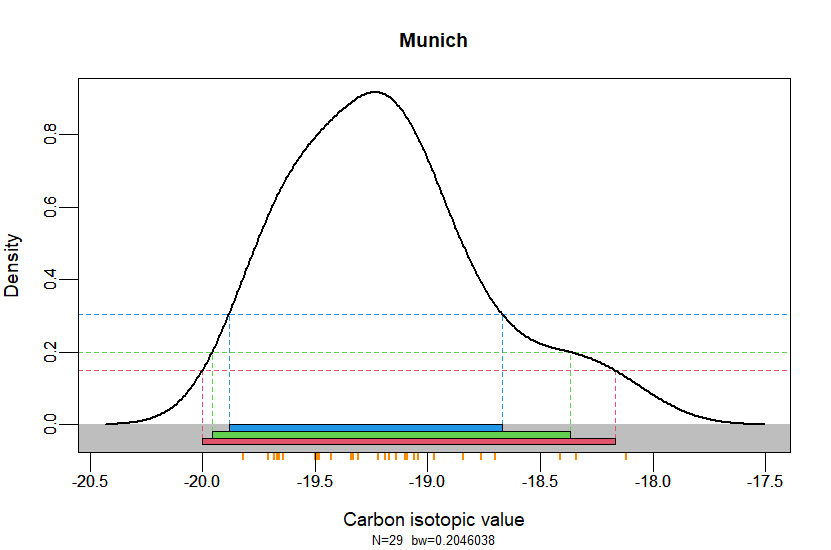


**Fig S2.4.2d: Kernel Density Plot of δ^13^C of Munich-Perlach and Unterhaching.**

KDE is based on Gaussian kernel. Bandwidth for Gaussian kernel based on all human bone collagen samples independent from site using the "solve-the-equation" method of Sheather & Jones [[32] (calculation/illustration: R packages hdrcde [33]/gglpot2 [27-28]).

Table S2.4.2: Ranges of “common variability” of diet in population groups (study sites: STB=Straubing-Bajuwarenstraße, BW=Burgweinting, IRM=Irlmauth, AEH=Alteglofsheim, AED=Altenerding, PEL=Munich-Perlach, UTH=Unterhaching).

| Group | δ^13^C [‰] | | | δ^15^N [‰] | | |
| --- | --- | --- | --- | --- | --- | --- |
|  | 99% HDI | 95% HDI | 90% HDI | 99% HDI | 95% HDI | 90% HDI |
| STB (N=33) | -20.8 to -18.9 | -20.7 to -19.1 | -20.5 to -19.2 | 8.6 to 10.3 | 8.6 to 10.3 | 8.7 to 10.1 |
| BW+IRM+AEH (N=49) | -21.2 to -18.7 | -21.0 to -19.0 | -21.0 to -19.3 | 7.8 to 10.5 | 8.0 to 10.4 | 8.2 to 10.3 |
| AED (N=44) | -20.7 to -18.5 | -20.5 to -18.8 | -20.4 to -18.9 | 7.9 to 11.1 | 8.3 to 10.8 | 8.4 to 10.7 |
| PEL+UTH (N=29) | -20.0 to -18.2 | -20.0 to -18.4 | -19.9 to -18.7 | 7.6 to 9.5 | 7.7 to 9.4 | 7.9 to 9.4 |

Table S2.4.3: Individuals with deviating carbon and or nitrogen ratios (study sites: STB=Straubing-Bajuwarenstraße, BW=Burgweinting, IRM=Irlmauth, AEH=Alteglofsheim, AED=Altenerding, PEL=Munich-Perlach, UTH=Unterhaching). ↑ increased ratios, ↓ decreased ratios, red=infans I children, orange=infans II child.

| Zone | Group | 99% HDI | | | | 90% HDI | | | |
| --- | --- | --- | --- | --- | --- | --- | --- | --- | --- |
|  |  | δ^13^C ↑ | δ^15^N ↑ | δ^13^C ↓ | δ^15^N ↓ | δ^13^C ↑ | δ^15^N ↑ | δ^13^C ↓ | δ^15^N ↓ |
| Border | STB | STB_228* | - | - | - | STB_310 | STB_220, STB_326, STB_330, STB_395 | STB_521 | - |
|  | BW+IRM+AEH | BWA_10071 | BWB_3888 |  | BWA_10071 | IRM_22, BWB_3743, BWA_10076, BWA_10078, BWA_10249 | IRM_24, IRM_26, IRM_36b |  | IRM_23, BWA_10079 |
| Hinterland | AED | AED_513*, AED_1108* | AED_421 | AED_1098 | - | AED_521, AED_552 | - | AED_1138 | AED_343, AED_280, AED_289 |
|  | PEL+UTH | PEL_18 | PEL_21 |  | PEL_11, PEL_28 | PEL_7, PEL_26 |  |  | PEL_24, PEL_29 |
|  | N=166 | N=5 | N=3 | N=1 | N=3 | N=10 | N=7 | N=2 | N=7 |
|  |  | N=11 | | | | N=26 | | | |

Looking at the bone bulk ratios of individuals (Fig 4), nitrogen or carbon bone ratios of seven infans I children (IRM_24, BWA_10076, BWB_3888, AED _552, AED_1098, PEL_11, PEL_21) can be found outside 90% or 99% HDI ranges (Table S2.4.3). This corresponds to 64% of all infans I children investigated. Therefore, it can be safely assumed that the deviating ratios of infans I children result from dietary patterns like breastfeeding or metabolic features and are not associated with migration. The decreased nitrogen ratio of one infans II child (PEL_28) is also not necessarily indicating a non-local diet but could refer to an observed drop of nitrogen ratios in younger age (S3.1 Text).

As ecogeographic conditions are rather similar (terrestrial, C3-plant environment) we expect no remarkable differences between different populations in southern Bavaria. However, unusual food sources such as C4-plants or marine fish, consumed in larger quantities and for extended periods, are assumed to be uncommon and might be an indicator of a non-local origin.

Six individuals are found outside 99% HDIs what probably indicates the usage of non-local resources:

A senile woman from Altenerding (AED_421) shows a significantly increased nitrogen ratio, what may be explained by significant amounts of marine fish in diet at some point in her life (as discussed by Hakenbeck et al. [2]) or resources from a terrestrial ecosystem with a higher nitrogen baseline. Only higher amounts of animal protein are unlikely to explain the observed increase in δ^15^N.

The other five females (STB_228*, AED_513*, AED_1108*, BWA_10071, PEL_18) show increased δ^13^C values that most likely display the consumption of a higher amount of millet. One female with a C4 plant signal (BWA_10071) has a simultaneously decreased nitrogen ratio, which may refer to a region with a generally lower nitrogen baseline or indicate less animal protein in diet.

23 individuals show δ^13^C or δ^15^N values that are found outside stricter 90% HDIs (Table S2.4.3).

In some cases, possible usage of non-local resources is supposedly supported by PCA (South-East European ancestry: STB_310) or stable strontium isotope analysis (non-local: STB_395, AED_343; potential migrants: BWB_3743, PEL_7). But for most of the individuals with potentially deviating dietary patterns a non-local origin is reasonable but remains questionable. Ranges of stable light isotope ratios of bone collagen that sufficiently describe common intra-populational variability of diet are generally difficult to define, but probably even more in small sample sets. On the one hand, ranges could underestimate the intra-populational variability of diet and some deviating ratios may also display individual dietary patterns rather than the usage of non-local resources in a different habitat. On the other hand, ranges could also overestimate the dietary variability in a population. This is more likely to happen in groups that generally show a wider and fragmentary spread of carbon and nitrogen ratios. Even more of the outer ratios might refer to the usage of resources from other ecosystems and some potential migrants remain undetected. Despite this, remodeling of bone tissue may lead to a mixed signal of local and non-local resources that is hard to interpret and could finally completely overprint non-local signatures.

In contrast to strontium isotopes, no clear identifiable side peaks can be observed within the 99% HDI beside the main peak. Therefore, it seems more reasonable to conservatively assume that only bone collagen outside these ranges indicate usage of non-local resources.

## References

[1] Veeramah KR, Rott A, Groß M, van Dorp L, López S, Kirsanow K, et al. Population genomic analysis of elongated skulls reveals extensive female-biased immigration in Early Medieval Bavaria. PNAS. 2018 Mar 27; 115(13):3494–9.

[2] Hakenbeck SE, McManus E, Geisler H, Grupe G, O’Connell T. Diet and mobility in Early Medieval Bavaria: A study of carbon and nitrogen stable isotopes. Am J Phys Anthropol. 2010 Oct; 143(2):235-249.

[3] Rösch M, Jacomet S, Karg S. The history of cereals in the region of the former Duchy of Swabia (Herzogtum Schwaben) from the Roman to the Post-medieval period: results of archaeobotanical research. Veget Hist Archaebot. 1992 Dec; 1(4): 193-231.

[4] Knipper C, Peters D, Meyer C, Maurer AF, Muhl A, Schöne BR, et al. Dietary reconstruction in Migration Period Central Germany: a carbon and nitrogen isotope study. Archaeol Anthropol Sci. 2013 Mar; 5(1):17-35.

[5] Gyulai F. Historical plant-biodiversity in the Carpathian Basin. In: Jerem E, Mester Z, Benczes R, editors. Archaeological and cultural heritage preservation. Budapest: Archaeolingua. 2006; 63-72.

[6] Gyulai F. The history of broomcorn millet (*Panicum miliaceum L.*) in the Carpathian Basin in the mirror of archaeobotanical remains II. From the Roman age until the late medieval age. Columella - Journal of Agricultural and Environmental Sciences. 2014; 1:39-47.

[7] Iacumin P, Galli E, Cavalli F, Cecere I. C4-consumers in southern Europe: the case of friuli V. G. (NE-Italy) during early and central Middle Ages. Am J Phys Anthropol. 2014; 154(4):561-574.

[8] Miller NF. Rainfall seasonality and the spread of millet cultivation in Eurasia. Iranian Journal of Archaeological Studies. 2015; 5(1):1-10.

[9] Hakenbeck SE, Evans J, Chapman H, Fóthi E. Practising pastoralism in an agricultural environment: An isotopic analysis of the impact of the Hunnic incursions on Pannonian populations. Caramelli D, editor. PLoS ONE. 2017 Mar 22; 12(3):e0173079.

[10] Paladin A, Moghaddam N, Stawinoga A E, Siebke I, Depellegrin V, Tecchiati U, Lösch S, Zink A. Early medieval Italian Alps: reconstructing diet and mobility in the valleys. Archaeol Anthropol Sci. 2020; 12(82):1-20.

[11] Milella M, Caspari G, Laffranchi Z, Arenz G, Sadykov T, Blochin J, Keller M, Kapinus Y, Lösch S. Dining in Tuva: Social correlates of diet and mobility in Southern Siberia during the 2nd and 4th centuries CE. Am J Biol Anthropol. 2022; 178:124-139.

[12] Cocozza C, Cirelli E, Gross M, Teegen WR, Fernandes R. Presenting the Compendium Isotoporum Medii Aevi, a Multi-Isotope Database for Medieval Europe. Nature Scientific Data. 2022; 9:354.

[13] Makarewicz CA, Sealy J. Dietary reconstruction, mobility, and the analysis of ancient skeletal tissues: Expanding the prospects of stable isotope research in archaeology. J Archaeol Sci. 2015 Apr; 56:146-158.

[14] Parfitt AM. Misconceptions (2): turnover is always higher in cancellous than in cortical bone. Bone. 2002; 30(6):807-809.

[15] Ubelaker DH, Parra RC. Radiocarbon analysis of dental enamel and bone to evaluate date of birth and death: perspective from the southern hemosphere. Forensic Sci Int. 2011; 208:103-107.

[16] Calcagnile L, Quarta G, Cattaneo C, D'Elia M. Determining ^14^C content in different human tissues: Implications for application of 14C bomb-spike dating in forensic medicine. Radiocarbon. 2013; 55(2-3):1845-1849.

[17] Ubelaker DH Plens CR Pessoa Soriano E Vitor Diniz M de Almeida Junior E Daruge Junior E Francesquini junior L Palhares Machado CE. Lag time of modern bomb-pulse radiocarbon in human bone tissues: New data from Brazil. Forensic Sci Int. 2022; 331:111143.

[18] AlQahtani SJ, Hector MP, Liversidge HM. Brief communication: The London atlas of human tooth development and eruption. Am J Phys Anthropol. 2010 Mar 22; 142(3):481-490.

[19] Moorrees CF, Fanning EA, Hunt EE Jr. Age variation of formation stages for ten permanent teeth. J Dent Res. 1963; 42:490-502.

[20] Moorrees CF, Fanning EA, Hunt EE Jr. Formation and resorption of three deciduous teeth in children. Am J Phys Anthropol. 1963; 21:205-213.

[21] Glaser S, Doppler G, Schwerd K. GeoBavaria. 600 Millionen Jahre Bayern. Internationale Edition. Bayerisches Geologisches Landesamt. 2004; 92 p.

[22] Montgomery J, Evans JA, Cooper RE. Resolving archaeological populations with Sr-isotope mixing models. Appl Geochem. 2007 Jul; 22(7):1502-1514.

[23] Maurer AF, Galer SJG, Knipper C, Beierlein L, Nunn EV, Peters D, et al. Bioavailable ^87^Sr/^86^Sr in different environmental samples - Effects of anthropogenic contamination and implications for isoscapes in past migration studies. Sci Total Environ. 2012 Sep; 433:216-229.

[24] Toncala A, Trautmann B, Velte M, Kropf E, McGlynn G, Peters J, et al. On the premises of mixing models to define local bioavailable ^87^Sr/^86^Sr ranges in archaeological contexts. Sci Total Environ. 2020 Nov; 745:140902.

[25] Evans JA, Chenery CA, Montgomery J. A summary of strontium and oxygen isotope variation in archaeological human tooth enamel excavated from Britain. J Anal At Spectrom. 2012; 27(5):1-68.

[26] Burton JH, Hahn R. Assessing the “Local” ^87^Sr/^86^Sr Ratio for Humans. In: Grupe G, McGlynn GC, editors. Isotopic Landscapes in Bioarchaeology. Berlin, Heidelberg: Springer Berlin Heidelberg. 2016; 113-121.

[27] Wickham H. ggplot2: Elegant Graphics for Data Analysis. Springer: New York. 2016; 260 p.

[28] Wickham H, Chang W, Henry L, Pedersen TL, Takahashi K, Wilke C, et al. ggplot2: Create Elegant Data Visualisations Using the Grammar of Graphics. R package version 3.4.0. 2022; Available from: https://ggplot2.tidyverse.org.

[29] Kassambara A. ggpubr: 'ggplot2' Based Publication Ready Plots. R package version 0.5.0. 2022; Available from: https://rpkgs.datanovia.com/ggpubr/.

[30] Kay M, Wiernik BM. ggdist: Visualizations of Distributions and Uncertainty. R package version 3.2.0. 2022; Available from: https://mjskay.github.io/ggdist/.

[31] Tiedemann F. gghalves: Compose Half-Half Plots Using Your Favorite Geoms. R package version 0.1.4. 2022; Available from: https://github.com/erocoar/gghalves.

[32] Sheather SJ, Jones MC. A Reliable Data-Based Bandwidth Selection Method for Kernel Density Estimation. J R Stat Soc Series B Stat Methodol. 1991 Jul; 53(3):683-690.

[33] Hyndman RJ, Einbeck J, Wand MP. hdrcde: Highest Density Regions and Conditional Density Estimation*.* R package version 3.4. 2021; Available from: https://pkg.robjhyndman.com/hdrcde/.

[34] Kruschke JK. Doing Bayesian data analysis: a tutorial with R, JAGS, and Stan. Edition 2. Boston: Academic Press. 2015; 759 p.

[35] Wilke CO. Ridgeline Plots in 'ggplot2'. R package version 0.5.4. 2022; Available from: https://wilkelab.org/ggridges/.

[36] Codreanu-Windauer S, Harbeck M. Neue Untersuchungen zu Gräbern des 5. Jahrhunderts: Der Fall Burgweinting - In: Geisler H, editor. Wandel durch Migration? Büchenbach: Dr. Faustus. 2016; 243-260.

[37] Chisholm M. Rural Settlement and Land Use, Hutchinson & Co., London. 1968; 183 p.

[38] Jones G, Bogaard A, Halstead P, Charles M & Smith H. Identifying the intensity of crop husbandry practices on the basis of weed floras. The Annual of the British School at Athens. 1999; 94:167-89.
