# Supplementary material for: Between Raetia Secunda and the dutchy of Bavaria: Exploring patterns of human movement and diet
Source: PLoS One. 2023 Apr 5;18(4):e0283243. doi: 10.1371/journal.pone.0283243 (PMC10075417; doi:10.1371/journal.pone.0283243)
Supplement: S3 Text — (DOCX) [file pone.0283243.s003.docx]

# S3 Statistics and detailed results

## S3.1 Human diet

Stable light isotope ratios of human bones exhibited a substantial scattering (Fig S3.1.1). Except for a few outliers δ^15^N values are consistent with typical human ratios between 8 to 10‰ using terrestrial resources [1-3]. δ^13^C values are in keeping with δ^13^C values around ~-20‰ for humans with a C3 plant based terrestrial diet in a temperate ecosystem [2-4], without single deviating values.

Stable light isotope ratios of human bone collagen in comparison to animal data from seven sites dating from Late Antiquity to Late Medieval times (S1.4 Table, [5-8]) are shown in Fig S3.1.1.


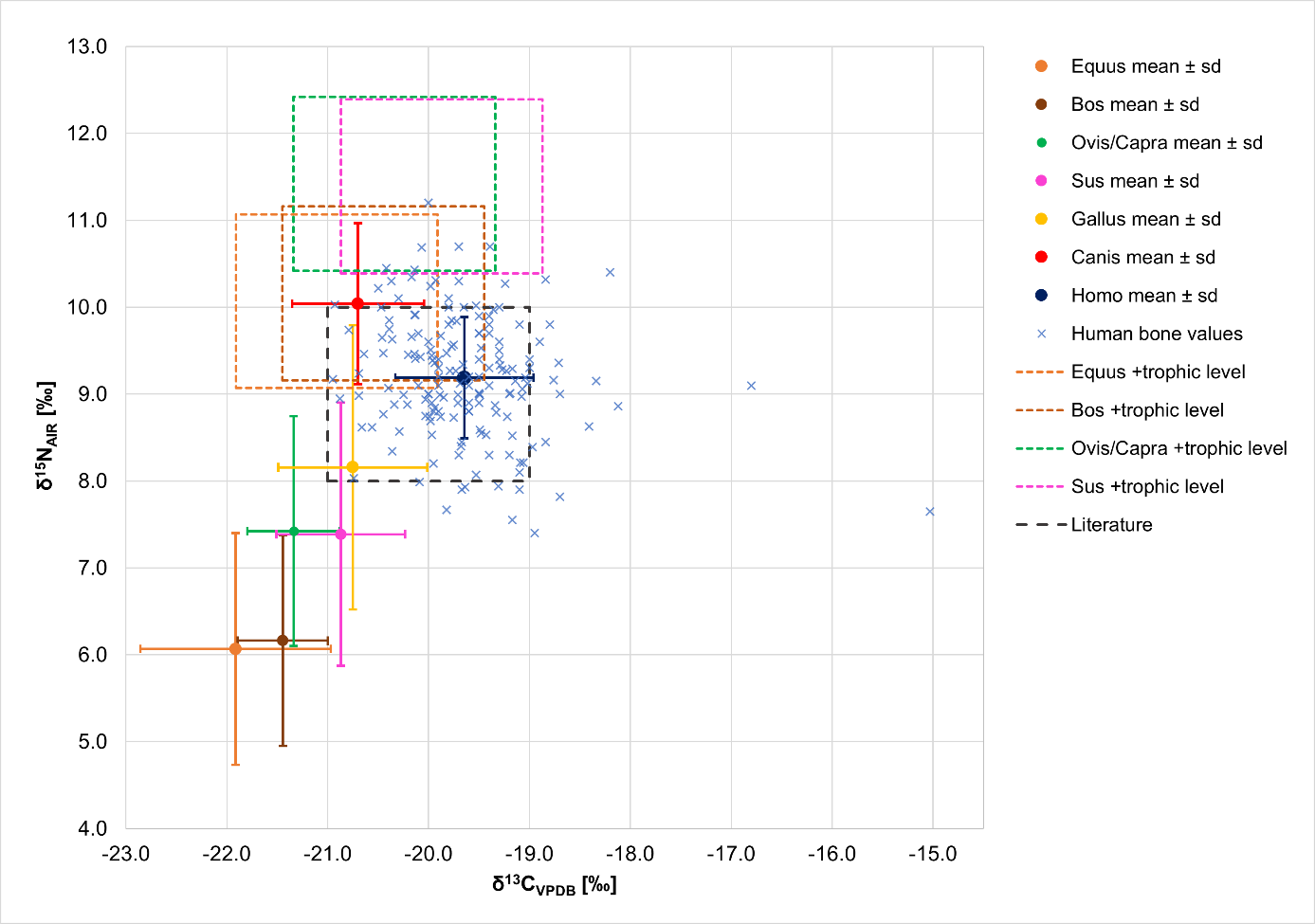


**Fig S3.1.1: Bone collagen ratios of humans**.

Bone collagen mean values ± SD of humans and animal species (min five samples). Dotted rectangles indicate data ranges one trophic level above the domestic horses, cattle, pigs, sheep/goat (mean value of each species plus 0 to 2‰ for δ^13^C [9-10] and 3 to 5‰ to δ^15^N [10-11] as well as values from the literature for C3 based and mainly terrestrial diet (in Central Europe) (illustration: Excel).

δ^15^N values of humans are, on average (9.2‰), increased by 3.1‰ in contrast to herbivores (mean 6.1‰), equal to one trophic level. A process of fractionation also leads to the enrichment of ^15^N from diet to collagen, higher trophic levels therefore have 3.0 to 5.0‰ increased δ^15^N values [10-11]. This and the position between omnivorous birds and omnivorous to carnivorous canids is indicating a large amount of animal protein in human diet. Such a high proportion of meat and dairy products contradicts the general assumption about plant food typically dominating in non-industrialized populations [12-13]. Plants commonly make up more than 85% of their nutritional intake of which cereals alone usually comprise 60 to 85% and the contribution of animal-derived products is low overall [12]. Based on the assumption about Early Medieval diet, of which cereals are assumed to be a major component [14], it seems plausible that human δ^15^N values are influenced by factors other than the proportion of animal protein, even if meat of omnivore animals with increased δ^15^N values are considered. Excessive land use and the practice of fertilization (fecal manure, green manure) in agriculture may enrich ^15^N in crops. The spreading of organic material from manure heaps or composts increased with heavy nitrogen isotopes leads to an increased uptake of ^15^N (in form of nitrate or ammonium) into plant tissues leading to increased δ^15^N values. The Romans already practiced different forms of manuring (e.g., liming, green manure) but to a lesser extent than people in the Middle Ages, in which the “age weakness of the soil” became a serious problem, leading to catastrophic crop failures and famines. Despite this, grains on average inherently reveal 2.4±0.8‰ increased δ^15^N values than the rachis [15]. Humans who primarily consume grains may therefore achieve more increased nitrogen values than animals that may feed on threshing refuse and straw [16]. Another dietary component that may contribute to the elevation of δ^15^N values is fish. Marine organisms generally tend to have increased δ^15^N values than terrestrial ones due to the presence of ^15^N-enriched nitrates in seawater and the often longer marine food chains. The latter also applies for freshwater fish leading to increased δ^15^N values. To estimate the contribution of marine or freshwater fish to human diet one must additionally consider carbon isotopic ratios. Marine resources (δ^13^C of approximately -14.0 to -10.0‰ [1, 17-18]) like sea fish with its increased δ^15^N and δ^13^C in comparison to C3 plants and herbivore meat in a C3-based ecosystem [19-20] would lead to a wider herbivore–human spacing of both isotope ratios. But since all study sites are situated several hundred kilometers away from the ocean coast the access to marine dietary sources should be very limited. This is also supported by decreased sulfur ratios in bone collagen (2.0 to 7.7‰, mean 5.8‰) of 11 individuals (S1.1 Table) from Erding, Munich and Regensburg (e.g., [21]). Freshwater fish shows an enormous carbon range due to large systematic differences in carbon isotope fractionation of phytoplankton and periphyton dependent on water turbidity [22-24] that overlaps with marine fish [24] but also with terrestrial resources. Larger isotopic fractionations discriminating against ^13^C (approaching that of terrestrial C3 plants) occurs during photosynthesis in faster moving waters of riverine ecosystems that can give rise to a food web with especially decreased δ^13^C values [24-26]. The consumption of freshwater fish from riverine ecosystems would likely result in increased δ^15^N (and, but also in decreased δ^13^C values, but the opposite is observed for the latter.

Human bone collagen δ^13^C ratio is on average (-19.7‰), 2.0‰ increased compared to the combined mean of all herbivores (-21.7‰) and 1.7‰ above the mean of cattle (-21.4‰). Both ratios are higher than the typical trophic level enrichment of δ^13^C values of 0.8 to 1.3‰ [27-28] and at the upper limit of a wider, more conservative span of 0 to 2.0‰ [9-10]. A lot of human δ^13^C values plotting less negatively than expected indicate human plant diet to clearly differ from animal forage. Possible explanations for human plant food that has less negative δ^13^C values are a minor contribution of C4 plants (-14.0 to -9.0‰ [29-31], such as millet [5, 32].

Archaeobotanical research at Early Medieval sites in Bavaria showed the isolated occurrence of millet [33]. Also, a study in South-Western Germany indicates that broomcorn and foxtail millet were identified at only 10 and 30% of examined Early Medieval sites [34]. But even the Romans cultivated millet at sites north of the Alps [34-35]. C4 plants like millet are adapted to warm and dry climates. It cannot germinate at temperatures below 10°C and does not tolerate frost, but it grows on nutrient-poor and dry soil and has a rather short growing period (ca. 3 months). Thus, it is possible to grow millet as spring or summer crop in colder regions. Because of its short growth period, millet is particularly suitable as "back-up-crop" in case of low yields from early crops or crop failures. Moreover, once the husks have been removed, millet can be stored for a very long time without any loss of quality, partly twice as long as wheat [36]. The short growing season, high tolerance to soil and climatic conditions (aside from cold temperatures and frost), ease of harvest, and storage stability make millet an ideal crop for small, self-sufficient subsistence farms. Nevertheless, a regularly consumption of millet in higher amounts is not to be assumed from archaeological record and carbon isotopic data. Another explanation for slightly increased carbon signatures is the consumption of photosynthetic inactive parts of C3 plants like roots, seeds, and fruits that enrich ^13^C up to 1.0 to 3.0‰ [37]. Cereals, which are dominating human plant diet, show 1.0 to 2.0‰ increased δ^13^C values in the grains than in the flag leaf, stalk, or chaff [38]. Again, a primary consumption of grains by humans and not by animals could explain the bigger herbivore-human spacing. Furthermore, average δ^13^C values of animal forage may be generally decreased because of probable contributions of plants from more humid or forested habitats [39-40]. Assuming a plant–herbivore collagen isotope shift of +5.0‰ [9, 41], average carbon isotope ratios of the plant forage would be approximately -26.9‰ for horses and -26.4‰ for cattle which is typical for C3 plants (ranging from -35.0 to -20.0‰ [[29-31], [42-43]). Coming back to animal-derived protein in human diet, the consumption of vegetable resources with increased δ^13^C values like C4 plants or C3 cereal grains could also outbalance the more negative δ^13^C signal of freshwater fish consumption [16].

Overall, human data reflect a mixed diet in a temperate C3-plant based ecosystem. This is in keeping with the archaeological evidence for Southern Germany from Late Antique and Early Medieval times which suggests that a wide range of C3 plants (cereal crops, oil and fiber plants, pulses) were cultivated [34-35, 44-45].

The human–faunal isotope spacing exceeds one trophic level in δ^13^C and is in the range of about one trophic level in δ^15^N. Hakenbeck et al. [5], attribute this at least partly to increased protein consumption. But it could also indicate a significant contribution of foodstuff to the human diet that is not directly deducible from the animal collagen data. In accordance with Knipper et al. [16], we suggest these dietary components are most likely cereal grains and other staple crops that grew on arable land under different conditions than the animal forage, possibly including a certain but limited input of millet. Besides that, the amount of animal protein in human basic diet was rather moderate and may also derive from low amounts of freshwater fish next to terrestrial animals. An input of marine resources seems rather unlikely overall.

Fig S3.1.2 shows the variability of stable light isotopes of bone samples within each population at study sites.


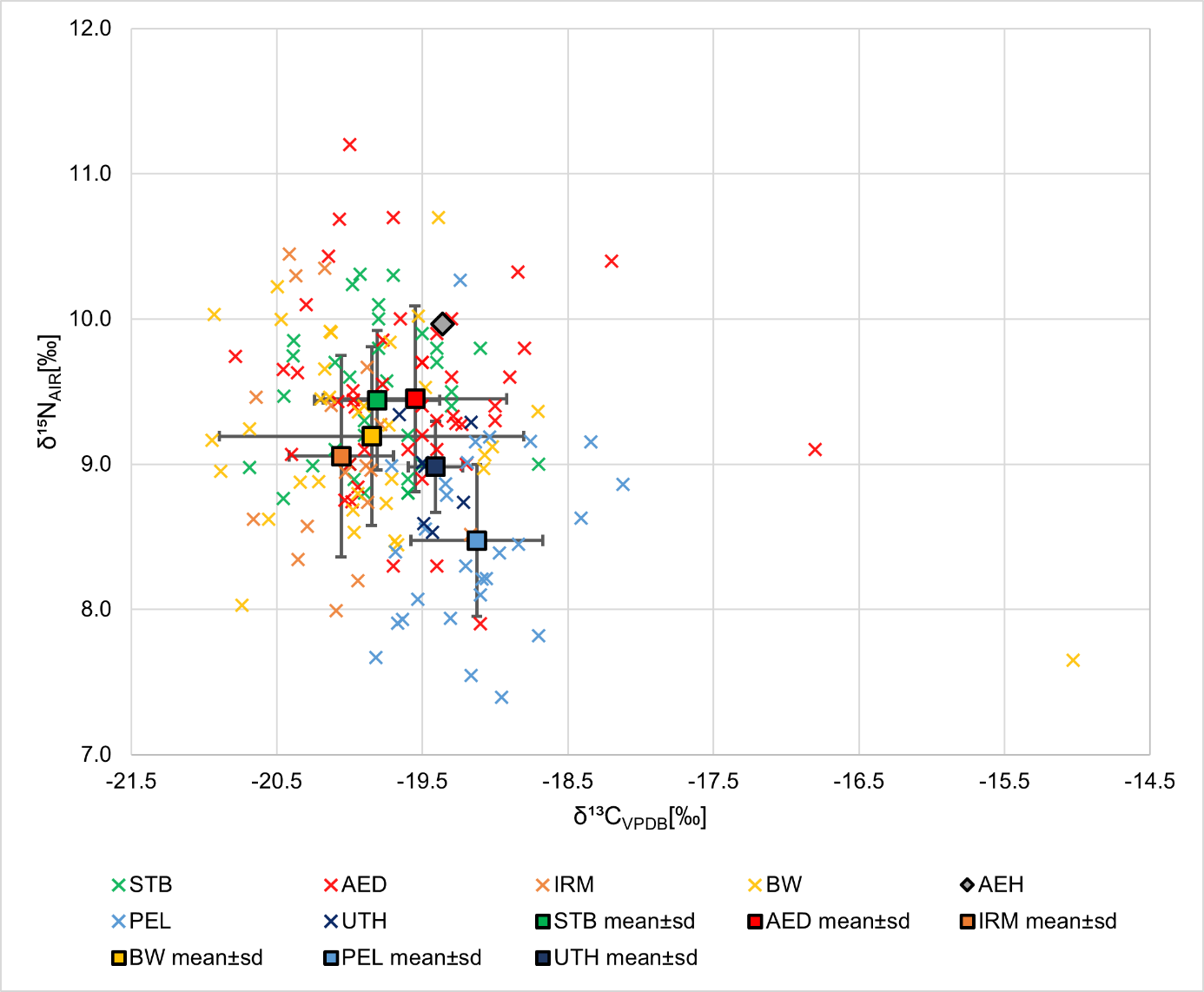


**Fig S3.1.2: Human bone collagen δ^13^C and δ^15^N values in bone collagen of excavation sites.**

Mean values and standard deviations (sd) are calculated excluding infans I children (illustration:Excel).

The scattering of stable light isotopes at all sites suggest that individuals consumed different amounts of plant and animal food sources. Reasons for a higher variability are not entirely clear, but smaller and more continuous clusters might indicate closed communities with similar access to food resources, whereas a wider and patchy scattering of data reveal a rather heterogeneous community, with varying access to certain food resources [46].

Stable light isotope ratios in bone collagen of most non-local individuals and potential migrants, as determined by strontium isotope analysis are not distinct from the spectrum of the “local” populations (Table S3.1.1.3.1). They may come from regions with similar isotopic ranges, which is most probably true for young children, whose bones do not show frequent remodeling and isotopically overprinting yet. Men and women from isotopically distinct regions who reached adult to senile ages may also have lived in Bavaria for years after their arrival and thus recorded the “common” dietary signal. Nevertheless, variability in diet could in part be linked to the mixed composition of communities including individuals of different origin and ethnics [47].

The often-made assumption that men consume more animal protein or higher trophic level protein than women (e.g., [46]) is not supported by our data in general, but δ^15^N and δ^13^C mean values of males tend to be slightly increased than female ratios at most burial sites, except for the small necropolis in Burgweinting (BW) and Unterhaching (UTH) (Table S3.1.1.1.4).

The group of infans I children (0.5-6 years) shows the most variable nitrogen bone ratios. Interestingly, infans II children (7-12 years) and juveniles (13-20 years) tend to have more decreased δ^15^N bone values than older age groups. A drop in nitrogen after the weaning process in later childhood and slightly increasing nitrogen ratios in later youth can also be seen in serial dentine analysis (Fig S3.1.1.2.5).

Remarkable high variability of isotopic ratios in infans I children seems plausible, because some of them are likely to be breastfed (increased ratios) or in the process of weaning (depleting ratios) others are already weaned and have a mixed diet like adults. Moreover, it remains unknown if and how circumstances of early death may influence stable light isotopes measured in bone tissue of infans I children. δ^15^N values below average adult levels have been reported previously for bone and dentine collagen of postweaning children (e.g., [16, 48-49]). This is considered to reflect protein from low trophic level source e.g., vegetable foodstuffs in the diet of children and adolescents in comparison to adults. One likely scenario is the introduction of increasing amounts of cereal gruel as a substitute for breastmilk. It is striking that an age-dependent change in δ^15^N is observed across populations and cultures. This suggests that physiological factors could play a role e.g., in connection with physical growth [50-52]. This is supported by evidence for age-related diet-tissue spacing of δ^15^N values [53]. Therefore, we do not assume that this specific pattern in postweaning subadult individuals can exclusively attributed to limited access to animal protein, but that physiological processes probably also play a role. Overall, we decided to exclude infans I samples from further comparisons.

A comparison of excavation sites near Munich shows that individuals from Unterhaching that likely represent a higher social class show increased nitrogen ratios and also slightly decreased carbon ratios (Table S3.1.1.4.2) than most people from Perlach. This might indicate that they had access to different food sources e.g., better access to higher protein resources, but sample sizes are small especially that of Unterhaching (n=6). Despite social status, non-local origin of most individuals from Unterhaching as revealed through strontium isotope analysis might be reasonable for different dietary patterns. Comparison of excavation sites near Regensburg reveals that groups from Burgweinting (BW) show no distinct difference in stable light isotopes among each other or to Irlmauth (IRM) (Mann-Whitney U Test: p(δ^13^C)=0.4526, p(δ^15^N)=0.2411). Also, Hakenbeck et al. [5] revealed no evidence for a relationship between burial wealth and diet for individuals from Altenerding and Straubing.

Isotopic human bone collagen data from different study sites show large overlap like expected for nearby sites with rather similar ecogeographic conditions. But we observe some local trends. Human bone δ^15^N values in Munich-Perlach are significantly decreased compared to most other sites. Individuals from Munich-Perlach also show significantly increased δ^13^C values than populations at all other sides (Table S3.1.1.4.2) An environmental factor that might lead to those differences is the position of Munich-Perlach on the *Munich Gravel Plain*. Due to the rapid percolation of water through the gravel the area around Munich might be less humid than other regions, which could explain slightly increased carbon ratios overall [43, 54]. But a higher aridity like it might be suggested for the *Gravel Plain* as a steppe environment is also assumed to increased nitrogen ratios of primary producers [55] and consumers, which contradicts the observed trend in human data. Since we did not find any regional differences in the animal bones, site-specific differences in human data would be more likely due to different subsistence strategies rather than regional ecological variation. Elevated δ^13^C and decreased δ^15^N ratios of human samples from Munich-Perlach can be explained by lower amounts of animal-derived foodstuffs but higher contents of plants or plant parts with increased carbon ratios (including millet in basic diet) and or lower amounts of freshwater fish in diet. Another explanation that does not involve less animal protein would be that land use could have been less intensive without or even with less manuring. However, animal samples from the area of Munich (ASB) are underrepresented (n=4) in faunal data, therefore ecological variation cannot be rule out completely. The analysis of more faunal samples that better cover study regions but also additional archaeobotanical remains might contribute to verify that. There is also a difference found for δ^13^C mean values between the populations of Altenerding (-19.6‰) and Irlmauth (-20.1‰). Individuals from Altenerding show increased carbon ratios (Table S3.1.1.4.2). This might also indicate some site-specific ecogeographic or dietary features, but the difference is less pronounced than the divergence between Munich-Perlach and other sites.

### S3.1.1 Statistics

#### S3.1.1.1 Comparison of genders

To investigate dietary differences between sexes stable light isotopic data in bone collagen of individuals determined as rather male or male are grouped and compared to ratios of rather female and female individuals. Individuals with indetermined sex including all infans I children were excluded.

Men and women show equal stable light isotopic ratios (Table S3.1.1.1.1). But we observe a bigger range and higher variance of both isotopic ratios, but especially for carbon (Table S3.1.1.1.2).

Table S3.1.1.1.1: Comparison of stable light isotopic ratios in bone collagen of men and women.

| Value | Sex | N | Mean | SD | Sig. Tests for Normality | | Sig. 2-sided Test^b^ | |
| --- | --- | --- | --- | --- | --- | --- | --- | --- |
|  |  |  |  |  | Kolmogorov-Smirnov^a^ | Shapiro-Wilk | Mann-Whitney U | T-Test |
| δ^13^C [‰] | men | 60 | -19.73 | 0.49 | 0.200* | 0.184 | 0.513 | (0.193^c^) |
|  | women | 93 | -19.60 | 0.81 | 0.021 | <0.001 |  |  |
| δ^15^N [‰] | men | 59 | 9.25 | 0.66 | 0.200* | 0.897 | (0.540) | 0.445^d^ |
|  | women | 93 | 9.17 | 0.68 | 0.200* | 0.516 |  |  |
| a Lilliefors Significance Correction  * This is a lower bound of the true significance.  b Asymptotic significances are displayed. The significance level is 0.05.  c Equal variances not assumed (Levene Test).  d Equal variances assumed (Levene Test) | | | | | | | | |

Table S3.2.1.1.2: Descriptives of stable light isotopic ratios in bone collagen of men and women.

| Value | Sex | N | Mean | SD | Min | Max | Range | Variance |
| --- | --- | --- | --- | --- | --- | --- | --- | --- |
| δ^13^C [‰] | men | 60 | -19.73 | 0.49 | -20.95 | -18.34 | 2.61 | 0.236 |
|  | women | 93 | -19.60 | 0.81 | -20.93 | -15.03 | 5.90 | 0.652 |
| δ^15^N [‰] | men | 59 | 9.25 | 0.66 | 7.90 | 10.70 | 2.80 | 0.434 |
|  | women | 93 | 9.17 | 0.68 | 7.55 | 11.20 | 3.65 | 0.460 |

We also observe no difference of stable light isotopic ratios of men and women from different regions (Table S3.1.1.1.3) or excavation sites (Table S3.1.1.1.4). δ^15^N mean values of men are slightly higher in AED, STB, IRM and PEL. In suspected elite groups BWA, BWB and UTH women show more increased δ^15^N mean values than men (BWA, BWB) or at least an equal δ^15^N mean (UTH). Inside study regions or at excavation sites sample sizes are small and unbalanced which is why these results remain unconfirmed.

Table S3.1.1.1.3: Comparison of stable light isotopic ratios in bone collagen of men and women from different regional groups. Only non-parametric test performed due to small sample sizes.

| Site | Value | Sex | N | Mean | SD | Sig. Tests for Normality | | Sig. 2-sided Test^b^ |
| --- | --- | --- | --- | --- | --- | --- | --- | --- |
|  |  |  |  |  |  | Kolmogorov-Smirnov^a^ | Shapiro-Wilk | Mann-Whitney U |
| AED | δ^13^C [‰] | men | 19 | -19.67 | 0.36 | 0.200^*^ | 0.995 | 0.484 |
|  |  | women | 25 | -19.46 | 0.78 | 0.200^*^ | 0.002 |  |
|  | δ^15^N [‰] | men | 19 | 9.51 | 0.59 | 0.200^*^ | 0.999 | 0.387 |
|  |  | women | 25 | 9.41 | 0.69 | 0.121 | 0.162 |  |
| STB | δ^13^C [‰] | men | 12 | -19.92 | 0.42 | 0.200^*^ | 0.971 | 0.365^c^ |
|  |  | women | 21 | -19.75 | 0.43 | 0.200^*^ | 0.592 |  |
|  | δ^15^N [‰] | men | 12 | 9.59 | 0.50 | 0.200^*^ | 0.413 | 0.228^c^ |
|  |  | women | 21 | 9.36 | 0.46 | 0.177 | 0.112 |  |
| BW+IRM+AEH | δ^13^C [‰] | men | 16 | -20.03 | 0.43 | 0.155 | 0.418 | 0.928 |
|  |  | women | 31 | -19.86 | 1.04 | 0.002 | <0.001 |  |
|  | δ^15^N [‰] | men | 16 | 9.10 | 0.73 | 0.200^*^ | 0.674 | 0.425 |
|  |  | women | 31 | 9.21 | 0.62 | 0.200^*^ | 0.380 |  |
| UTH+PEL | δ^13^C [‰] | men | 13 | -19.29 | 0.45 | 0.091 | 0.009 | 0.083^c^ |
|  |  | women | 16 | -19.10 | 0.41 | 0.200^*^ | 0.601 |  |
|  | δ^15^N [‰] | men | 13 | 8.75 | 0.45 | 0.200^*^ | 0.177 | 0.144^c^ |
|  |  | women | 16 | 8.44 | 0.56 | 0.200^*^ | 0.625 |  |
| a Lilliefors Significance Correction  * This is a lower bound of the true significance.  b Asymptotic significances are displayed. The significance level is 0.05.  c Exact significance is displayed. | | | | | | | | |

Table S3.1.1.1.4: Comparison of stable light isotopic ratios in bone collagen of men and women at excavation sites around Regensburg and Munich. Only non-parametric test performed due to small sample sizes.

| Site | Value | Sex | N | Mean | SD | Sig. 2-sided Test^a^ |
| --- | --- | --- | --- | --- | --- | --- |
|  |  |  |  |  |  | Mann-Whitney U^b^ |
| IRM | δ^13^C [‰] | men | 8 | -19.95 | 0.39 | 0.423 |
|  |  | women | 9 | -20.16 | 0.32 |  |
|  | δ^15^N [‰] | men | 8 | 9.19 | 0.89 | 0.815 |
|  |  | women | 9 | 8.93 | 0.49 |  |
| BW | δ^13^C [‰] | men | 8 | -20.11 | 0.48 | 0.756 |
|  |  | women | 21 | -19.75 | 1.23 |  |
|  | δ^15^N [‰] | men | 8 | 9.00 | 0.57 | 0.257 |
|  |  | women | 21 | 9.29 | 0.64 |  |
| PEL | δ^15^N [‰] | men | 10 | -19.21 | 0.49 | 0.186 |
|  |  | women | 13 | -19.06 | 0.44 |  |
|  | δ^15^N [‰] | men | 10 | 8.68 | 0.46 | 0.131 |
|  |  | women | 13 | 8.32 | 0.53 |  |
| UTH | δ^13^C [‰] | men | 3 | -19.53 | 0.12 | 0.200 |
|  |  | women | 3 | -19.29 | 0.17 |  |
|  | δ^15^N [‰] | men | 3 | 8.96 | 0.41 | >0.999 |
|  |  | women | 3 | 9.00 | 0.28 |  |
| a Asymptotic significances are displayed. The significance level is 0.05.  b Exact significance is displayed. | | | | | |  |

#### S3.1.1.2 Comparison of age groups

To investigate dietary differences between individuals of different age stable light isotopic ratios in bone collagen of individuals grouped into the following age categories are compared: infans I (0-6 y), infans II (7-12 y), juvenile (13-20 y), adult (20-40 y), adult-mature (30-50 y), mature (40-60 y), mature-senile (50-60+ y) and senile (60+ y).

We observe no significant difference in δ^13^C ratios between age groups (Fig S3.1.1.2.1, Table S3.1.1.2.1), but δ^15^N of age groups differs significantly (Fig S3.1.1.2.2, Table S3.1.1.2.1).

Table S3.1.1.2.1: Comparison of stable light isotopes in bone collagen of age groups.

| Value | Age | N | Mean | SD | Sig. Tests for Normality | | Sig. 2-sided Test^b^ | |
| --- | --- | --- | --- | --- | --- | --- | --- | --- |
|  |  |  |  |  | Kolmogorov-Smirnov^a^ | Shapiro-Wilk | Kruskal-Wallis^c^ | ANOVA |
| δ^13^C [‰] | infans I | 11 | -19.59 | 0.68 | 0.036 | 0.064 | 0.605 | (0.817) |
|  | infans II | 4 | -19.47 | 0.41 | - | 0.397 |  |  |
|  | juvenile | 7 | -19.66 | 0.59 | 0.200^*^ | 0.212 |  |  |
|  | adult | 34 | -19.63 | 1.03 | 0.012 | <0.001 |  |  |
|  | adult-mature | 20 | -19.57 | 0.85 | 0.149 | 0.007 |  |  |
|  | mature | 57 | -19.78 | 0.47 | 0.200^*^ | 0.720 |  |  |
|  | mature-senile | 3 | -19.40 | 0.46 | - | 0.637 |  |  |
|  | senile | 30 | -19.54 | 0.56 | 0.103 | 0.017 |  |  |
| δ^15^N [‰] | infans I | 11 | 9.21 | 1.11 | 0.200^*^ | 0.364 | (0.005) | 0.005 |
|  | infans II | 4 | 8.56 | 0.84 | - | 0.587 |  |  |
|  | juvenile | 7 | 8.68 | 0.37 | 0.200^*^ | 0.924 |  |  |
|  | adult | 34 | 8.99 | 0.74 | 0.200^*^ | 0.636 |  |  |
|  | adult-mature | 20 | 9.10 | 0.62 | 0.200* | 0.662 |  |  |
|  | mature | 57 | 9.27 | 0.54 | 0.200^*^ | 0.965 |  |  |
|  | mature-senile | 3 | 9.57 | 0.55 | - | 0.909 |  |  |
|  | senile | 30 | 9.55 | 0.68 | 0.200^*^ | 0.898 |  |  |
| a Lilliefors Significance Correction  * This is a lower bound of the true significance.  b Asymptotic significances are displayed. The significance level is 0.05.  c The test statistic is adjusted for ties. | | | | | | | | |


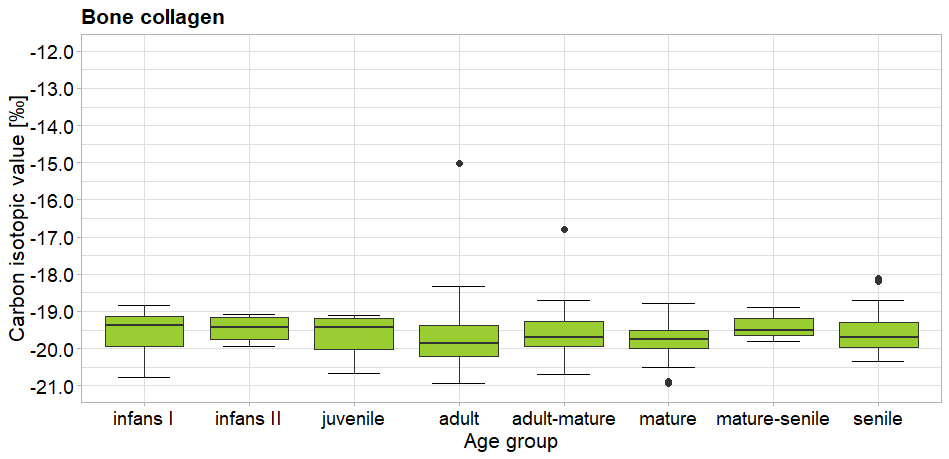


**Fig S3.1.1.2.1: Boxplot of δ^13^C in bone collagen of age groups.**

Boxes represent upper and lower quartiles. Horizontal lines inside boxes show median values. Whiskers are defined as 1.5*interquartile range. Single dots represent ratios defined as outliers (Illustration/calculation: R package ggplot2 [56-57]).


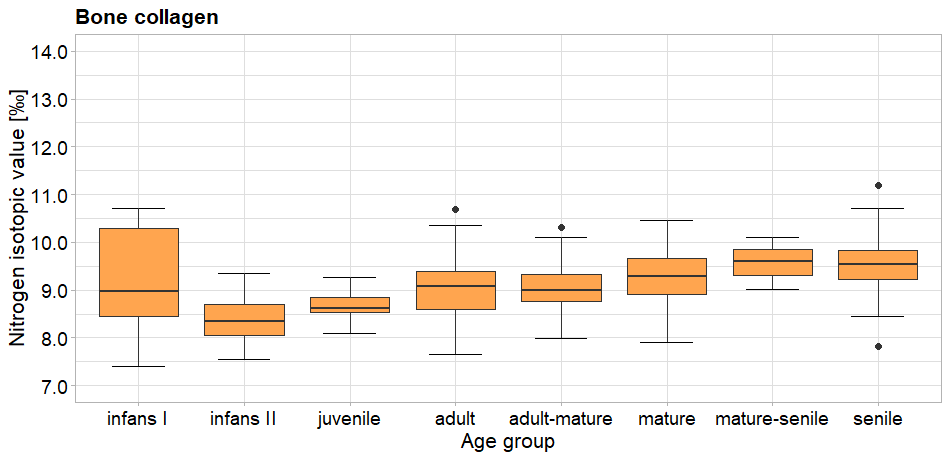


**Fig S3.1.1.2.2: Boxplot of δ^15^N of age groups.**

Boxes represent upper and lower quartiles. Horizontal lines inside boxes show median values. Whiskers are defined as 1.5*interquartile range. Single dots represent ratios defined as outliers. (Illustration/calculation: R package ggplot2 [56-57])

For carbon ratios pairwise comparisons are not performed because the overall test does not show significant differences across samples. Pairwise comparisons of nitrogen ratios of age groups (Table S3.1.1.2.2) only revealed a significant difference between juveniles and senile individuals.

Table S3.1.1.2.2: Pairwise comparisons of δ^15^N in bone collagen of age groups.

| Value | Group 1 – Group 2 | Mean Diff. (1-2) | Sig. Parametric Post Hoc | Dunn’s Post Hoc^a^ |
| --- | --- | --- | --- | --- |
|  |  |  | Dunnett T3 | Adj. Sig.^b^ |
| δ^15^N [‰] | infans I – infans II | 0.64727 | 0.986 | (>0.999) |
|  | infans I – juvenile | 0.53156 | 0.956 | (>0.999) |
|  | infans I – adult | 0.22139 | >0.999 | (>0.999) |
|  | infans I – adult-mature | 0.11277 | >0.999 | (>0.999) |
|  | infans I – mature | -0.06203 | >0.999 | (>0.999) |
|  | infans I – mature-senile | -0.36273 | >0.999 | (>0.999) |
|  | infans I – senile | -0.33939 | >0.999 | (>0.999) |
|  | infans II – juvenile | -0.11571 | >0.999 | (>0.999) |
|  | infans II – adult | -0.42588 | 0.994 | (>0.999) |
|  | infans II – adult-mature | -0.53450 | 0.970 | (>0.999) |
|  | infans II – mature | -0.70930 | 0.833 | (>0.999) |
|  | infans II – mature-senile | -1.01000 | 0.735 | (>0.999) |
|  | infans II – senile | -0.98667 | 0.594 | (0.613) |
|  | juvenile – adult | -0.31017 | 0.909 | (>0.999) |
|  | juvenile – adult-mature | -0.41879 | 0.618 | (>0.999) |
|  | juvenile – mature | -0.59358 | 0.070 | (0.348) |
|  | juvenile – mature-senile | -0.89429 | 0.496 | (0.757) |
|  | juvenile – senile | -0.87095^*^ | 0.005 | (0.023) |
|  | adult – adult-mature | -0.10862 | >0.999 | (>0.999) |
|  | adult – mature | -0.28342 | 0.766 | (>0.999) |
|  | adult – mature-senile | -0.58412 | 0.811 | (>0.999) |
|  | adult – senile | -0.56078 | 0.065 | (0.048) |
|  | adult-mature – mature | -0.10862 | 0.999 | (>0.999) |
|  | adult-mature – mature-senile | -0.28342 | 0.924 | (>0.999) |
|  | adult-mature – senile | -0.58412 | 0.384 | (0.608) |
|  | mature – mature-senile | -0.30070 | 0.991 | (>0.999) |
|  | mature – senile | -0.27737 | 0.766 | (>0.999) |
|  | mature-senile – senile | 0.02333 | >0.999 | (>0.999) |
| a Each row tests the null hypothesis that the Group 1 and Group 2 distributions are the same. Asymptotic significances (2-sided tests) are displayed. The significance level is 0.05.  b Significance values have been adjusted by the Bonferroni correction for multiple tests. | | | | |

Additional even greater mean differences remain not significant due to extremely small sample sizes. To improve statistical significance age categories of infans I and infans II children as well as juveniles are grouped as subadults and adult-mature as well as mature-senile individuals are counted for the older age category (morphological age at death is mostly underestimated). Comparison of the resulting four age groups also reveal a significant difference in nitrogen ratios (Table S3.1.1.2.3).

Table S3.1.1.2.3: Comparison of stable light isotopes in bone collagen of age groups.

| Value | Group | N | Mean | SD | Sig. Tests for Normality | | Sig. 2-sided Test^b^ | |
| --- | --- | --- | --- | --- | --- | --- | --- | --- |
|  |  |  |  |  | Kolmogorov-Smirnov^a^ | Shapiro-Wilk | Kruskal-Wallis^c^ | ANOVA^d^ |
| δ^13^C [‰] | subadult | 22 | -19.59 | 0.59 | 0.064 | 0.020 | 0.247 | (0.554) |
|  | adult | 34 | -19.63 | 1.03 | 0.012 | <0.001 |  |  |
|  | adult-mature+mature | 77 | -19.73 | 0.59 | 0.164 | <0.001 |  |  |
|  | mature-senile+senile | 33 | -19.53 | 0.54 | 0.104 | 0.022 |  |  |
| δ^15^N [‰] | subadult | 22 | 8.92 | 0.90 | 0.200* | 0.349 | (0.001) | 0.001 |
|  | adult | 34 | 8.99 | 0.74 | 0.200* | 0.636 |  |  |
|  | adult-mature+mature | 77 | 9.22 | 0.56 | 0.200* | 0.786 |  |  |
|  | mature-senile+senile | 33 | 9.55 | 0.66 | 0.200* | 0.900 |  |  |
| a Lilliefors Significance Correction  * This is a lower bound of the true significance.  b Asymptotic significances are displayed. The significance level is 0.05.  c The test statistic is adjusted for ties.  d Equal variances assumed (Levene Test). | | | | | | | | |

Pairwise comparison δ^15^N values in bone collagen of age groups (Table S3.1.1.2.4) shows that mature-senile to senile individuals have significantly increased nitrogen ratios than subadults and adult to adult-mature individuals.

Table S3.1.1.2.4: Pairwise comparison of δ^15^N in bone collagen of age groups.

| Value | Group 1 – Group 2 | Mean Diff. (1-2) | Sig. Parametric Post Hoc | Wilcoxon^a^ |
| --- | --- | --- | --- | --- |
|  |  |  | Hochberg | Adj. Sig.^b^ |
| δ^15^N [‰] | subadult – adult | -0.06543 | >0.999 | (1.000) |
|  | subadult – adult-mature+mature | -0.30344 | 0.324 | (0.364) |
|  | subadult – mature-senile+senile | -0.62833 | 0.005 | (0.004) |
|  | adult – adult-mature+mature | -0.23801 | 0.419 | (0.754) |
|  | adult – mature-senile+senile | -0.56291 | 0.005 | (0.007) |
|  | adult-mature+mature – mature-senile+senile | -0.32489 | 0.121 | (0.123) |
| a Each row tests the null hypothesis that the Group 1 and Group 2 distributions are the same. Asymptotic significances (2-sided tests) are displayed. The significance level is 0.05.  b Significance values have been adjusted by the Bonferroni correction for multiple tests. | | | | |

δ^15^N mean values are generally slightly elevated from adult to senile age (Fig S3.1.1.2.2).

Also, dentine samples can be used to analyze dietary differences between ages. Stable light isotopic ratios of molar dentine sections were grouped into life phases of 1-3 years, 3-6 years, 6-9 years, 9-12 years, 12-15 years,15-18 years, and 18-21 years.

We also observe a change in δ^15^N with age (Table S3.1.1.2.5). Dentine sections forming between approximately 9-12 years show the most decreased ratios (Fig S3.1.1.2.3).

Table S3.1.1.2.5 Comparison of stable light isotopic ratios in dentine of age spans (y=years).

| Value | Age span | N | Mean | SD | Sig. Tests for Normality | | Sig. 2-sided Statistical Test^b^ |
| --- | --- | --- | --- | --- | --- | --- | --- |
|  |  |  |  |  | Kolmogorov-Smirnov^a^ | Shapiro-Wilk | Kruskal-Wallis^c^ |
| δ^13^C [‰] | 1-3 y | 14 | -16.76 | 1.71 | 0.147 | 0.357 | 0.654 |
|  | 3-6 y | 29 | -17.76 | 1.91 | 0.200^*^ | 0.104 |  |
|  | 6-9 y | 39 | -17.86 | 1.98 | 0.111 | 0.013 |  |
|  | 9-12 y | 17 | -17.98 | 2.36 | 0.062 | 0.020 |  |
|  | 12-15 y | 31 | -17.48 | 2.38 | <0.001 | 0.002 |  |
|  | 15-18 y | 17 | -16.99 | 2.89 | 0.112 | 0.041 |  |
|  | 18-21 y | 13 | -17.34 | 2.75 | 0.031 | 0.042 |  |
| δ^15^N [‰] | 1-3 y | 14 | 10.97 | 1.49 | 0.200^*^ | 0.947 | 0.039 |
|  | 3-6 y | 29 | 9.77 | 1.09 | <0.001 | 0.007 |  |
|  | 6-9 y | 39 | 9.84 | 1.33 | <0.001 | 0.002 |  |
|  | 9-12 y | 17 | 9.44 | 1.59 | <0.001 | <0.001 |  |
|  | 12-15 y | 31 | 9.76 | 1.43 | 0.007 | 0.003 |  |
|  | 15-18 y | 17 | 9.86 | 1.55 | 0.200^*^ | 0.073 |  |
|  | 18-21 y | 13 | 10.34 | 1.56 | 0.200^*^ | 0.137 |  |
| a Lilliefors Significance Correction  * This is a lower bound of the true significance.  b Asymptotic significances are displayed. The significance level is 0.05.  c The test statistic is adjusted for ties. | | | | | | | |


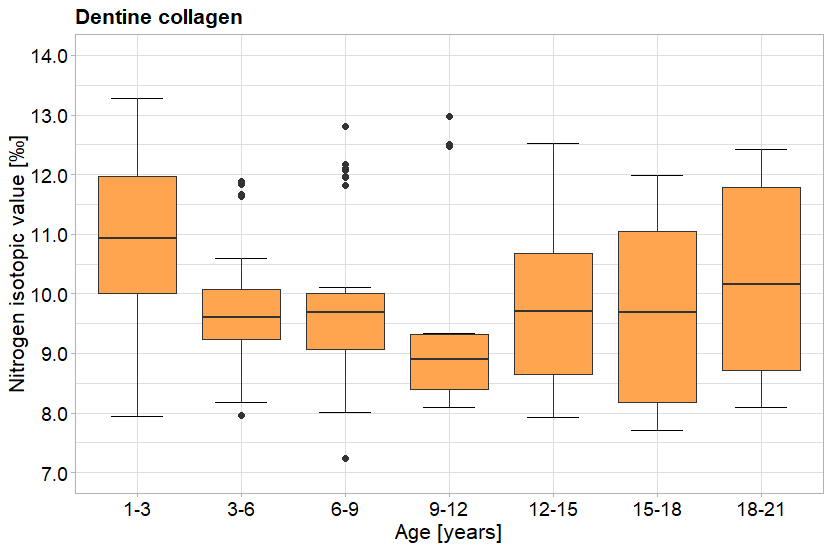


**Fig S3.1.1.2.3: Boxplot of δ^15^N in dentine collagen of age spans.**

Boxes represent upper and lower quartiles. Horizontal lines inside boxes show median values. Whiskers are defined as 1.5*interquartile range. Single dots represent ratios defined as outliers. (Illustration/calculation: R package ggplot2 [56-57])


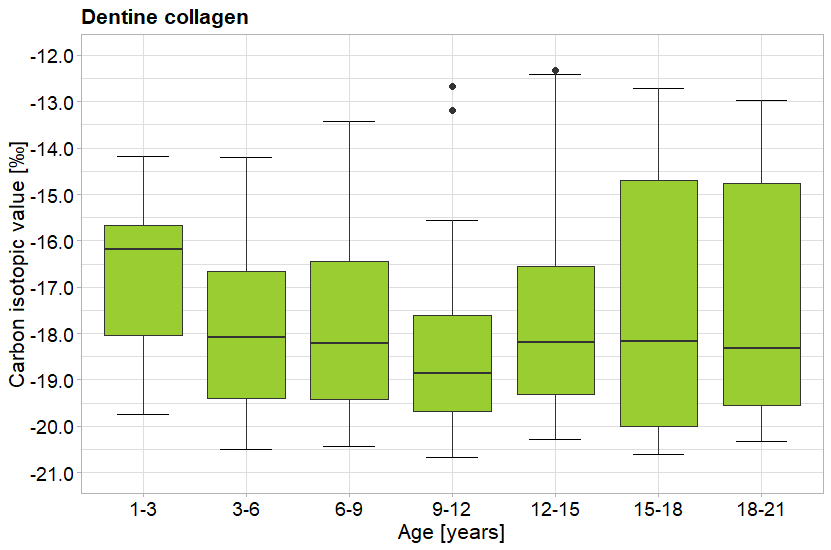


**Fig S3.1.1.2.4: Boxplot of δ^13^C in dentine collagen of age spans.**

Boxes represent upper and lower quartiles. Horizontal lines inside boxes show median values. Whiskers are defined as 1.5*interquartile range. Single dots represent ratios defined as outliers. (Illustration/calculation: R package ggplot2 [56-57])

Table S3.1.1.2.6: Pairwise comparison of δ^15^N in dentine of different age spans (y=years).

| Value | Span 1 – Span 2 | Dunn’s Post Hoc^b^ |
| --- | --- | --- |
|  |  | Adj. Sig.^c^ |
| δ^15^N [‰] | 1-3 y – 3-6 y | 0.269 |
|  | 1-3 y – 6-9 y | 0.392 |
|  | 1-3 y – 9-12 y | 0.020 |
|  | 1-3 y – 12-15 y | 0.220 |
|  | 1-3 y – 15-18 y | 0.575 |
|  | 1-3 y – 18-21 y | >0.999 |
|  | 3-6 y – 6-9 y | >0.999 |
|  | 3-6 y – 9-12 y | >0.999 |
|  | 3-6 y – 12-15 y | >0.999 |
|  | 3-6 y – 15-18 y | >0.999 |
|  | 3-6 y – 18-21 y | >0.999 |
|  | 6-9 y – 9-12 y | >0.999 |
|  | 6-9 y – 12-15 y | >0.999 |
|  | 6-9 y – 15-18 y | >0.999 |
|  | 6-9 y – 18-21 y | >0.999 |
|  | 9-12 y – 12-15 y | >0.999 |
|  | 9-12 y – 15-18 y | >0.999 |
|  | 9-12 y – 18-21 y | 0.560 |
|  | 12-15 y – 15-18 y | >0.999 |
|  | 12-15 – 18-21 y | >0.999 |
|  | 15-18 y – 18-21 y | >0.999 |
| b Each row tests the null hypothesis that the Span 1 and Span 2 distributions are the same. Asymptotic significances (2-sided tests) are displayed. The significance level is 0.05.  c Significance values have been adjusted by the Bonferroni correction for multiple tests. | | |

#### S3.1.1.3 Comparison of “locals” and non-locals revealed by stable strontium isotope analysis

To investigate dietary differences between migrants and “locals”, stable light isotopic ratios of bone collagen of “local” and non-local individuals as revealed by stable strontium isotope analysis are compared. Migrants are identified through 99% HDIs. Data of infans I children are excluded.

No significant difference between migrants and “local” individuals is observed (Table S3.1.1.3.1).

Table S3.1.1.3.1: Comparison of stable light isotopic ratios in bone collagen of “local” and non-local individuals as revealed by stable strontium isotope analysis (99% HDIs) at study regions.

| Region | Value | Provenance | N | Mean | SD | Sig. Tests for Normality | | Sig. 2-sided Statistical Test^b^ | |
| --- | --- | --- | --- | --- | --- | --- | --- | --- | --- |
|  |  |  |  |  |  | Kolmogrov-Smirnov | Shapiro-Wilk | Mann-Whithney U^c^ | T-Test |
| AED | δ^13^C [‰] | "local" | 29 | -19.50 | 0.72 | 0.045 | <0.001 | 0.805 | 0.531^e^ |
|  |  | non-local | 14 | -19.64 | 0.45 | 0.200* | 0.943 |  |  |
|  | δ^15^N [‰] | "local" | 29 | 9.50 | 0.67 | 0.014 | 0.023 | 1.000 | 0.543^e^ |
|  |  | non-local | 14 | 9.36 | 0.61 | 0.151 | 0.179 |  |  |
| STB | δ^13^C [‰] | "local" | 29 | -19.84 | 0.40 | 0.200* | 0.675 | 0.568 | 0.721^e^ |
|  |  | non-local | 2 | -19.94 | 0.06 | - | - |  |  |
|  | δ^15^N [‰] | "local" | 29 | 9.45 | 0.47 | 0.164 | 0.102 | 1.000 | 0.837^e^ |
|  |  | non-local | 2 | 9.52 | 1.02 | - | - |  |  |
| REG | δ^13^C [‰] | "local" | 37 | -19.83 | 0.95 | <0.001 | <0.001 | 0.152 | 0.205^e^ |
|  |  | non-local | 9 | -20.25 | 0.47 | 0.200* | 0.186 |  |  |
|  | δ^15^N [‰] | "local" | 37 | 9.21 | 0.70 | 0.200* | 0.722 | 0.786 | 0.824^f^ |
|  |  | non-local | 9 | 9.16 | 0.29 | 0.200* | 0.907 |  |  |
| MUC | δ^13^C [‰] | "local" | 21 | -19.13 | 0.47 | 0.200* | 0.390 | 0.349 | 0.284^e^ |
|  |  | non-local | 8 | -19.33 | 0.26 | 0.060 | 0.102 |  |  |
|  | δ^15^N [‰] | "local" | 21 | 8.48 | 0.53 | 0.200* | 0.164 | 0.083 | 0.088^e^ |
|  |  | non-local | 8 | 8.85 | 0.44 | 0.148 | 0.129 |  |  |
| a Lilliefors Significance Correction  * This is a lower bound of the true significance.  b Asymptotic significances are displayed. The significance level is 0.05.  c The test statistic is adjusted for ties. Exact significance is displayed for this test.  e Equal variances assumed (Levene Test)  f Equal variances not assumed (Levene Test) | | | | | | | | | |

#### S3.1.1.4 Comparison of excavation sites

To investigate site-specific differences stable light isotopes of human bone collagen of excavation sites except Alteglofsheim (AEH) are compared. Data of infans I children is excluded.

Significant differences in δ^13^C and δ^15^N between excavation sites is found (Table S3.1.1.4.1). Pairwise comparisons revealed differences between single sites (Table S3.1.1.4.2).

Table S3.1.1.4.1: Comparison of stable light isotopic ratios in bone collagen of excavation sites (AED=Altenerding, STB=Straubing-Bajuwarenstraße, IRM=Irlmauth, BW=Burgweinting, PEL=Munich-Perlach, UTH=Unterhaching).

| Value | Site | N | Mean | SD | Sig. Tests for Normality | | Sig. 2-sided Statistical Test^b^ |
| --- | --- | --- | --- | --- | --- | --- | --- |
|  |  |  |  |  | Kolmogorov-Smirnov^a^ | Shapiro-Wilk | Kruskal-Wallis^c^ |
| δ^13^C [‰] | AED | 44 | -19.55 | 0.63 | 0.058 | <0.001 | <0.001 |
|  | STB | 33 | -19.81 | 0.43 | 0.200* | 0.432 |  |
|  | IRM | 17 | -20.06 | 0.36 | 0.200* | 0.882 |  |
|  | BW | 31 | -19.85 | 1.05 | 0.049 | 0.042 |  |
|  | PEL | 23 | -19.13 | 0.45 | 0.200* | 0.283 |  |
|  | UTH | 6 | -19.41 | 0.19 | 0.200* | 0.545 |  |
| δ^15^N [‰] | AED | 44 | 9.45 | 0.64 | <0.001 | <0.001 | <0.001 |
|  | STB | 33 | 9.44 | 0.48 | 0.200* | 0.507 |  |
|  | IRM | 17 | 9.06 | 0.70 | 0.200* | 0.418 |  |
|  | BW | 31 | 9.21 | 0.60 | 0.200* | 0.161 |  |
|  | PEL | 23 | 8.48 | 0.52 | 0.200* | 0.583 |  |
|  | UTH | 6 | 8.98 | 0.31 | 0.200* | 0.672 |  |
| a Lilliefors Significance Correction  * This is a lower bound of the true significance.  b Asymptotic significances are displayed. The significance level is 0.05.  c The test statistic is adjusted for ties. | | | | | | | |

Table S3.1.1.4.2: Pairwise comparison of stable light isotopic ratios in bone collagen of excavation sites (AED=Altenerding, STB=Straubing-Bajuwarenstraße, IRM=Irlmauth, BW=Burgweinting, PEL=Munich-Perlach, UTH=Unterhaching).

| Value | Group 1 – Group 2 | Test Statistic | Std. Error | Std. Test Statistic | Sig. | Adj. Sig.^a^ |
| --- | --- | --- | --- | --- | --- | --- |
| δ^13^C [‰] | AED – STB | 17.561 | 10.269 | 1.710 | 0.087 | 1.000 |
|  | AED – IRM | 39.711 | 12.734 | 3.118 | 0.002 | 0.027 |
|  | AED – BW | 29.424 | 10.456 | 2.814 | 0.005 | 0.073 |
|  | AED – PEL | -36.753 | 11.474 | -3.203 | 0.001 | 0.020 |
|  | AED – UTH | -23.068 | 19.406 | -1.189 | 0.235 | 1.000 |
|  | STB – IRM | 22.151 | 13.312 | 1.664 | 0.096 | 1.000 |
|  | STB – BW | 11.863 | 11.153 | 1.064 | 0.287 | 1.000 |
|  | STB – PEL | -54.314 | 12.112 | -4.484 | <0.001 | 0.000 |
|  | STB – UTH | -40.629 | 19.790 | -2.053 | 0.040 | 0.601 |
|  | IRM – BW | -10.287 | 13.458 | -0.764 | 0.445 | 1.000 |
|  | IRM – PEL | -76.464 | 14.263 | -5.361 | <0.001 | 0.000 |
|  | IRM – UTH | -62.779 | 21.175 | -2.965 | 0.003 | 0.045 |
|  | BW – PEL | -66.177 | 12.272 | -5.393 | <0.001 | 0.000 |
|  | BW – UTH | -52.492 | 19.888 | -2.639 | 0.008 | 0.125 |
|  | PEL – UTH | 13.685 | 20.442 | 0.669 | 0.503 | 1.000 |
| δ^15^N [‰] | AED – STB | -0.061 | 10.269 | -0.006 | 0.995 | 1.000 |
|  | AED – IRM | 28.791 | 12.735 | 2.261 | 0.024 | 0.357 |
|  | AED – BW | 14.587 | 10.457 | 1.395 | 0.163 | 1.000 |
|  | AED – PEL | 63.105 | 11.474 | 5.500 | <0.001 | 0.000 |
|  | AED – UTH | 35.492 | 19.407 | 1.829 | 0.067 | 1.000 |
|  | STB – IRM | 28.852 | 13.313 | 2.167 | 0.030 | 0.453 |
|  | STB – BW | 14.647 | 11.154 | 1.313 | 0.189 | 1.000 |
|  | STB – PEL | 63.165 | 12.113 | 5.215 | <0.001 | 0.000 |
|  | STB – UTH | 35.553 | 19.792 | 1.796 | 0.072 | 1.000 |
|  | IRM – BW | -14.205 | 13.459 | -1.055 | 0.291 | 1.000 |
|  | IRM – PEL | 34.313 | 14.263 | 2.406 | 0.016 | 0.242 |
|  | IRM – UTH | 6.701 | 21.176 | 0.316 | 0.752 | 1.000 |
|  | BW – PEL | 48.518 | 12.273 | 3.953 | <0.001 | 0.001 |
|  | BW – UTH | 20.906 | 19.890 | 1.051 | 0.293 | 1.000 |
|  | PEL – UTH | -27.612 | 20.443 | -1.351 | 0.177 | 1.000 |
| Each row tests the null hypothesis that the Group 1 and Group 2 distributions are the same.  Asymptotic significances (2-sided tests) are displayed. The significance level is 0.05.  a Significance values have been adjusted by the Bonferroni correction for multiple tests. | | | | | | |

## S3.2 Childhood diet

Although children might also change residency with their parents or other adults, it is assumed that individuals more often spent their childhood at place of birth. Therefore, migrants are likely to show deviating stable light isotopic ratios in tooth dentine due to the stay in another ecosystem at least more often than locals inhabitants. To investigate this in our samples, we analyzed bulk root dentine (S1.1 Table) of 24 individuals, of which 13 individuals show at least one evidence of a foreign origin, defined as notable attributes: (1) ^87^Sr/^86^Sr in enamel or (2) δ^13^C and δ^15^N in bone collagen which are different from the main population (S2.3.3.3 Table, S2.4.3 Table) or (3) the presence of ACD. Two additional individuals show potentially notable attributes: Isotopic ratios outside 90% HDIs. Deviating values in bone collagen of individuals of advanced age due to the consumption of non-local resources might also be observed in dentine which mirrors childhood diet if the migration event happened later in life. If childhood diet of these individuals is evaluated to be inside the population’s “common variability” it could mean that individuals had access to non-local food stuffs later and possibly changed residence more than one time. Nine “local” individuals without notable attributes were chosen randomly.

“Bulk” dentine (S1.1 Table) is analyzed, excluding crown sections to minimize the effect of breastfeeding and weaning.

First molar bulk root dentine represents diet between approximately 3.5 to 9.5 years of age. Rib bone of most individuals provides information about a period towards the end of a person's life [58], though contributions from earlier years are possible [59]. Long bone samples of AED_249 (clavicula) and BWA_10071 (femur) are said to reflect a long-term average of individual’s diet [59]. All individuals reached adult to senile age. Thus, dentine and bone collagen of all individuals are likely to refer to dietary patterns in separated life stages.

Mean differences between bone and dentine collagen of individuals without notable attributes are 0.7 for δ^13^C and 0.5 for δ^15^N. Mean differences of individuals with notable attributes are more than twice as big (1.5 for δ^13^C and 1.1 for δ^15^N). The difference in δ^15^N is significantly higher in individuals with notable attributes (Table S3.2.1).

Table S3.2.1: Comparison of differences between dentine and bone collagen of individuals with notable attributes (isotopic outliers or presence of ACD) and individuals without notable attributes.

| Value | Notable attribute | N | Mean | SD | Sig. Tests for Normality | | Sig. 2-sided Test^b^ | |
| --- | --- | --- | --- | --- | --- | --- | --- | --- |
|  |  |  |  |  | Kolmogorov-Smirnov^a^ | Shapiro-Wilk | Mann-Whitney U | T-Test^d^ |
| δ^13^C [‰] | No | 9 | 0.7 | 0.5 | 0.071 | 0.902 | 0.324^c^ | 0.074 |
|  | Yes | 13 | 1.5 | 1.4 | 0.024 | 0.864 |  |  |
| δ^15^N [‰] | No | 9 | 0.5 | 0.3 | 0.200* | 0.943 | 0.011^c^ | 0.003 |
|  | Yes | 13 | 1.1 | 0.7 | 0.200* | 0.943 |  |  |
| a Lilliefors Significance Correction.  * This is a lower bound of the true significance.  b Asymptotic significances are displayed. The significance level is 0.05.  c Exact significance is displayed.  d Equal variances not assumed (Levene Test). | | | | | | | | |

As for bone stable light isotopic ratios in dentine are considered to be deviant if one or both ratios are found outside 99% HDIs of the population, and potentially deviant if at least one value lay outside 90% HDIs. Not deviant values are assumed if both ratios lay inside 90% HDIs (Table S3.2.2).

Table S3.2.2: Notable attributes and childhood diet. Notable attributes are ^86^Sr/^87^Sr ratios in enamel, δ^13^C or δ^15^N ratios in bone collagen outside 99% HDIs of the population or the presence of ACD, potentially notable attributes are isotopic ratios outside 90% HDIs, not notable features are isotopic values inside 90% HDIs. Childhood diet as indicated by stable light isotopes in bulk root dentine is considered as deviant if δ^13^C or δ^15^N ratios are found outside 99% HDIs of the population, potentially deviant if values are found outside 90% HDIs and considered as not deviant if both values are found inside 90% HDIs.

| Individual | Sex | Age | Attribute (Isotopes in tooth enamel or bone collagen and skull shape) | Stable light isotopes in bulk root mean dentine collagen |
| --- | --- | --- | --- | --- |
| PEL_12 | f | adult | notable (^87^Sr/^86^Sr 99% HDI) | deviant (δ^13^C 90% HDI, δ^15^N 99% HDI) |
| AED_125* | f | mature | notable (ACD) | deviant (δ^13^C 99% HDI) |
| AED_211 | f | mature | notable (^87^Sr/^86^Sr 99% HDI) | deviant (δ^15^N 99% HDI) |
| AED_280 | m | adult-mature | potentially notable (δ^15^N bone value 90% HDI) | not deviant |
| AED_343 | f | mature | notable (^87^Sr/^86^Sr 99% HDI) | deviant (δ^13^C 99% HDI) |
| AED_492 | m | mature | notable (^87^Sr/^86^Sr 99% HDI) | not deviant |
| AED_501 | m | mature | notable (^87^Sr/^86^Sr 99% HDI) | not deviant |
| AED_513* | f | adult-mature | notable (ACD, South European ancestry) | deviant (δ^13^C/ δ^15^N 99% HDI) |
| AED_1129 | f | adult | potentially notable (Sr 90%HDI) | not deviant |
| AED_1143 | (m) | adult-mature | notable (^87^Sr/^86^Sr 99% HDI) | not deviant |
| STB_300 | f | mature | notable (^87^Sr/^86^Sr 99% HDI, South European ancestry) | potentially deviant (δ^13^C 90% HDI) |
| STB_361* | f | mature | notable (ACD) | deviant (δ^13^C 99% HDI) |
| STB_535* | f | mature | notable (ACD, South European ancestry) | deviant (δ^13^C 99% HDI) |
| BWA_10071 | f | adult | notable (δ^13^C/δ^15^N bone 99% HDI) | deviant (δ^13^C 99% HDI) |
| BWA_10254* | f | mature | notable (ACD, South European ancestry) | deviant (δ^13^C/ δ^15^N 99% HDI) |
| AED_92 | m | adult | not notable | not deviant |
| AED_105 | f | adult | not notable | not deviant |
| AED_154 | m | mature | not notable | potentially deviant (δ^13^C 90% HDI) |
| AED_204 | f | senile | not notable | not deviant |
| AED_249 | m | mature | not notable | not deviant |
| AED_825 | (f) | adult | not notable | not deviant |
| AED_1119 | f | adult-mature | not notable | not deviant |
| BWA_10075 | f | adult | not notable | not deviant |
| BWA_10255 | f | mature | not notable | not deviant |
| * ACD | | | | |

Chi-Square Test revealed a significant correlation between the presence of notable attributes and deviant stable light isotope values in childhood (Table S3.2.4).

Table S3.2.3: Crosstabulation (Attribute vs. Stable light isotopes in bulk root dentine collagen (DC)).

|  | | | Stable light isotopes in DC | | | Total |
| --- | --- | --- | --- | --- | --- | --- |
|  |  |  | not deviant | deviant | potentially deviant |  |
| Attribute | not notable | Count | 8 | 0 | 1 | 9 |
|  |  | Expected Count | 4.9 | 3.0 | 1.1 | 9.0 |
|  |  | % within Feature | 88.9% | 0.0% | 11.1% | 100.0% |
|  |  | 95% CI | 66.7-100.0% |  | 0.0-33.0% |  |
|  |  | % within DC | 61.5% | 0.0% | 50.0% | 37.5% |
|  |  | Adjusted residual | 2.6 | -2.9 | 0.4 |  |
|  |  | Pearson Chi Square | 0.0082 | 0.0033* | 0.7029 |  |
|  | notable | Count | 3 | 9 | 1 | 13 |
|  |  | Expected Count | 7.0 | 4.9 | 1.1 | 13.0 |
|  |  | % within Feature | 23.1% | 69.2% | 7.7% | 100.0% |
|  |  | 95% CI | 0.0-46.2% | 46.2-92.3% | 0.0-23.1% |  |
|  |  | % within DC | 23.1% | 100.0% | 50.0% | 54.2% |
|  |  | Adjusted residual | -3.3 | 3.5 | -0.1 |  |
|  |  | Pearson Chi Square | 0.0009* | 0.0005* | 0.9017 |  |
|  | potentially notable | Count | 2 | 0 | 0 | 2 |
|  |  | Expected Count | 1.1 | 0.8 | 0.2 | 2.0 |
|  |  | % within Feature | 100.0% | 0.0% | 0.0% | 100.0% |
|  |  | 95% CI |  |  |  |  |
|  |  | % within DC | 15.4% | 0.0% | 0.0% | 8.3% |
|  |  | Adjusted residual | 1.4 | -1.1 | -0.4 |  |
|  |  | Pearson Chi Square | 0.1742 | 0.2526 | 0.6561 |  |
| Total | | Count | 13 | 9 | 2 | 24 |
|  |  | Expected Count | 13.0 | 9.0 | 2.0 | 24.0 |
|  |  | % within Feature | 54.2% | 37.5% | 8.3% | 100.0% |
|  |  | % within DC | 100.0% | 100.0% | 100.0% | 100.0% |
| * significant after Bonferroni correction (p<0.0056) | | | | | | |

Table S3.2.4: Pearson Chi-Square Test.

|  | Value | df | Asymptotic Significance (2-sided) |
| --- | --- | --- | --- |
| Pearson Chi-Square | 12.970^a^ | 4 | 0.011 |
| Likelihood Ratio | 16.709 | 4 | 0.002 |
| Linear-by-Linear Association | 0.828 | 1 | 0.363 |
| N of Valid Cases | 24 |  |  |
| a 8 cells (88.9%) have expected count less than 5. The minimum expected count is 0.17. | | | |

69.2% (95% CI: 46.2–92.3%) of individuals with notable attributes show deviating stable light isotope ratios in first molar bulk root dentine. Except for one individual (AED_211) they all show increased carbon ratios favoring a higher contribution of millet to childhood diet. This group includes all five individuals with deformed skulls. Two females with ACD additionally show increased nitrogen ratios (AED_513*, AED_10254*) but also AED_211 and PEL_12 have elevated δ^15^N values that might refer to marine resources or foods from more arid and warm areas. Three individuals with deviant ^87^Sr/^86^Sr ratios, AED_492, AED_501 and AED_1143 (28.6%), and the two individuals with potentially notable attributes (AED_280, AED_1129) show stable light isotopic ratios that refer to ecosystems with comparable conditions to their burial places. This does not necessarily prove that individuals with potentially notable attributes are “local” individuals at all. But for AED_280 whose non-local origin due to a decreased nitrogen value was questionable favors an individual dietary pattern rather than the usage of non-local resources in a different habitat. One individual STB_300 shows a slightly increased δ^13^C value that corresponds to the cut of value of the local range (99% HDI) and indicates a potentially uncommon diet.

88.9% (95% CI: 66.7-100.0%) of individuals without notable attributes show stable light isotopic dentine ratios that also display “common” diet. But one individual AED_154 has a slightly increased carbon value that might indicate a more frequent consumption of C4 plants in related formation age and be linked to residency in another ecosystem. At first glance, this appears to be a small number, but it corresponds to more than 10 % of the individuals without notable features addressed as "local". If no additional information, but only stable strontium isotopes had been available, there would be even more.

“Local” individuals couldn’t be selected safely and must not belong to the actual local population, whereas migrants may show “common” dietary patterns in childhood if food spectrum at place of origin and burial site do not differ significantly. Therefore, we did not expect 100% agreement of the presence of notable features that indicate a non-local provenance and deviating childhood diet or “local” origin and “common” diet during childhood anyway, but we observe a clear cluster of deviating dietary patterns in potential migrants. An alternative explanation for different carbon or nitrogen dentine ratios independent of individual’s origin would be a more generally changed diet for children. But we do not observe a trend towards elevated carbon ratios or increased nitrogen ratios in bone collagen of young individuals (S3.1 Text). Overall, we propose that the connection between a non-local origin and a different diet in childhood is reliable and consider that analysis of dentine samples to be a useful tool in migration research.

## S3.3 Life-history

Serial analysis of dentine samples of first, second and third molars were undertaken exemplary on six individuals (Fig 8) to provide high resolution biographical data pointing out changes throughout an individual’s life. This not only shows patterns of diet in earlier life in addition to later life trends evidenced by bone stable light isotopes but fluctuations in micro-sampling profiles have been linked to dietary changes, migration, physiological stress, metabolic disorders, and/or nutritional deficiency [60].

The selection of individuals was limited by the availability of required molars. Unfortunately, this applied for only one female with deformed skull (BWA_10254*). Therefore, we included other non-local women identified through strontium isotope analysis (AED_343, PEL_12) and clearly outstanding dietary patterns in bone collagen (BWA_10071). In addition, two “local” females (AED_105, BWA_10075) were included for comparison.

Stable light isotopes of successive sections of the same molar but also sections of teeth (S1.3 Table) from overlapping time periods cannot always be perfectly aligned but show some bigger leaps. This is not unusual and due to the fact that the chosen method does not sample very precisely, and tooth structure is very complex. In general, it applies that dentine mineralization rates vary across teeth (crown vs. root) and between tooth types [61-62]. The varying number of depositional layers (of dentine) in each horizontal incremental section appears to result in varying isotopic ratios for the same period of life [60]. The imprecision is caused by rather diagonally running dentine layers increases within the root, which is why first sections of molars should be given greater attention in overlapping periods. However, tooth abrasion plays a role in the first section, which cannot always be accurately estimated and may therefore be greater than expected. Overall, the sampling method does not consider the actual direction of dentine growth layers and methods for age assignment of sequential dentine samples and alignment of results from different teeth are still flawed (for further discussion and approaches see [63-65]). Thus, the age period given in each data point is subject to an error. However, the general course can still be reconstructed reasonably well. To acknowledge the uncertainty, the included error bars represent the putative timespan each datapoint covers.

It is difficult to ascertain what degree of change within the same individual can be attributed to “normal” dietary variation. We observe changes in δ^15^N and δ^13^C between dentine of several molars but also remarkable changes between successive dentine sections of single molars. Dentine sections and even bulk dentine record diet over a shorter time period than bone collagen, making dentine samples more sensitive to short-term dietary changes. The difference between bulk root dentine of molars and bone samples can get even more pronounced.

Interestingly, although δ^15^N are more sensitive to short term change than δ^13^C [66] and previous studies observed higher intra-individual variability for δ^15^N values [67-68] we observe a remarkable variation in δ^13^C. Excluding data from the crown of first molars that show typical weaning pattern, the difference between the most increased and decreased ratio of dentine sections of each individual lies between 1.4‰ and 2.6‰ for δ^15^N and 1.0‰ and 5.4‰ for δ^13^C (Table S3.3.1). Variability in both isotopic ratios is slightly increased in comparison to other studies (e.g., [67-68]) because we included dentine sections of more than one tooth, taken from several previously identified migrants. More variable δ^13^C compared to δ^15^N indicate that some individuals experienced a change in diet regarding plant food components rather than animal protein.

| *Table S3.3.1: Standard deviation (SD) and range (R) of isotopic ratios in dentine sections of first molar (M1), second molar (M2) and third molar (M3).* | | | | | | | | | | | | | |
| --- | --- | --- | --- | --- | --- | --- | --- | --- | --- | --- | --- | --- | --- |
| Individual | value | M1 | | | | M2 | | M3 | | M1 – M3 | | M1_root_ – M3 | |
|  |  | SD | R | SD_root_ | R_root_ | SD | R | SD | R | SD | R | SD | R |
| AED_105 | δ^13^C | 0.34 | 1.08 | 0.25 | 0.61 | 0.17 | 0.43 | 0.22 | 0.62 | 0.35 | 1.5 | 0.31 | 1.03 |
|  | δ^15^N | 0.62 | 2.27 | 0.35 | 0.87 | 0.88 | 2.5 | 0.59 | 1.51 | 0.77 | 3.38 | 0.69 | 2.5 |
| AED_343 | δ^13^C | 0.91 | 2.72 | 0.65 | 1.53 | 0.68 | 1.88 | 0.23 | 0.77 | 1.34 | 4.84 | 1.06 | 3.64 |
|  | δ^15^N | 0.71 | 2.33 | 0.26 | 0.81 | 0.61 | 1.83 | 0.27 | 0.8 | 0.94 | 3.45 | 0.68 | 1.93 |
| BWA_10071 | δ^13^C | 0.42 | 1.57 | 0.37 | 1.07 | 0.83 | 1.17 | 1.17 | 3.08 | 0.83 | 3.08 | 0.87 | 3.08 |
|  | δ^15^N | 0.36 | 1.21 | 0.17 | 0.51 | 0.8 | 1.14 | 0.3 | 0.91 | 0.4 | 1.91 | 0.36 | 1.46 |
| BWA_10075 | δ^13^C | 0.7 | 2.22 | 0.09 | 0.25 | 0.47 | 1.69 | 0.28 | 0.7 | 1.18 | 3.07 | 1.19 | 3.07 |
|  | δ^15^N | 0.27 | 0.83 | 0.25 | 0.63 | 0.4 | 1.33 | 0.45 | 1.32 | 0.42 | 1.74 | 0.43 | 1.74 |
| BWA_10254* | δ^13^C | 0.68 | 1.95 | 0.63 | 1.71 | 0.61 | 1.61 | 0.77 | 2.15 | 1.62 | 5.42 | 1.63 | 5.42 |
|  | δ^15^N | 0.54 | 1.65 | 0.23 | 0.54 | 0.31 | 0.92 | 0.38 | 1.29 | 0.41 | 1.65 | 0.35 | 1.36 |
| PEL_12 | δ^13^C | 0.86 | 2.3 | 0.25 | 0.57 | 0.41 | 1.23 | 0.55 | 1.84 | 0.8 | 3.32 | 0.51 | 2.26 |
|  | δ^15^N | 1.2 | 3.02 | 0.17 | 0.37 | 0.38 | 1.14 | 0.84 | 2.11 | 0.97 | 3.56 | 0.75 | 2.6 |
| * ACD | | | | | | | | | | | | | |

Stable light isotope profiles of all individuals show fluctuations in isotopic ratios that can be attributed to each person’s unique experience. However, all profiles show a more or less pronounced drop down of both stable light isotopes in the first sections of the first molar due to weaning (e.g., [69]) (which is not the focus of this study but is part of a different publication Velte et al. in prep. [70]). They also show a more or less pronounced drop down of nitrogen ratios in younger age, which matches previous results and might indicate a lower protein diet but may also be connected to physiological features (e.g., [53]). We also found some opposing covariant courses of stable light isotopes, which might indicate phases of physiological stress (e.g., [71-73]).

For the adult female AED_105 averaged dentine ratios are consistent with bone (later life) ratios indicating a rather high level of stability in diet across the entire life course. Fluctuations of both stable light isotopes are likely to reflect “normal” variation. Especially carbon ratios show no remarkable changes. Nitrogen ratios are slightly decreased between approximately 8 and 12 years. This matches previous results and may indicates a less protein rich diet during that time or some physiological features. Afterwards δ^15^N is increased until the previous level possibly due to an increasing amount of animal protein.

Two women from Burgweinting (BWA_10071 and BWA_10254*) show remarkable increased δ^13^C values in subadult age that clearly indicate an uncommon diet and both profiles show significant changes in carbon (>2‰). The profile of BWA_10071 (aged 25-35 years) shows a drop of δ^13^C around the age of 15 years that seems to further align with the bone value. This indicates a change of location, presumably to Bavaria. Contrarily, BWA_10254* (aged 60+) shows an increase in δ^13^C around the same age that further departs from the decreased bone value. This also indicates a change of location but not to Bavaria. The extreme difference between both isotope ratios in early life (dentine) and later life (bone) indicate that her diet changed remarkably. The migration to Bavaria must have happened after the age of 20 years but not very close to death (in senile age), because her bone data likely reflects a blend of foreign and local diet (average of diet of several years before death).

Although not identified as non-local through previous analyses, BWA_10075 (aged 30-40) also shows a significant change in carbon ratios. Dentine sections of the second molar are approximately 2‰ elevated than those of the first and third molar. But in contrast to carbon ratios of BW_10071 and BW_10254*, values only slightly exceed the local range. Therefore, this pattern could also be interpreted as a sign for a temporal e.g., seasonal usage of unusual resources at the site. While in Late Antiquity and the Early Middle Ages a wide range of C3 crops were cultivated [45], millet was uncommon (e.g., [3, 74-75]) but known in Central Europe [76-78]. Because of its short growing period, high tolerance to poor soil and good storage conditions [36] millet is particularly suitable as “back-up-crop” in case of poor harvest or crop failure of more commonly cultivated C3 crops (e.g., [45]). Thus, the cultivation of millet in Southern Bavaria, at least temporarily, cannot be completely excluded. Albeit the drop in carbon ratios around the age of 15 years may also display a change in diet due to a change in residence and call her actual origin in question.

In contrast to BWA_10071, AED_343 shows a significant drop in carbon ratios (~2‰) in dentine sections of the second molar. Additional stronger fluctuations in nitrogen ratios with trend towards (~1.5‰) increased values rather indicate phase(s) of physiological stress, however, dietary changes cannot be ruled out. Carbon ratios in dentine of the third molar of AED_343 are found outside of the local range for Erding and differ from bone ratios. This indicates a change of diet and potentially residency later in life.

For PEL_12 nitrogen dentine ratios stabilize around the upper local limit after weaning which might indicate food stuffs from a region with a higher nitrogen baseline. Additionally, carbon ratios in dentine sections of the third molar are found at or above the local range for some time. The difference between dentine and bone ratios again indicates a change of dietary patterns linked to the migration to Munich in later life.

Changes in residence become visible in at least two profiles around the age of 15 years (BWA_10071, BWA_10254*). But most migrants likely came to Bavaria after the age of 20 years - outside the timeframe captured with dentine analysis of all three molars.

The serial analysis of stable light isotopes provides interesting hints about dietary changes, but a lot of aspects are not easy to explain and hard to interpret at present state of the art.

## S3.4 Patterns of mobility

We calculated proportion of non-locals and migrants as revealed by stable strontium isotope analysis to determine differences between sexes, settlement zones, and excavation sites. Infans I and II children were excluded from calculations because they were used as reference data to determine regional strontium ranges.

At least 23% (95% CI: 17–30%) of all individuals in our sample set are of non-local origin. 6% (95% CI: 3–10%) are individuals that show strontium isotope ratios within the range of South Bavaria, whereas 17% (95% CI: 12–23%) are non-locals that show strontium ratios which are not found in the North Alpine foreland of South Bavaria.

Figure S3.4.1 shows isotopic ratios, ^87^Sr/^86^Sr in enamel (left), δ^13^C (middle) and δ^15^N (right) in bone collagen of non-locals and “locals” as revealed by Strontium isotope analysis.

**
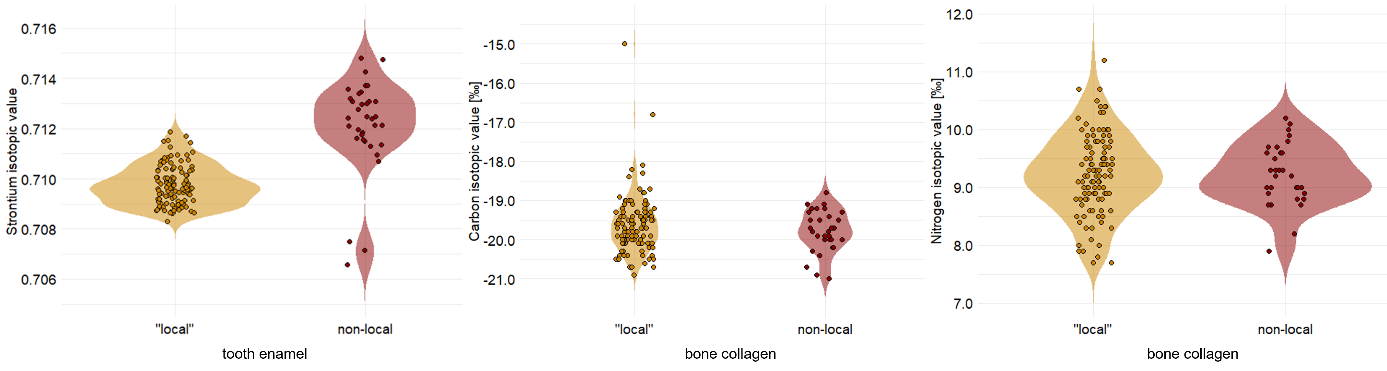
**

**Fig S3.4.1: Isotopic ratio range of non-locals and “locals” in Bavaria around 500 AD.**

Left: ^87^Sr/^86^Sr. Middle: δ^13^C. Right: δ^15^N. Ratios of “locals” are illustrated in orange, ratios of non-locals are illustrated in red (illustration/calculation: R Studio package ggplot2 [56-57]).

### S3.4.1 Provenance vs. sex

To investigate differences between sexes data of individuals determined as rather male or male are grouped and compared to ratios of rather female and female individuals. Individuals with indetermined sex are excluded.

We found no significant correlation between non-local provenance and sex, although we identified more female non-locals, but women are overrepresented in the sample set (Table S3.4.1.1, Table S3.4.1.2).

Table S3.4.1.1: Crosstabulation provenance (“local”=individuals who show strontium ratios inside the range determined for respective regions, non-local=individuals who show strontium ratios outside the range determined for respective regions) vs. sex (male=male and rather male individuals, female=female and rather female individuals).

|  | | | Sex | | Total |
| --- | --- | --- | --- | --- | --- |
|  |  |  | male | female |  |
| Provenance | "local" | Count | 45 | 70 | 115 |
|  |  | Expected Count | 45.2 | 69.8 | 115.0 |
|  |  | % within Provenance | 39.1% | 60.9% | 100.0% |
|  |  | % within Sex | 76.3% | 76.9% | 76.7% |
|  |  | 95% CI | 66.1-86.4% | 67.8-85.7% |  |
|  | non-local | Count | 14 | 21 | 35 |
|  |  | Expected Count | 13.8 | 21.2 | 35.0 |
|  |  | % within Provenance | 40.0% | 60.0% | 100.0% |
|  |  | % within Sex | 23.7% | 23.1% | 23.3% |
|  |  | 95% CI | 13.6-33.9% | 14.3-32.2% |  |
| Total | | Count | 59 | 91 | 150 |
|  |  | Expected Count | 59.0 | 91.0 | 150.0 |
|  |  | % within Provenance | 39.3% | 60.7% | 100.0% |
|  |  | % within Sex | 100.0% | 100.0% | 100.0% |

Table S3.4.1.2: Pearson Chi-Square Test for provenance (“local”=individuals who show strontium ratios inside the range determined for respective regions, non-local=individuals who show strontium ratios outside the range determined for respective regions) vs. sex (male=male and rather male individuals, female=female and rather female individuals).

|  | Value | df | Asymptotic Significance (2-sided) | Exact Sig. (2-sided) | Exact Sig. (1-sided) |
| --- | --- | --- | --- | --- | --- |
| Pearson Chi-Square | 0.009^a^ | 1 | 0.927 |  |  |
| Continuity Correction^b^ | 0.000 | 1 | 1.000 |  |  |
| Likelihood Ratio | 0.008 | 1 | 0.927 |  |  |
| Fisher's Exact Test |  |  |  | 1.000 | 0.539 |
| Linear-by-Linear Association | 0.008 | 1 | 0.927 |  |  |
| N of Valid Cases | 150 |  |  |  |  |
| a. 0 cells (0.0%) have expected count less than 5. The minimum expected count is 13.77.  b. Computed only for a 2x2 table | | | | | |

We also found no significant difference between sexes if we only differentiate between individuals who show strontium ratios which are found in the Northern pre-Alpine foothills of South Bavaria (Bavarian ^87^Sr/^86^Sr) or not (Tab S3.4.1.3, Tab S3.4.1.4).

Table 3.4.1.3: Crosstabulation Bavarian ^87^Sr/^86^Sr (yes=individuals who show strontium ratios inside the range determined for South Bavaria, no=individuals who show strontium ratios outside the range determined for South Bavaria) vs. sex (male=male and rather male individuals, female=female and rather female individuals).

|  | | | Sex | | Total |
| --- | --- | --- | --- | --- | --- |
|  |  |  | male | female |  |
| Bavarian ^87^Sr/^86^Sr | yes | Count | 50 | 74 | 124 |
|  |  | Expected Count | 48.8 | 75.2 | 124.0 |
|  |  | % within Bavarian ^87^Sr/^86^Sr | 40.3% | 59.7% | 100.0% |
|  |  | % within Sex | 84.7% | 81.3% | 82.7% |
|  |  | 95% CI | 74.6-93.2% | 72.5-89.0% |  |
|  | no | Count | 9 | 17 | 26 |
|  |  | Expected Count | 10.2 | 15.8 | 26.0 |
|  |  | % within Bavarian ^87^Sr/^86^Sr | 34.6% | 65.4% | 100.0% |
|  |  | % within Sex | 15.3% | 18.7% | 17.3% |
|  |  | 95% CI | 6.8-25.4% | 11.0-27.5 |  |
| Total | | Count | 59 | 91 | 150 |
|  |  | Expected Count | 59.0 | 91.0 | 150.0 |
|  |  | % within Bavarian Sr | 39.3% | 60.7% | 100.0% |
|  |  | % within Sex | 100.0% | 100.0% | 100.0% |

Table 3.4.1.4: Pearson Chi-Square Test for Bavarian ^87^Sr/^86^Sr (yes=individuals who show strontium ratios inside the range determined for South Bavaria, no=individuals who show strontium ratios outside the range determined for South Bavaria) vs. sex (male=male and rather male individuals, female=female and rather female individuals).

|  | Value | df | Asymptotic Significance (2-sided) | Exact Sig. (2-sided) | Exact Sig. (1-sided) |
| --- | --- | --- | --- | --- | --- |
| Pearson Chi-Square | 0.293^a^ | 1 | 0.588 |  |  |
| Continuity Correction^b^ | 0.103 | 1 | 0.748 |  |  |
| Likelihood Ratio | 0.297 | 1 | 0.586 |  |  |
| Fisher's Exact Test |  |  |  | 0.663 | 0.378 |
| Linear-by-Linear Association | 0.291 | 1 | 0.589 |  |  |
| N of Valid Cases | 150 |  |  |  |  |
| a. 0 cells (0.0%) have expected count less than 5. The minimum expected count is 10.23.  b. Computed only for a 2x2 table | | | | | |

Moreover, we observe no difference between sexes if we differentiate between non-locals that show strontium ratios which are not found in the area of South Bavaria and most likely migrated to the region from outside the area and individuals who show strontium ratios that do not fit into determined ranges of respective regions but are found in other regions in South Bavaria (their origin might be found inside or outside the Northern Alpine foreland of Bavaria) (Tab S3.4.1.5, Tab S3.4.1.6).

Table3.4.1.5: Crosstabulation provenance (“local”=individuals who show strontium ratios inside ranges determined for respective regions; non-local, not Bavarian=individuals who show strontium ratios outside ranges determined for respective regions and outside the range of South Bavaria; non-local, possible Bavarian= individuals who show strontium ratios outside ranges determined for respective regions but inside ranges determined at other regions in South Bavaria) vs. sex (male=male and rather male individuals, female=female and rather female individuals).

|  | | | Sex | | Total |
| --- | --- | --- | --- | --- | --- |
|  |  |  | male | female |  |
| Provenance | "local" | Count | 45 | 70 | 115 |
|  |  | Expected Count | 45.2 | 69.8 | 115.0 |
|  |  | % within Provenance | 39.1% | 60.9% | 100.0% |
|  |  | % within Sex | 76.3% | 76.9% | 76.7% |
|  |  | 95% CI | 64.4-86.4% | 68.1-84.6% |  |
|  | non-local, not Bavarian | Count | 9 | 17 | 26 |
|  |  | Expected Count | 10.2 | 15.8 | 26.0 |
|  |  | % within Provenance | 34.6% | 65.4% | 100.0% |
|  |  | % within Sex | 15.3% | 18.7% | 17.3% |
|  |  | 95% CI | 6.8-25.4% | 11.0-26.4% |  |
|  | non-local, possible Bavarian | Count | 5 | 4 | 9 |
|  |  | Expected Count | 3.5 | 5.5 | 9.0 |
|  |  | % within Provenance | 55.6% | 44.4% | 100.0% |
|  |  | % within Sex | 8.5% | 4.4% | 6.0% |
|  |  | 95% CI | 1.7-16.9% | 1.1-8.8% |  |
| Total | | Count | 59 | 91 | 150 |
|  |  | Expected Count | 59.0 | 91.0 | 150.0 |
|  |  | % within Provenance | 39.3% | 60.7% | 100.0% |
|  |  | % within Sex | 100.0% | 100.0% | 100.0% |

Table S3.4.1.6: Pearson Chi-Square Test for provenance (“local”=individuals who show strontium ratios inside ranges determined for respective regions; non-local, not Bavarian=individuals who show strontium ratios outside ranges determined for respective regions and outside the range of South Bavaria; non-local, possible Bavarian= individuals who show strontium ratios outside ranges determined for respective regions but inside ranges determined at other regions in South Bavaria) vs. sex (male=male and rather male individuals, female=female and rather female individuals).

|  | Value | df | Asymptotic Significance (2-sided) |
| --- | --- | --- | --- |
| Pearson Chi-Square | 1.237^a^ | 2 | 0.539 |
| Likelihood Ratio | 1.212 | 2 | 0.545 |
| Linear-by-Linear Association | 0.243 | 1 | 0.622 |
| N of Valid Cases | 150 |  |  |
| 1 cells (16.7%) have expected count less than 5. The minimum expected count is 3.54. | | | |

### S3.4.2 Provenance vs. settlement zone

To investigate differences between settlement zones, data of excavation sites found in the border region (Burgweinting (BW), Irlmauth (IRM) and Straubing-Bajuwarenstraße (STB)) are grouped and compared to sites in the hinterland (Altenerding (AED), Munich-Perlach (PEL), Unterhaching (UTH)). The single find of Alteglofsheim (AEH) is excluded.

A significant difference between settlement zones is found. A higher frequency of non-locals is found in the hinterland (Table S3.4.2.1, Table S3.4.2.2).

Table S3.4.2.1: Crosstabulation provenance (“local”=individuals who show strontium ratios inside the range determined for respective regions, non-local=individuals who show strontium ratios outside the range determined for respective regions) vs. settlement zone (border=individuals from BW, IRM and STB), hinterland=individuals from AED, PEL and UTH)

|  | | | Settlement zone | | Total |
| --- | --- | --- | --- | --- | --- |
|  |  |  | border | hinterland |  |
| Provenance | "local" | Count | 63 | 51 | 114 |
|  |  | Expected Count | 56.6 | 57.4 | 114.0 |
|  |  | % within Provenance | 55.3% | 44.7% | 100.0% |
|  |  | % within Settlement zone | 85.1% | 68.0% | 76.5% |
|  |  | 95% CI | 76.4-93.2% | 57.3-78.7% |  |
|  | non-local | Count | 11 | 24 | 35 |
|  |  | Expected Count | 17.4 | 17.6 | 35.0 |
|  |  | % within Provenance | 31.4% | 68.6% | 100.0% |
|  |  | % within Settlement zone | 14.9% | 32.0% | 23.5% |
|  |  | 95% CI | 6.9-23.6% | 21.9-43.1% |  |
| Total | | Count | 74 | 75 | 149 |
|  |  | Expected Count | 74.0 | 75.0 | 149.0 |
|  |  | % within Provenance | 49.7% | 50.3% | 100.0% |
|  |  | % within Settlement zone | 100.0% | 100.0% | 100.0% |

Table S3.4.2.2: Pearson Chi-Square Test for provenance (“local”=individuals who show strontium ratios inside the range determined for respective regions, non-local=individuals who show strontium ratios outside the range determined for respective regions) vs. settlement zone (border=individuals from BW, IRM and STB), hinterland=individuals from AED, PEL and UTH)

|  | Value | df | Asymptotic Significance (2-sided) | Exact Sig. (2-sided) | Exact Sig. (1-sided) |
| --- | --- | --- | --- | --- | --- |
| Pearson Chi-Square | 6.085^a^ | 1 | 0.014 |  |  |
| Continuity Correction^b^ | 5.169 | 1 | 0.023 |  |  |
| Likelihood Ratio | 6.205 | 1 | 0.013 |  |  |
| Fisher's Exact Test |  |  |  | 0.020 | 0.011 |
| Linear-by-Linear Association | 6.044 | 1 | 0.014 |  |  |
| N of Valid Cases | 149 |  |  |  |  |
| a. 0 cells (0.0%) have expected count less than 5. The minimum expected count is 17.38.  b. Computed only for a 2x2 table | | | | | |

No significant difference is found between settlement zones if we only differentiate between individuals who show strontium ratios which are found in the Northern pre-Alpine foothills of South Bavaria (Bavarian ^87^Sr/^86^Sr) or not (Tab S3.4.2.3, Tab S3.4.2.4).

Table S3.4.2.3: Crosstabulation Bavarian ^87^Sr/^86^Sr (yes=individuals who show strontium ratios inside the range determined for South Bavaria, no=individuals who show strontium ratios outside the range determined for South Bavaria) vs. settlement zone (border=individuals from BW, IRM and STB), hinterland=individuals from AED, PEL and UTH).

|  | | | Settlement zone | | Total |
| --- | --- | --- | --- | --- | --- |
|  |  |  | border | hinterland |  |
| Bavarian ^87^Sr/^86^Sr | yes | Count | 64 | 59 | 123 |
|  |  | Expected Count | 61.1 | 61.9 | 123.0 |
|  |  | % within Bavarian ^87^Sr/^86^Sr | 52.0% | 48.0% | 100.0% |
|  |  | % within Settlement zone | 86.5% | 78.7% | 82.6% |
|  |  | 95% CI | 78.1-93.3% | 69.3-88.0% |  |
|  | no | Count | 10 | 16 | 26 |
|  |  | Expected Count | 12.9 | 13.1 | 26.0 |
|  |  | % within Bavarian ^87^Sr/^86^Sr | 38.5% | 61.5% | 100.0% |
|  |  | % within Settlement zone | 13.5% | 21.3% | 17.4% |
|  |  | 95% CI | 6.7-21.9% | 12.0-30.7% |  |
| Total | | Count | 74 | 75 | 149 |
|  |  | Expected Count | 74.0 | 75.0 | 149.0 |
|  |  | % within Bavarian ^87^Sr/^86^Sr | 49.7% | 50.3% | 100.0% |
|  |  | % within Settlement zone | 100.0% | 100.0% | 100.0% |

Table S3.4.2.4: Pearson Chi-Square Test for Bavarian ^87^Sr/^86^Sr (yes=individuals who show strontium ratios inside the range determined for South Bavaria, no=individuals who show strontium ratios outside the range determined for South Bavaria) vs. settlement zone (border=individuals from BW, IRM and STB), hinterland=individuals from AED, PEL and UTH).

|  | Value | df | Asymptotic Significance (2-sided) | Exact Sig. (2-sided) | Exact Sig. (1-sided) |
| --- | --- | --- | --- | --- | --- |
| Pearson Chi-Square | 1.581^a^ | 1 | 0.209 |  |  |
| Continuity Correction^b^ | 1.085 | 1 | 0.298 |  |  |
| Likelihood Ratio | 1.594 | 1 | 0.207 |  |  |
| Fisher's Exact Test |  |  |  | 0.281 | 0.149 |
| Linear-by-Linear Association | 1.571 | 1 | 0.210 |  |  |
| N of Valid Cases | 149 |  |  |  |  |
| a. 0 cells (0.0%) have expected count less than 5. The minimum expected count is 12.91.  b. Computed only for a 2x2 table | | | | | |

However, we observe a difference between settlement zones if we differentiate between non-locals that show strontium ratios which are not found in the area of South Bavaria and most likely migrated to the region from outside the area and individuals who show strontium ratios that do not fit into determined ranges of respective regions but are found in other regions in South Bavaria (their origin might be found inside or outside the Northern Alpine foreland of Bavaria) (Tab S3.4.2.5, Tab S3.4.2.6).

Table S3.4.2.5: Crosstabulation provenance (“local”=individuals who show strontium ratios inside ranges determined for respective regions; non-local, not Bavarian=individuals who show strontium ratios outside ranges determined for respective regions and outside the range of South Bavaria; non-local, possible Bavarian= individuals who show strontium ratios outside ranges determined for respective regions but inside ranges determined at other regions in South Bavaria) vs. settlement zone (border=individuals from BW, IRM and STB), hinterland=individuals from AED, PEL and UTH).

|  | | | Settlement zone | | Total |
| --- | --- | --- | --- | --- | --- |
|  |  |  | border | hinterland |  |
| Provenance | "local" | Count | 63 | 51 | 114 |
|  |  | Expected Count | 56.6 | 57.4 | 114.0 |
|  |  | % within Provenance | 55.3% | 44.7% | 100.0% |
|  |  | % within Settlement zone | 85.1% | 68.0% | 76.5% |
|  |  | 95% CI | 76.4-93.2% | 57.3-78.7% |  |
|  |  | Adjusted Residual | 2.5 | -2.5 |  |
|  |  | Pearson Chi-Square | 0.0124 | 0.0124 |  |
|  | Non-local, not Bavarian | Count | 10 | 16 | 26 |
|  |  | Expected Count | 12.9 | 13.1 | 26.0 |
|  |  | % within Provenance | 38.5% | 61.5% | 100.0% |
|  |  | % within Settlement zone | 13.5% | 21.3% | 17.4% |
|  |  | 95% CI | 6.7-21.9% | 13.3-30.7% |  |
|  |  | Adjusted Residual | -1.3 | 1.3 |  |
|  |  | Pearson Chi-Square | 0.1936 | 0.1936 |  |
|  | Non-local, possible Bavarian | Count | 1 | 8 | 9 |
|  |  | Expected Count | 4.5 | 4.5 | 9.0 |
|  |  | % within Provenance | 11.1% | 88.9% | 100.0% |
|  |  | % within Settlement zone | 1.4% | 10.7% | 6.0% |
|  |  | 95% CI | 0.0-4.1% | 4.0-17.3% |  |
|  |  | Adjusted Residual | -2.4 | 2.4 |  |
|  |  | Pearson Chi-Square | 0.0164* | 0.0164* |  |
| Total | | Count | 74 | 75 | 149 |
|  |  | Expected Count | 74.0 | 75.0 | 149.0 |
|  |  | % within Provenance | 49.7% | 50.3% | 100.0% |
|  |  | % within Settlement zone | 100.0% | 100.0% | 100.0% |
| * significant after Bonferroni correction (p<0.0167) | | | | | |

Table S3.4.2.6:Pearson Chi-Square Test for provenance (“local”=individuals who show strontium ratios inside ranges determined for respective regions; non-local, not Bavarian=individuals who show strontium ratios outside ranges determined for respective regions and outside the range of South Bavaria; non-local, possible Bavarian= individuals who show strontium ratios outside ranges determined for respective regions but inside ranges determined at other regions in South Bavaria) vs. settlement zone (border=individuals from BW, IRM and STB), hinterland=individuals from AED, PEL and UTH).

|  | Value | df | Asymptotic Significance (2-sided) |
| --- | --- | --- | --- |
| Pearson Chi-Square | 8.086^a^ | 2 | 0.018 |
| Likelihood Ratio | 8.854 | 2 | 0.012 |
| Linear-by-Linear Association | 7.870 | 1 | 0.005 |
| N of Valid Cases | 149 |  |  |
| a. 2 cells (33.3%) have expected count less than 5. The minimum expected count is 4.47. | | | |

### S3.4.3 Provenance vs. excavation sites

To investigate differences between excavation sites, data of six investigated burial grounds are compared. The single find of Alteglofsheim (AEH) is excluded.

A significant difference in the number of non-locals between excavation sites is found (Table S3.4.3.2). Pairwise comparisons (Table S3.4.3.1) of sites revealed a significant higher number of migrants in Unterhaching (UTH) than expected.

Table S3.4.3.1: Crosstabulation provenance (“local”=individuals who show strontium ratios inside the range determined for respective regions, non-local=individuals who show strontium ratios outside the range determined for respective regions) vs. excavation site (Altenerding, Straubing-Bajuwarenstraße, Irlmauth, Burgweinting, Munich-Perlach, Unterhaching).

|  | | | Provenance | | Total |
| --- | --- | --- | --- | --- | --- |
|  |  |  | "local" | non-local |  |
| Excavation | Altenerding | Count | 29 | 14 | 43 |
|  |  | Expected Count | 32.9 | 10.1 | 43.0 |
|  |  | % within Excavation | 67.4% | 32.6% | 100.0% |
|  |  | 95% CI | 53.5-82.0% | 18.0-46.5% |  |
|  |  | % within Provenance | 25.4% | 40.0% | 28.9% |
|  |  | Adjusted Residual | -1.7 | 1.7 |  |
|  |  | Pearson Chi Square | 0.0891 | 0.0891 |  |
|  | Straubing-Bajuwarenstraße | Count | 29 | 2 | 31 |
|  |  | Expected Count | 23.7 | 7.3 | 31.0 |
|  |  | % within Excavation | 93.5% | 6.5% | 100.0% |
|  |  | 95% CI | 83.9-100.0% | 0.0-16.1% |  |
|  |  | % within Provenance | 25.4% | 5.7% | 20.8% |
|  |  | Adjusted Residual | 2.5 | -2.5 |  |
|  |  | Pearson Chi Square | 0.0124 | 0.0124 |  |
|  | Irlmauth | Count | 12 | 2 | 14 |
|  |  | Expected Count | 10.7 | 3.3 | 14.0 |
|  |  | % within Excavation | 85.7% | 14.3% | 100.0% |
|  |  | 95% CI | 66.7-100.0% | 0.0-33.3% |  |
|  |  | % within Provenance | 10.5% | 5.7% | 9.4% |
|  |  | Adjusted Residual | 0.9 | -0.9 |  |
|  |  | Pearson Chi Square | 0.3681 | 0.3681 |  |
|  | Burgweinting | Count | 22 | 7 | 29 |
|  |  | Expected Count | 22.2 | 6.8 | 29.0 |
|  |  | % within Excavation | 75.9% | 24.1% | 100.0% |
|  |  | 95% CI | 60.0-90.3% | 9.7-40.0% |  |
|  |  | % within Provenance | 19.3% | 20.0% | 19.5% |
|  |  | Adjusted Residual | -0.1 | 0.1 |  |
|  |  | Pearson Chi Square | 0.9203 | 0.9203 |  |
|  | Munich-Perlach | Count | 20 | 3 | 23 |
|  |  | Expected Count | 17.6 | 5.4 | 23.0 |
|  |  | % within Excavation | 87.0% | 13.0% | 100.0% |
|  |  | 95% CI | 69.6-100.0% | 0.0-30.4% |  |
|  |  | % within Provenance | 17.5% | 8.6% | 15.4% |
|  |  | Adjusted Residual | 1.3 | -1.3 |  |
|  |  | Pearson Chi Square | 0.1936 | 0.1936 |  |
|  | Unterhaching | Count | 2 | 7 | 9 |
|  |  | Expected Count | 6.9 | 2.1 | 9.0 |
|  |  | % within Excavation | 22.2% | 77.8% | 100.0% |
|  |  | 95% CI | 0.0-55.6% | 44.4-100.0% |  |
|  |  | % within Provenance | 1.8% | 20.0% | 6.0% |
|  |  | Adjusted Residual | -4.0 | 4.0 |  |
|  |  | Pearson Chi Square | <0.0001* | <0.0001* |  |
| Total | | Count | 114 | 35 | 149 |
|  |  | Expected Count | 114.0 | 35.0 | 149.0 |
|  |  | % within Excavation | 76.5% | 23.5% | 100.0% |
|  |  | % within Provenance | 100.0% | 100.0% | 100.0% |
| * significant after Bonferroni correction (p<0.0083) | | | | | |

Table S3.4.3.2: Pearson Chi-Square Test for provenance (“local”=individuals who show strontium ratios inside the range determined for respective regions, non-local=individuals who show strontium ratios outside the range determined for respective regions) vs. excavation site (Altenerding, Straubing-Bajuwarenstraße, Irlmauth, Burgweinting, Munich-Perlach, Unterhaching).

|  | Value | df | Asymptotic Significance (2-sided) |
| --- | --- | --- | --- |
| Pearson Chi-Square | 23.797^a^ | 5 | <0.001 |
| Likelihood Ratio | 22.466 | 5 | <0.001 |
| Linear-by-Linear Association | 1.066 | 1 | 0.302 |
| N of Valid Cases | 149 |  |  |
| a. 2 cells (16.7%) have expected count less than 5. The minimum expected count is 2.11. | | | |

No significant difference is found between excavation sites if we only differentiate between individuals who show strontium ratios which are found in the Northern pre-Alpine foothills of South Bavaria (Bavarian ^87^Sr/^86^Sr) or not (Tab S3.4.3.3, Tab S3.4.3.4).

Table S3.4.3.3: Crosstabulation Bavarian ^87^Sr/^86^Sr (yes=individuals who show strontium ratios inside the range determined for South Bavaria, no=individuals who show strontium ratios outside the range determined for South Bavaria) vs. excavation site (Altenerding, Straubing-Bajuwarenstraße, Irlmauth, Burgweinting, Munich-Perlach, Unterhaching).

|  | | | Bavarian ^87^Sr/^86^Sr | | Total |
| --- | --- | --- | --- | --- | --- |
|  |  |  | yes | no |  |
| Excavation | Altenerding | Count | 33 | 10 | 43 |
|  |  | Expected Count | 35.5 | 7.5 | 43.0 |
|  |  | % within Excavation | 76.7% | 23.3% | 100.0% |
|  |  | 95% CI | 62.8-88.4% | 11.6-37.2% |  |
|  |  | % within Bavarian ^87^Sr/^86^Sr | 26.8% | 38.5% | 28.9% |
|  | Straubing-Bajuwarenstraße | Count | 30 | 1 | 31 |
|  |  | Expected Count | 25.6 | 5.4 | 31.0 |
|  |  | % within Excavation | 96.8% | 3.2% | 100.0% |
|  |  | 95% CI | 90.3-100.0% | 0.0-9.7% |  |
|  |  | % within Bavarian ^87^Sr/^86^Sr | 24.4% | 3.8% | 20.8% |
|  | Irlmauth | Count | 12 | 2 | 14 |
|  |  | Expected Count | 11.6 | 2.4 | 14.0 |
|  |  | % within Excavation | 85.7% | 14.3% | 100.0% |
|  |  | 95% CI | 64.3-100.0% | 0.0-35.7% |  |
|  |  | % within Bavarian ^87^Sr/^86^Sr | 9.8% | 7.7% | 9.4% |
|  | Burgweinting | Count | 22 | 7 | 29 |
|  |  | Expected Count | 23.9 | 5.1 | 29.0 |
|  |  | % within Excavation | 75.9% | 24.1% | 100.0% |
|  |  | 95% CI | 58.6-89.7% | 10.3-41.4% |  |
|  |  | % within Bavarian ^87^Sr/^86^Sr | 17.9% | 26.9% | 19.5% |
|  | Munich-Perlach | Count | 20 | 3 | 23 |
|  |  | Expected Count | 19.0 | 4.0 | 23.0 |
|  |  | % within Excavation | 87.0% | 13.0% | 100.0% |
|  |  | 95% CI | 73.9-100.0% | 0.0-26.1% |  |
|  |  | % within Bavarian ^87^Sr/^86^Sr | 16.3% | 11.5% | 15.4% |
|  | Unterhaching | Count | 6 | 3 | 9 |
|  |  | Expected Count | 7.4 | 1.6 | 9.0 |
|  |  | % within Excavation | 66.7% | 33.3% | 100.0% |
|  |  | 95% CI | 33.3-100.0% | 0.0-66.7% |  |
|  |  | % within Bavarian ^87^Sr/^86^Sr | 4.9% | 11.5% | 6.0% |
| Total | | Count | 123 | 26 | 149 |
|  |  | Expected Count | 123.0 | 26.0 | 149.0 |
|  |  | % within Excavation | 82.6% | 17.4% | 100.0% |
|  |  | % within Bavarian ^87^Sr/^86^Sr | 100.0% | 100.0% | 100.0% |

Table S3.4.3.4: Pearson Chi-Square Test for Bavarian ^87^Sr/^86^Sr (yes=individuals who show strontium ratios inside the range determined for South Bavaria, no=individuals who show strontium ratios outside the range determined for South Bavaria) vs. excavation site (Altenerding, Straubing-Bajuwarenstraße, Irlmauth, Burgweinting, Munich-Perlach, Unterhaching).

|  | Value | df | Asymptotic Significance (2-sided) |
| --- | --- | --- | --- |
| Pearson Chi-Square | 8.245^a^ | 5 | 0.143 |
| Likelihood Ratio | 9.673 | 5 | 0.085 |
| Linear-by-Linear Association | 0.169 | 1 | 0.681 |
| N of Valid Cases | 149 |  |  |
| a. 3 cells (25.0%) have expected count less than 5. The minimum expected count is 1.57. | | | |

However, we observe a difference between excavation sites if we differentiate between non-locals that show strontium ratios which are not found in the area of South Bavaria and most likely migrated to the region from outside the area and individuals who show strontium ratios that do not fit into determined ranges of respective regions but are found in other regions in South Bavaria (their origin might be found inside or outside the Northern Alpine foreland of Bavaria) (Tab S3.4.3.5, Tab S3.4.3.6).

Table S3.4.3.5: Crosstabulation provenance (“local”=individuals who show strontium ratios inside ranges determined for respective regions; non-local, not Bavarian=individuals who show strontium ratios outside ranges determined for respective regions and outside the range of South Bavaria; non-local, possible Bavarian= individuals who show strontium ratios outside ranges determined for respective regions but inside ranges determined at other regions in South Bavaria) vs. excavation site (Altenerding, Straubing-Bajuwarenstraße, Irlmauth, Burgweinting, Munich-Perlach, Unterhaching).

|  | | | Provenance | | | Total |
| --- | --- | --- | --- | --- | --- | --- |
|  |  |  | "local" | non-local, not Bavarian | non-local, possible Bavarian |  |
| Excavation | Altenerding | Count | 29 | 10 | 4 | 43 |
|  |  | Expected Count | 32.9 | 7.5 | 2.6 | 43.0 |
|  |  | % within Excavation | 67.4% | 23.3% | 9.3% | 100.0% |
|  |  | 95% CI | 53.5-81.4% | 11.6-37.2% | 2.3-18.6% |  |
|  |  | % within Provenance | 25.4% | 38.5% | 44.4% | 28.9% |
|  |  | Adjusted Residual | -1.7 | 1.2 | 1.1 |  |
|  |  | Pearson Chi Square | 0.0891 | 0.2301 | 0.2713 |  |
|  | Straubing-Bajuwarenstraße | Count | 29 | 1 | 1 | 31 |
|  |  | Expected Count | 23.7 | 5.4 | 1.9 | 31.0 |
|  |  | % within Excavation | 93.5% | 3.2% | 3.2% | 100.0% |
|  |  | 95% CI | 83.9-100.0% | 0.0-9.7% | 0.0-9.7% |  |
|  |  | % within Provenance | 25.4% | 3.8% | 11.1% | 20.8% |
|  |  | Adjusted Residual | 2.5 | -2.3 | -0.7 |  |
|  |  | Pearson Chi Square | 0.01241 | 0.0215 | 0.4839 |  |
|  | Irlmauth | Count | 12 | 2 | 0 | 14 |
|  |  | Expected Count | 10.7 | 2.4 | .8 | 14.0 |
|  |  | % within Excavation | 85.7% | 14.3% | 0.0% | 100.0% |
|  |  | 95% CI | 64.3-100.0% | 0.0-35.7% |  |  |
|  |  | % within Provenance | 10.5% | 7.7% | 0.0% | 9.4% |
|  |  | Adjusted Residual | 0.9 | -0.3 | -1.0 |  |
|  |  | Pearson Chi Square | 0.3681 | 0.7642 | 0.3173 |  |
|  | Burgweinting | Count | 22 | 7 | 0 | 29 |
|  |  | Expected Count | 22.2 | 5.1 | 1.8 | 29.0 |
|  |  | % within Excavation | 75.9% | 24.1% | 0.0% | 100.0% |
|  |  | 95% CI | 58.6-89.7% | 10.3-41.4% |  |  |
|  |  | % within Provenance | 19.3% | 26.9% | 0.0% | 19.5% |
|  |  | Adjusted Residual | -0.1 | 1.1 | -1.5 |  |
|  |  | Pearson Chi Square | 0.9203 | 0.2713 | 0.1336 |  |
|  | Munich-Perlach | Count | 20 | 3 | 0 | 23 |
|  |  | Expected Count | 17.6 | 4.0 | 1.4 | 23.0 |
|  |  | % within Excavation | 87.0% | 13.0% | 0.0% | 100.0% |
|  |  | 95% CI | 73.9-100% | 0.0-26.1% |  |  |
|  |  | % within Provenance | 17.5% | 11.5% | 0.0% | 15.4% |
|  |  | Adjusted Residual | 1.3 | -0.6 | -1.3 |  |
|  |  | Pearson Chi Square | 0.1936 | 0.5485 | 0.1936 |  |
|  | Unterhaching | Count | 2 | 3 | 4 | 9 |
|  |  | Expected Count | 6.9 | 1.6 | 0.5 | 9.0 |
|  |  | % within Excavation | 22.2% | 33.3% | 44.4% | 100.0% |
|  |  | 95% CI | 0.0-55.6% | 0.0-66.7% | 11.1-77.8% |  |
|  |  | % within Provenance | 1.8% | 11.5% | 44.4% | 6.0% |
|  |  | Adjusted Residual | -4.0 | 1.3 | 5.0 |  |
|  |  | Pearson Chi Square | <0.0001* | 0.1936 | <0.0001* |  |
| Total | | Count | 114 | 26 | 9 | 149 |
|  |  | Expected Count | 114.0 | 26.0 | 9.0 | 149.0 |
|  |  | % within Excavation | 76.5% | 17.4% | 6.0% | 100.0% |
|  |  | % within Provenance | 100.0% | 100.0% | 100.0% | 100.0% |
| * significant after Bonferroni correction (p<0.0028) | | | | | | |

Table S3.4.3.6: Pearson Chi-Square Test for provenance (“local”=individuals who show strontium ratios inside ranges determined for respective regions; non-local, not Bavarian=individuals who show strontium ratios outside ranges determined for respective regions and outside the range of South Bavaria; non-local, possible Bavarian= individuals who show strontium ratios outside ranges determined for respective regions but inside ranges determined at other regions in South Bavaria) vs. excavation site (Altenerding, Straubing-Bajuwarenstraße, Irlmauth, Burgweinting, Munich-Perlach, Unterhaching).

|  | Value | df | Asymptotic Significance (2-sided) |
| --- | --- | --- | --- |
| Pearson Chi-Square | 39.522^a^ | 10 | <0.001 |
| Likelihood Ratio | 33.285 | 10 | <0.001 |
| Linear-by-Linear Association | 1.574 | 1 | 0.210 |
| N of Valid Cases | 149 |  |  |
| a. 9 cells (50.0%) have expected count less than 5. The minimum expected count is 0.54. | | | |

## S3.5 Human migration in Bavaria over time periods

Looking at the total sample (N=519) the average proportion of migrants is 15% (95% CI: 11-17%).

The Chi-square test (Table S3.5.2) shows that there is a significant statistical relationship mainly due to the fact that more non-local than expected are detected in the period around 500 AD (Tab S3.5.1).

Table S3.5.1: Crosstabulation provenance (“local”=individuals who show strontium ratios inside the range determined for the Northern pre-Alpine foreland in South Bavaria, non-local= individuals who show strontium ratios outside the range determined for the Northern pre-Alpine foreland in South Bavaria) vs. time period (EN=Early Neolithics, EMA=Early Middle Ages, LN=Late Neolithics, EBA= Early Bronze Age, LA=Late Antique, 500AD=around 500 AD, IA= Iron Age). 95% CI = 95% Confidence interval.

|  | | | Provenance | | Total |
| --- | --- | --- | --- | --- | --- |
|  |  |  | local | non-local |  |
| Dating | EN | Count | 77 | 5 | 82 |
|  |  | Expected Count | 70.2 | 11.8 | 82,0 |
|  |  | % within Dating | 93.9% | 6.1% | 100.0% |
|  |  | 95% CI | 89.0-98.8% | 1.2-11.0% |  |
|  |  | % within Provenance | 17.3% | 6.7% | 15.8% |
|  |  | Adjusted Residual | 2.3445 | -2.3445 |  |
|  |  | Pearson Chi-Square | 0.0191 | 0.0191 |  |
|  | EMA | Count | 35 | 2 | 37 |
|  |  | Expected Count | 31.7 | 5.3 | 37.0 |
|  |  | % within Dating | 94.6% | 5.4% | 100.0% |
|  |  | 95% CI | 86.5-100.0% | 0.0-13.5% |  |
|  |  | % within Provenance | 7.9% | 2.7% | 7.1% |
|  |  | Adjusted Residual | 1.6238 | -1.6238 |  |
|  |  | Pearson Chi-Square | 0.1044 | 0.1044 |  |
|  | LN | Count | 88 | 12 | 100 |
|  |  | Expected Count | 85.5 | 14.5 | 100.0 |
|  |  | % within Dating | 88.0% | 12.0% | 100,0% |
|  |  | 95% CI | 81.0-94.0% | 6.0-19.0% |  |
|  |  | % within Provenance | 19.8% | 16.0% | 19.3% |
|  |  | Adjusted Residual | 0.7758 | -0.7758 |  |
|  |  | Pearson Chi-Square | 0.4379 | 0.4379 |  |
|  | EBA | Count | 49 | 10 | 59 |
|  |  | Expected Count | 50.5 | 8.5 | 59.0 |
|  |  | % within Dating | 83.1% | 16.9% | 100.0% |
|  |  | 95% CI | 72.9-91.5% | 8.5-27.1% |  |
|  |  | % within Provenance | 11.0% | 13.3% | 11.4% |
|  |  | Adjusted Residual | -0.5797 | 0.5797 |  |
|  |  | Pearson Chi-Square | 0.5621 | 0.5621 |  |
|  | LA | Count | 94 | 15 | 109 |
|  |  | Expected Count | 93.2 | 15.8 | 109.0 |
|  |  | % within Dating | 86.2% | 13.8% | 100.0% |
|  |  | 95% CI | 78.9-92.7% | 7.3-21.1% |  |
|  |  | % within Provenance | 21.2% | 20.0% | 21.0% |
|  |  | Adjusted Residual | 0.2303 | -0.2303 |  |
|  |  | Pearson Chi-Square | 0.8178 | 0.8178 |  |
|  | 500AD | Count | 83 | 25 | 108 |
|  |  | Expected Count | 92.4 | 15.6 | 108.0 |
|  |  | % within Dating | 76.9% | 23.1% | 100.0% |
|  |  | 95% CI | 69.4-85.2% | 14.8-30.6% |  |
|  |  | % within Provenance | 18.7% | 33.3% | 20.8% |
|  |  | Adjusted Residual | -2.8887 | 2.8887 |  |
|  |  | Pearson Chi-Square | 0.0039* | 0.0039* |  |
|  | IA | Count | 18 | 6 | 24 |
|  |  | Expected Count | 20.5 | 3.5 | 24.0 |
|  |  | % within Dating | 75.0% | 25.0% | 100.0% |
|  |  | 95% CI | 58.3-91.7% | 8.3-41.7% |  |
|  |  | % within Provenance | 4.1% | 8.0% | 4.6% |
|  |  | Adjusted Residual | -1.5050 | 1.5050 |  |
|  |  | Pearson Chi-Square | 0.1323 | 0.1323 |  |
| Total | | Count | 444 | 75 | 519 |
|  |  | Expected Count | 444,0 | 75.0 | 519.0 |
|  |  | % within Dating | 85.5% | 14.5% | 100.0% |
|  |  | 95% CI |  | 11.4-17.3% |  |
|  |  | % within Provenance | 100.0% | 100.0% | 100.0% |
| * significant after Bonferroni correction (p<0.0071) | | | | | |

Table S3.5.2: Pearson Chi-Square Test for provenance (“local”=individuals who show strontium ratios inside the range determined for the Northern pre-Alpine foreland in South Bavaria, non-local= individuals who show strontium ratios outside the range determined for the Northern pre-Alpine foreland in South Bavaria) vs. time period (EN=Early Neolithics, EMA=Early Middle Ages, LN=Late Neolithics, EBA= Early Bronze Age, LA=Late Antique, 500AD=around 500 AD , IA= Iron Age).

|  | Value | df | Asymptotic Significance (2-sided) |
| --- | --- | --- | --- |
| Pearson Chi-Square | 16.671^a^ | 6 | 0.011 |
| Likelihood Ratio | 17.260 | 6 | 0.008 |
| Linear-by-Linear Association | 14.185 | 1 | <0.001 |
| N of Valid Cases | 519 |  |  |
| a. 1 cells (7.1%) have expected count less than 5. The minimum expected count is 3.47. | | | |

## References

[1] Schoeninger MJ, DeNiro MJ. Nitrogen and carbon isotopic composition of bone collagen from marine and terrestrial animals. Geochim Cosmochim Acta. 1984; 48:625-639.

[2] Richards MP, Mellars PA. Stable isotopes and the seasonality of the Oronsay middens. Antiquity. 1998; 72(275):178-184.

[3] Lightfoot E, Liu X, Jones MK. Why move starchy cereals? A review of the isotopic evidence for prehistoric millet consumption across Eurasia. World Archaeology. 2013 Oct; 45(4):574-623.

[4] Schoeninger MJ, DeNiro MJ, Tauber H. Stable Nitrogen Isotope Ratios of Bone Collagen Reflect Marine and Terrestrial Components of Prehistoric Human Diet. Science. 1983 Jun 24; 220(4604):1381-1383.

[5] Hakenbeck SE, McManus E, Geisler H, Grupe G, O’Connell T. Diet and mobility in Early Medieval Bavaria: A study of carbon and nitrogen stable isotopes. Am J Phys Anthropol. 2010 Oct; 143(2):235-249.

[6] von Heyking K. Anthropologie einer frühstädtischen Randgruppe morphologische und archäometrische Untersuchung eines hoch- bis spätmittelalterlichen Armenhausgräberfeldes in Regensburg. Ludwig-Maximilians-Universität. 2012; 302 p.

[7] Sofeso C, Vohberger M, Wisnowsky A, Päffgen B, Harbeck M. Verifying archaeological hypotheses: Investigations on origin and genealogical lineages of a privileged society in Upper Bavaria from Imperial Roman times (Erding, Kletthamer Feld). In: Burger J, Kaiser E, Schier W, editors. Population dynamics in pre- and Early History. New Approaches by using Stable Isotopes and Genetics. Berlin: De Gruyter. 2012; 115-132.

[8] Strott N. Paläodemographie frühmittelalterlicher Bevölkerungen Altbaierns - Diachrone und allopatrische Trends. Ludwig-Maximilians-Universität. 2006; 331 p.

[9] Lee-Thorp JA. On Isotopes and Old Bones*. Archaeometry. 2008 Dec; 50(6):925-950.

[10] Bocherens H, Drucker D. Trophic level isotopic enrichment of carbon and nitrogen in bone collagen: case studies from recent and ancient terrestrial ecosystems. Int J Osteoarchaeol. 2003 Jan; 13(1–2):46-53.

[11] Hedges REM, Reynard LM. Nitrogen isotopes and the trophic level of humans in archaeology. J Archaeol Sci. 2007; 34:1240-1251.

[12] Ebersbach R. Von Bauern und Rindern. Eine Ökosystemanalyse zur Bedeutung der Rinderhaltung in bäuerlichen Gesellschaften als Grundlage zur Modellbildung im Neolithikum. Basler Beiträge zur Archäologie 15. Basel: Schwabe. 2002; 393 p.

[13] Ebersbach R. Glückliche Milch von glücklichen Kühen? Zur Bedeutung der Rinderhaltung in (neolithischen) Wirtschaftssystemen. In: Herrmann B, editor. Beiträge zum Göttinger Umwelthistorischen Kolloquium 2004–2006. Graduiertenkolleg Interdisziplinäre Umweltgeschichte. Göttingen: Universitätsverlag. 2007; 41-58.

[14] Willerding U. Die Landwirtschaft bei den Germanen und in den römischen Provinzen bis zur Völkerwanderungszeit - Ackerbau. In: Benecke N, Donat P, Grindmuth-Dallmer E, Willerding U, editors. Frühgeschichte der Landwirtschaft in Deutschland. Beier & Beran. 2003; 35-57.

[15] Fraser RA, Bogaard A, Charles M, Styring AK, Wallace M, Jones G, et al. Assessing natural variation and the effects of charring, burial and pre-treatment on the stable carbon and nitrogen isotope values of archaeobotanical cereals and pulses. J Archaeol Sci. 2013 Dec; 40(12):4754-4766.

[16] Knipper C, Peters D, Meyer C, Maurer AF, Muhl A, Schöne BR, et al. Dietary reconstruction in Migration Period Central Germany: a carbon and nitrogen isotope study. Archaeol Anthropol Sci. 2013 Mar; 5(1):17-35.

[17] Chisholm BS, Nelson DE, Schwarcz HP. Stable-Carbon Isotope Ratios as a Measure of Marine Versus Terrestrial Protein in Ancient Diets. Science. 1982 Jun 4; 216(4550):1131-1132.

[18] Richards MP, Hedges REM. Stable Isotope Evidence for Similarities in the Types of Marine Foods Used by Late Mesolithic Humans at Sites Along the Atlantic Coast of Europe. J Archaeol Sci. 1999 Jun; 26(6):717-722.

[19] Barrett JH, Orton D, Johnstone C, Harland J, Van Neer W, Ervynck A, et al. Interpreting the expansion of sea fishing in medieval Europe using stable isotope analysis of archaeological cod bones. J Archaeol Sci. 2011 Jul; 38(7):1516-1524.

[20] Fischer A, Olsen J, Richards M, Heinemeier J, Sveinbjornsdottir AE, Bennike P. Coast-inland mobility and diet in the Danish Mesolithic and Neolithic: evidence from stable isotope values of humans and dogs. J Archaeol Sci. 2007; 34:2125-2150.

[21] Richards MP, Fuller BT, Hedges REM. Sulphur isotopic variation in ancient bone collagen from Europe: implications for human palaeodiet, residence mobility, and modern pollutant studies. Earth Planet Sci Lett. 2001 Sep; 191(3–4):185-190.

[22] France R. Critical examination of stable isotope analysis as a means for tracing carbon pathways in stream ecosystems. Can J Fish Aquat Sci. 1995 Mar 1; 52(3):651-656.

[23] Finlay JC, Kendall C. Stable isotope tracing of temporal and spatial variability in organic matter sources to freshwater ecosystems. Stable Isotopes Ecol Environ Sci. 2007; 2:283-333.

[24] Guiry E. Complexities of Stable Carbon and Nitrogen Isotope Biogeochemistry in Ancient Freshwater Ecosystems: Implications for the Study of Past Subsistence and Environmental Change. Front Ecol Evol. 2019 Aug 21; 7:313.

[25] Finlay JC, Power ME, Cabana G. Effects of water velocity on algal carbon isotope ratios: implications for river food web studies. Limnol. Oceanogr. 1999; 44:1198-1203.

[26] Finlay JC. Patterns and controls of lotic algal stable carbon isotope ratios. Limnol. Oceanogr. 2004; 49:850-861.

[27] Drucker DG, Henry-Gambier D. Determination of the dietary habits of a Magdalenian woman from Saint-Germain-la-Rivière in southwestern France using stable isotopes. J Hum Evol. 2005 Jul; 49(1):19-35.

[28] Bocherens H, Drucker DG, Bonjean D, Bridault A, Conard NJ, Cupillard C, et al. Isotopic evidence for dietary ecology of cave lion (Panthera spelaea) in North-Western Europe: Prey choice, competition and implications for extinction. Quat Int. 2011 Dec; 245(2):249-261.

[29] Ambrose SH. Isotopic analysis of Paleodiets: Methodological and interpretive considerations. In: Sandford MK, editor. Investigation of Ancient Human Tissue: Chemical Analyses in Anthropology. Gordon and Breach Science Publishers. 1993; 59-130.

[30] Pate FD. Bone chemistry and paleodiet. J Archaeol Method Theory. 1994; 6:161-209.

[31] Fry B. Stable Isotope Ecology. New York: Springer New York. 2006; 308 p.

[32] Le Huray JD, Schutkowski H. Diet and social status during the La Tène period in Bohemia: Carbon and nitrogen stable isotope analysis of bone collagen from Kutná Hora-Karlov and Radovesice. J Anthropol Archaeol. 2005 Jun; 24(2):135-147.

[33] Zach B. Äcker und Gärten im frühmittelalterlichen Bayern. In: Haberstroh J, Heitmeier I, editors. Gründerzeit. Siedlung in Bayern zwischen Spätantike und Frühmittelalter. St. Ottilien: Eos. 2019; 205-218.

[34] Rösch M. The history of crops and crop weeds in south-western Germany from the Neolithic period to modern times, as shown by archaeobotanical evidence. Veg Hist Archaeobot. 1998; 7:109-125.

[35] Rösch M, Jacomet S, Karg S. The history of cereals in the region of the former Duchy of Swabia (Herzogtum Schwaben) from the Roman to the Post-medieval period: results of archaeobotanical research. Veget Hist Archaebot. 1992 Dec; 1(4): 193-231.

[36] Spurr MS. The Cultivation of Millet in Roman Italy. Pap Br Sch Rome. 1983 Nov; 51:1-15.

[37] Cernusak LA, Tcherkez G, Keitel C, Cornwell WK, Santiago LS, Knohl A, et al. Why are non-photosynthetic tissues generally ^13^C enriched compared with leaves in C3 plants? Review and synthesis of current hypotheses. Functional Plant Biol. 2009; 36(3):1-15.

[38] Merah O, Deléens E, Teulat B, Monneveux P. Associationbetween yield and carbon isotope discrimination value in differentorgans of durum wheat under drought. J Agron Crop Sci. 2002; 188:426-434.

[39] Ferrio JP, Voltas J, Araus JL. Use of carbon isotope composition in monitoring environmental changes. Manage Environ Qual: An Int J. 2003; 14(1):82-98.

[40] Heaton THE. Spatial, species, and temporal variations in the 13C/12C ratios of C3 plants: implications for palaeodiet studies. J Archaeol Sci. 1999; 26:637-649.

[41] Bocherens H. Preservation of isotopic signals (^13^C, ^15^N) in Pleistocene mammals. In: Ambrose SH, Katzenberg AM, editors. Biochemical approaches to paleodietary analysis. Advances in archaeological and museum science 5. New York: Kluwer Academic/Plenum. 2000; 65-88.

[42] Cerling TE, Harris JM. Carbon isotope fractionation between diet and bioapatite in ungulate mammals and implications for ecological and paleoecological studies. Oecologia. 1999 Aug 20; 120(3):347-363.

[43] Kohn MJ. Carbon isotope compositions of terrestrial C3 plants as indicators of (paleo)ecology and (paleo)climate. PNAS. 2010; 107:19691-19695.

[44] Stika HP.Römerzeitliche Pflanzenreste aus Baden-Württemberg. Materialh Archäol Bad-Württ 36. Stuttgart: Theiss. 1996; 207 p.

[45] Rösch M. New aspects of agriculture and diet of the early medieval period in central Europe: waterlogged plant material from sites in south-western Germany. Veget Hist Archaeobot. 2008 Dec; 17(S1):225-238.

[46] Alt KW, Müller C, Held P. Ernährungsrekonstruktion anhand stabiler Isotope von Kohlenstoff und Stickstoff an frühmittelalterlichen Bestattungen der Gräberfelder von Tauberbischofsheim-Dittigheim und Szólád. In: Drauschke J, Kislinger E, Kühtreiber K, Kühtreiber T, Scharrer- Liška G, Vida T, editors. Lebenswelten zwischen Archäologie und Geschichte: Festschrift für Falko Daim zu seinem 65. Geburtstag. Mainz: Römisch-Germanisches Zentralmuseum. 2018; 869-885.

[47] Craig-Atkins E, Jervis B, Cramp L, Hammann S, Nederbragt AJ, Nicholson E, et al. The dietary impact of the Norman Conquest: A multiproxy archaeological investigation of Oxford, UK. PLoS ONE. 2020 Jul 6; 15(7):e0235005.

[48] Nitsch EK, Humphrey LT, Hedges REM. Using stable isotope analysis to examine the effect of economic change on breastfeeding practices in Spitalfields, London, UK. Am J Phys Anthropol. 2011 Dec; 146(4):619-628.

[49] Eerkens JW, Berget AG, Bartelink EJ. Estimating weaning and early childhood diet from serial micro-samples of dentin collagen. J Archaeol Sci. 2011 Nov; 38(11):3101-3111.

[50] Schurr MR. Stable nitrogen isotopes as evidence for the age of weaning at the angel site: a comparison of isotopic and demographic measures of weaning age. J Archaeol Sci. 1997; 24:919-927.

[51] Millard AR. A model for the effect of weaning on nitrogen isotope ratios in humans. In: Goodfriend GA, Collins MJ, Fogel M, Macko SA, Wehmiller JF, editors. Perspectives in Amino Acid and Protein Geochemistry. Oxford: Oxford University Press. 2000; 51-59.

[52] Crowder KD, Montgomery J, Gröcke DR, Filipek KL. Childhood “stress” and stable isotope life-histories in Transylvania. Int J Osteoarchaeol. 2019; 29(4): 544-653.

[53] Waters-Rist AL, Katzenberg MA. The effect of growth on stable nitrogen isotope ratios in subadult bone collagen. Int J Osteoarchaeol. 2009; 20:172-191.

[54] Diefendorf AF, Mueller KE, Wing ScottL, Koch PL, Freeman KH. Global patterns in leaf ^13^C discrimination and implications for studies of past and future climate. PNAS. 2010 Mar 30; 107(13):5738-5743.

[55] Shishlina N, Sevastyanov V, Hedges REM. Isotope ratio study of Bronze Age samples from the Eurasian Caspian Steppes. In: Kaiser E, Burger J, Schier W, editors. Population Dynamics in Prehistory and Early History New Approaches Using Stable Isotopes and Genetics. Berlin-Boston: De Gruyter; 2012; 177-198.

[56] Wickham H. ggplot2: Elegant Graphics for Data Analysis. Springer: New York. 2016; 260 p.

[57] Wickham H, Chang W, Henry L, Pedersen TL, Takahashi K, Wilke C, et al. ggplot2: Create Elegant Data Visualisations Using the Grammar of Graphics. R package version 3.4.0. 2022; Available from: https://ggplot2.tidyverse.org.

[58] Tsutaya T, Yoneda M. Quantitative Reconstruction of Weaning Ages in Archaeological Human Populations Using Bone Collagen Nitrogen Isotope Ratios and Approximate Bayesian Computation. PLoS ONE. 2013; 8:e72327.

[59] Hedges R, Clement J, Thomas C, O'Connell T. Collagen turnover in the adult femoral mid-shaft: modeled from anthropogenic radiocarbon tracer measurements. Am. J. Anthopol. 2007; 133:808-816.

[60] Crowder KD, Montgomery J, Gröcke DR, Filipek KL. Childhood “stress” and stable isotope life-histories in Transylvania. Int J Osteoarchaeol. 2019; 29(4): 544-653.

[61] Dean MC, Scandrett AE. The relation between long-period incremental markings in dentine and daily cross-striations in enamel in human teeth. Arch Oral Biol. 1996 Mar; 41(3):233-241.

[62] Schour I, Poncher HG Rate of apposition of enamel and dentin, measured by the effect of acute fluorosis. Am J Dis Child. 1937; 54:757-776.

[63] Scharlotta I, Goude G, Herrscher E, Bazaliiskii VI, Weber AW. “Mind the gap”-Assessing methods for aligning age determination and growth rate in multi-molar sequences of dietary isotopic data. Am J Hum Biol. 2018 Sep; 30(5):e23163.

[64] Tsutaya T. Blurred time resolution of tooth dentin serial sections. Am J Phys Anthropol. 2020 Dec; 173(4):748-759.

[65] Czermak A, Fernández‐Crespo T, Ditchfield PW, Lee‐Thorp JA. A guide for an anatomically sensitive dentine microsampling and age‐alignment approach for human teeth isotopic sequences. Am J Phys Anthropol. 2020 Dec; 173(4):776-783.

[66] Beaumont J, Montgomery J, Buckberry J, Jay M. Infant mortality and isotopic complexity: New approaches to stress, maternal health, and weaning: Infant Mortality and Isotopic Complexity. Am J Phys Anthropol. 2015 Jul; 157(3):441-457.

[67] Beaumont J, Montgomery J. The Great Irish Famine: Identifying Starvation in the Tissues of Victims Using Stable Isotope Analysis of Bone and Incremental Dentine Collagen. Bondioli L, editor. PLoS ONE. 2016 Aug 10;11(8):e0160065.

[68] Craig-Atkins E, Jervis B, Cramp L, Hammann S, Nederbragt AJ, Nicholson E, et al. The dietary impact of the Norman Conquest: A multiproxy archaeological investigation of Oxford, UK. PLoS ONE. 2020 Jul 6; 15(7):e0235005.

[69] Fernández-Crespo T, Czermak A, Lee-Thorp JA, Schulting RJ. Infant and childhood diet at the passage tomb of Alto de la Huesera (north-central Iberia) from bone collagen and sequential dentine isotope composition. Int J Osteoarchaeol. 2018 Sep; 28(5):542-551.

[70] Velte M, Czermak A, Grigat A, Neidich D, Kropf E, Trautmann B et al. Exploring Early life histories. In prep.

[71] Mekota AM, Grupe G, Ufer S, Cuntz U. Serial analysis of stable nitrogen and carbon isotopes in hair: Monitoring starvation and recovery phases of patients suffering from anorexia nervosa. Rapid Commun Mass Spectrom. 2006; 20(10):1604-1610.

[72] Neuberger FM, Jopp E, Graw M, Püschel K, Grupe G. Signs of malnutrition and starvation: Reconstruction of nutritional life histories by serial isotopic analyses of hair. Forensic Sci Int. 2013; 226:22-32.

[73] D’Ortenzio L, Brickley M, Schwarcz H, Prowse T. You are not what you eat during physiological stress: Isotopic evaluation of human hair: Isotopic Evaluation of Human Hair. Am J Phys Anthropol. 2015 Jul; 157(3):374-388.

[74] Murphy C. Finding millet in the Roman world. Archaeol Anthropol Sci. 2016 Mar; 8(1):65-78.

[75] Hunt HV, Linden MV, Liu X, Motuzaite-Matuzeviciute G, Colledge S, Jones MK. Millets across Eurasia: chronology and context of early records of the genera Panicum and Setaria from archaeological sites in the Old World. Veg Hist Archaeobot. 2008; 17(Suppl 1): 5-18.

[76] Marinval P. Archaeobotanical data on millets (*Panicum miliaceum* and *Setaria italica*) in France. Rev Palaeobot Palynol. 1992 Sep; 73(1–4):259-270.

[77] van Dinter M. Living along the Limes: landscape and settlement in the Lower Rhine Delta during Roman and Early Medieval times. Utrecht: Utrecht University, Faculty of Geosciences, Department of Physical Geography. 2017; 224 p.

[78] Kooistra LI, van Dinter M, Dütting MK, van Rijn P, Cavallo C. Could the local population of the Lower Rhine delta supply the Roman army? Part 1: the archaeological and historical framework. J. Archaeol. Low Countries. 2013; 4(2):5-23.
